# Fractional-Order Traveling Wave Approximations for a Fractional-Order Neural Field Model

**DOI:** 10.3389/fncom.2022.788924

**Published:** 2022-03-24

**Authors:** Laura R. González-Ramírez

**Affiliations:** Escuela Superior de Física y Matemáticas, Instituto Politécnico Nacional, Mexico City, Mexico

**Keywords:** traveling wave, cortical wave propagation, fractional-order derivative, neural fields, memory effect

## Abstract

In this work, we establish a fractional-order neural field mathematical model with Caputo's fractional derivative temporal order α considering 0 < α < 2, to analyze the effect of fractional-order on cortical wave features observed preceding seizure termination. The importance of this incorporation relies on the theoretical framework established by fractional-order derivatives in which memory and hereditary properties of a system are considered. Employing Mittag-Leffler functions, we first obtain approximate fractional-order solutions that provide information about the initial wave dynamics in a fractional-order frame. We then consider the Adomian decomposition method to approximate pulse solutions in a wider range of orders and longer times. The former approach establishes a direct way to investigate the *initial* relationships between fractional-order and wave features, such as wave speed and wave width. In contrast, the latter approach displays wave propagation dynamics in different fractional orders for longer times. Using the previous two approaches, we establish approximate wave solutions with characteristics consistent with *in vivo* cortical waves preceding seizure termination. In our analysis, we find consistent differences in the initial effect of the fractional-order on the features of wave speed and wave width, depending on whether α <1 or α>1. Both cases can model the shape of cortical wave propagation for different fractional-orders at the cost of modifying the wave speed. Our results also show that the effect of fractional-order on wave width depends on the synaptic threshold and the synaptic connectivity extent. Fractional-order derivatives have been interpreted as the memory trace of the system. This property and the results of our analysis suggest that fractional-order derivatives and neuronal collective memory modify cortical wave features.

## 1. Introduction

Fractional-order derivatives have been employed to pursue a deeper understanding of different physical and biological processes, as these are thought to account for more realistic dynamic features. Fractional derivatives provide a framework in which the memory and hereditary properties of a system are taken into account (Ross, 1974; Podlubny, 1999; Ishteva, 2005; Ortigueira and Tenreiro Machado, 2015; Tarasov, 2018), in comparison with integer-order systems in which these features are not considered.

Although the exact physical interpretation of a fractional-order derivative remains an open problem, progress has been made in this direction. In Podlubny (2002), a geometrical interpretation of a fractional-order derivative was developed and it is suggested to express an inhomogeneity of the time scale. When applied in the temporal order, fractional derivatives may exert an influence on the effect of the delays of signals or history-dependent dynamics (Podlubny, 2002; Wang and Li, 2011). In Du et al. (2013), among others, it was established that the fractional-order derivative acts as an index of memory, meaning that the present state of a system is influenced by its past states. On the other hand, space fractional derivatives may describe the inhomogeneity of a medium. A space fractional derivative of order near two may represent anomalous diffusion, having non-local and possibly long-range interactions (Podlubny, 1999; Metzler and Klafter, 2000; Sokolov and Klafter, 2005; Chen et al., 2010).

In neuroscience, fractional-order derivatives have been applied to model dynamics of a single neuron in the Hodgkin-Huxley model (Baleanu et al., 2012; Nagy and Sweilam, 2014; Santamaria, 2015; Weinberg, 2015; Teka et al., 2016; Coutin et al., 2018), to the FitzHugh-Nagumo model (Pandir and Tandogan, 2013; Armanyos and Radwan, 2016), to model electrically coupled neuron systems (Moaddy et al., 2012), to bursting neuron models (Mondal et al., 2019), and to Cable equations (Henry et al., 2008; Langlands et al., 2009; Sweilam et al., 2014; Vitali et al., 2017; Yang et al., 2017), among others. In particular, in the Hodgkin-Huxley model, fractional derivatives of order less than 1 - which model the influence of the membrane potential memory- have been shown to affect the spiking diversity of the model (Santamaria, 2015; Weinberg, 2015; Teka et al., 2016).

Neural field models have been widely employed to describe mean neuronal population activity during hallucinations (Ermentrout and Cowan, 1979; Bressloff et al., 2001; Butler et al., 2012), the spreading of seizures (Connors and Amitai, 1993; Stefanescu et al., 2012; Zhao and Robinson, 2015; Kuhlmann et al., 2016; Jirsa et al., 2017; Proix et al., 2018), and many others (Coombes et al., 2014). To our knowledge, fractional-order neural field models have not been yet established in the literature. The novelties and contributions of this manuscript include a heuristic model motivation for a fractional-order neural field model, explicit approximated traveling wave solutions in the case of α ≈ 1, and explicit approximated wave solutions in the case of 0 < α < 2 employing a semi-analytical method for solving fractional-order differential equations, namely, the Adomian decomposition method. The explicit approximated solutions in the case of α ≈ 1 are in the form of finite sums of Mittag-Leffler functions, which provides a simpler scenario of closed-form solutions not usually obtained in fractional-order models. We also provide error estimates of such approximations. In the case of α ≈ 1^−^, our solutions converge to the solutions established in the first-order case. However, in the case of α ≈ 1^+^, there is no convergence to the first-order solution, and the usefulness of these approximations is restricted when considering long synaptic connectivity extents and low wave speeds. This characteristic agrees with the fractional-order derivative's memory interpretation, which asserts that both cases 0 < α ≤ 1 and 1 < α < 2 are considerably different. The obtained error estimates for each case motivate our work. By considering the Adomian decomposition method, we present approximated solutions in the form of power series decomposition and extend the approximated solutions to fractional-order of 0 < α < 2. We also provide error estimates of our solutions.

The primary goal of the manuscript is to provide a first investigation toward understanding the effect of fractional-order on wave propagation features. We claim that incorporating fractional-order derivatives into neural fields is essential. Realistic features can build more sensitive models of neuronal activity, particularly the potential incorporation of neuronal collective memory into neural field models. The primary motivation for incorporating a fractional-order approach into the modeling of pattern formation is to enlarge our understanding of wave propagation in a more realistic setting, where past dynamics might influence an effect. Also, to compare the possible outcomes and differences in the modeling of standard first-order features, as the results of fractional-order influence on single neuron models have shown the existence of ample dynamics. Our results are consistent in the exhibited waves and suggest different initial characteristics of the system's traveling wave solutions considering different fractional orders. Thus, the effect of the collective memory of the neuronal population due to the fractional derivative approach determines the features of the wave solutions.

The work in this manuscript is developed as follows. In Section 2, we review the wave features observed in *in vivo* clinical recordings preceding seizure termination that are found in the literature and establish explicit traveling wave solutions in the first-order case. In Section 3, we establish the approximate traveling wave solutions for values of α ≈ 1 and analyze the effect of neuronal collective memory on wave features by utilizing these approximations. In Section 3.5, we also establish the approximate wave solutions employing the Adomian decomposition method and analyze the wave features under this approach. Finally, in Section 4, we discuss the conclusions of this work and future work to be developed. To facilitate the visualization of the manuscript, we refer to the terms of our approximate solutions to the Supplementary Material. Since the main objective of this manuscript is to provide information related to wave propagation features, the manuscript is structured containing the relevant model motivation, results, and conclusions from analyzing the properties of traveling wave solutions under a fractional-order effect. The mathematical formalism, the details of the approximated solutions, and the error estimates appear in the Supplementary Material. In the Supplementary Material, we also provide a background for fractional calculus, discuss the memory interpretation of the Caputo fractional-order derivative, provide error estimates of our explicit Mittag-Leffler approximations, and develop the details behind the Adomian decomposition method described in Section 3.5.

## 2. Materials and Methods

### 2.1. Neural Field Models and Cortical Wave Propagation

In this section, we review the existence of traveling wave solutions of first-order neural field models. We establish a choice of parameters that support wave propagation with features consistent with *in vivo* wave dynamics. In this manuscript, we focus on modeling wave features observed in human clinical recordings reported in González-Ramírez et al. (2015), in particular, wave speeds varying from 80 μm/ms to 500 μm/ms, and wave widths varying from 1, 000 to 5, 000 μm. These values are in agreement with similar studies found in the literature (Chervin et al., 1988; Wadman and Gutnick, 1993; Golomb and Amitai, 1997). When necessary, we analyze features outside but close to these ranges-of-interest.

We consider a voltage-based neural field model with a linear adaptation term (Ermentrout, 1998; Pinto and Ermentrout, 2001). This model is based on the assumption that a presynaptic membrane potential, *V*, is converted into a firing rate by a convenient firing rate function *S*(*V*). Further assumptions are made to ignore processes, such as axonal delays, release of neurotransmitters, synaptic facilitation, dendritic architecture, among others, in order for the synaptic input, due to the synaptic interactions on a postsynaptic neuron, to be described by a convenient integral equation. To simplify this integral equation, it is assumed that the postsynaptic potential is mainly determined by the properties of the postsynaptic membrane and that it is modeled in terms of sums and powers of exponential functions. In this scenario, it is considered that the postsynaptic cell membrane behaves as an ideal capacitor; thus, that a first-order differential equation can be derived to describe the postsynaptic membrane potential. Considering a mean field approach and a continuum limit in the number of neurons of the previous system, a neural field model can be established to describe the mean features of neuronal populations. In this work, we consider a single population of neurons together with a linear bulk adaptation term (Pinto and Ermentrout, 2001), accounting for multiple processes (such as synaptic adaptation) and preventing activity from remaining excited. In this neural field model, there is a spatial convolution term that is employed to describe distance-dependent synaptic interactions. We will further comment on the details behind this model derivation when we motivate the fractional-order neural field model in the following section. The first order neural field voltage-based model with a linear adaptation term is determined by:


(1) $$\begin{array}{l} \begin{array}{c} D_{t}u\left(x,t\right)=-u\left(x,t\right)+\int_{-\infty }^{\infty }g\left(x-y\right)H\left(u\left(y,t\right)-k\right)dy-\beta q\left(x,t\right)\\ D_{t}q\left(x,t\right)=\epsilon u\left(x,t\right)-\epsilon q\left(x,t\right). \end{array} \end{array}$$

Here, *D*_*t*_ denotes the derivative with respect to *t*. The variable *u*(*x, t*) accounts for a mean synaptic input and the variable *q*(*x, t*) accounts for a linear adaptation term, both measured at position *x* and time *t*. The convolution term represents the inputs due to synaptic interactions. The kernel of the convolution is a symmetric weight function *g*(*x*) = *g*(−*x*) that monotonically decreases for *x*≥0. We choose an exponential kernel, $g\left(x\right)=\frac{1}{2\sigma }e^{-\frac{|x|}{\sigma }}$, where σ>0 denotes the extent of the synaptic connectivity, to provide concrete examples of wave solutions and to extend our notion of wave solutions to the fractional-order case. The function *H*(*x*) denotes a Heaviside function that is activated when the activity reaches a synaptic threshold, denoted by *k*. That is, *H*(*x*) = 1 for *x*≥*k* and *H*(*x*) = 0 if *x*<*k*. The parameter β denotes the strength of the adaptation term. The parameter ϵ <1 represents the decay rate parameter for the linear adaptation term, which we assume occurs more slowly than the synaptic input. All parameters are assumed to be positive. The units for the variables and parameters are as follows. The variables *u* and *q*, the strength of adaptation and the synaptic threshold are dimensionless. The synaptic connectivity extent, σ, has units of μm. The wave speed is measured in μm/ms and the wave width is measured in μm.

Traveling wave solutions of this model, which move with a fixed shape and constant speed *c*, have been established and extensively studied (Ermentrout, 1998; Pinto and Ermentrout, 2001; Bressloff, 2012; Coombes et al., 2014). Here, we provide a sketch of the derivation of such solutions (for details, see the Supplementary Material). To obtain explicit traveling wave solutions, we change coordinates into the moving frame (*z, t*), where *z* = *x* + *ct*, and look for stationary solutions in this system. We assume that the stationary solutions are pulse solutions that cross the synaptic threshold *k* at exactly two points: at *z* = *w*_0_ and *z* = *w*, so that the super-threshold activity region is determined by *w*_0_ ≤ *z* ≤ *w*. Given the fact that the traveling wave solutions are translationally invariant, we assume that *w*_0_ = 0. Using the variation of parameters formula, we obtain the traveling wave solutions under the traditional integer-order derivative setting:


(2) $$\begin{array}{l} \begin{array}{c} u\left(x,t\right)=\left(\frac{\epsilon -1+\sqrt{{\left(\epsilon -1\right)}^{2}-4\epsilon \beta }}{2c\sqrt{{\left(\epsilon -1\right)}^{2}-4\epsilon \beta }}\right)e^{\lambda_{+}\left(x+ct\right)}\\ \times \int_{-\infty }^{x+ct}e^{-s\lambda_{+}}\left(\int_{0}^{w}g\left(s-y\right)dy\right)ds\\ -\left(\frac{\epsilon -1-\sqrt{{\left(\epsilon -1\right)}^{2}-4\epsilon \beta }}{2c\sqrt{{\left(\epsilon -1\right)}^{2}-4\epsilon \beta }}\right)e^{\lambda_{-}\left(x+ct\right)}\\ \times \int_{-\infty }^{x+ct}e^{-s\lambda_{-}}\left(\int_{0}^{w}g\left(s-y\right)dy\right)ds, \end{array} \end{array}$$

and


(3) $$\begin{array}{l} \begin{array}{c} q\left(x,t\right)=\left(\frac{\epsilon }{c\sqrt{{\left(\epsilon -1\right)}^{2}-4\epsilon \beta }}\right)e^{\lambda_{+}\left(x+ct\right)}\\ \times \int_{-\infty }^{x+ct}e^{-s\lambda_{+}}\left(\int_{0}^{w}g\left(s-y\right)dy\right)ds\\ -\left(\frac{\epsilon }{c\sqrt{{\left(\epsilon -1\right)}^{2}-4\epsilon \beta }}\right)e^{\lambda_{-}\left(x+ct\right)}\\ \times \int_{-\infty }^{x+ct}e^{-s\lambda_{-}}\left(\int_{0}^{w}g\left(s-y\right)dy\right)ds, \end{array} \end{array}$$

where


(4) $$\begin{array}{l} \lambda_{\pm }=-\frac{\epsilon +1}{2c}\pm \frac{\sqrt{{\left(\epsilon -1\right)}^{2}-4\epsilon \beta }}{2c}. \end{array}$$

The procedure to obtain the traveling wave solutions (Equations 2,3) is fully established in Section 2 of the Supplementary Material.

To simplify our analysis throughout the manuscript, we assume that the parameters ϵ and β satisfy the inequality (ϵ−1)^2^ − 4 ϵ* β* > 0; thus, we focus on the real eigenvalue case. The traveling wave solutions (Equations 2, 3) can be simplified to be written as piecewise continuous solutions. The simplified traveling wave solutions are:


(5) $$\begin{array}{l} u_{⋆}(x,t)=\left\{ \begin{array}{l} A_{u}e^{\frac{x+ct}{\sigma }}-A_{u}e^{\frac{x+ct-w}{\sigma }}\\ \text{ if }x+ct\leq 0\\ B_{u}e^{\lambda_{+}(x+ct)}+C_{u}e^{\lambda_{-}(x+ct)}\\ \;+D_{u}e^{-\frac{x+ct}{\sigma }}+E_{u}e^{\frac{x+ct-w}{\sigma }}+F_{u}\\ \text{ if }0<x+ct\leq w\\ G_{u}e^{\lambda_{+}(x+ct)}-G_{u}e^{\lambda_{+}(x+ct-w)}\\ \;+H_{u}e^{\lambda_{-}(x+ct)}-H_{u}e^{\lambda_{-}(x+ct-w)}\\ \;+I_{u}e^{-\frac{x+ct}{\sigma }}+J_{u}e^{-\frac{x+ct-w}{\sigma }}\\ \text{ if }x+ct>w, \end{array}\right. \end{array}$$

and


(6) $$\begin{array}{l} q_{⋆}(x,t)=\left\{ \begin{array}{l} A_{q}e^{\frac{x+ct}{\sigma }}-A_{q}e^{\frac{x+ct-w}{\sigma }}\\ \text{ if }x+ct\leq 0\\ B_{q}e^{\lambda_{+}(x+ct)}+C_{q}e^{\lambda_{-}(x+ct)}\\ \;+D_{q}e^{-\frac{x+ct}{\sigma }}+E_{q}e^{\frac{x+ct-w}{\sigma }}+F_{q}\\ \text{ if }0<x+ct\leq w\\ G_{q}e^{\lambda_{+}(x+ct)}-G_{q}e^{\lambda_{+}(x+ct-w)}\\ \;+H_{q}e^{\lambda_{-}(x+ct)}-H_{q}e^{\lambda_{-}(x+ct-w)}\\ \;+I_{q}e^{-\frac{x+ct}{\sigma }}+J_{q}e^{-\frac{x+ct-w}{\sigma }}\\ \text{ if }x+ct>w. \end{array}\right. \end{array}$$

The coefficients of the previous expressions depend on the different model parameters and are fully established in the Supplementary Material. The existence of wave solutions is determined by the matching conditions:


(7) $$\begin{array}{l} u\left(0,t\right)=k,\;\text{and }u\left(w,t\right)=k. \end{array}$$

**Figure 1:**
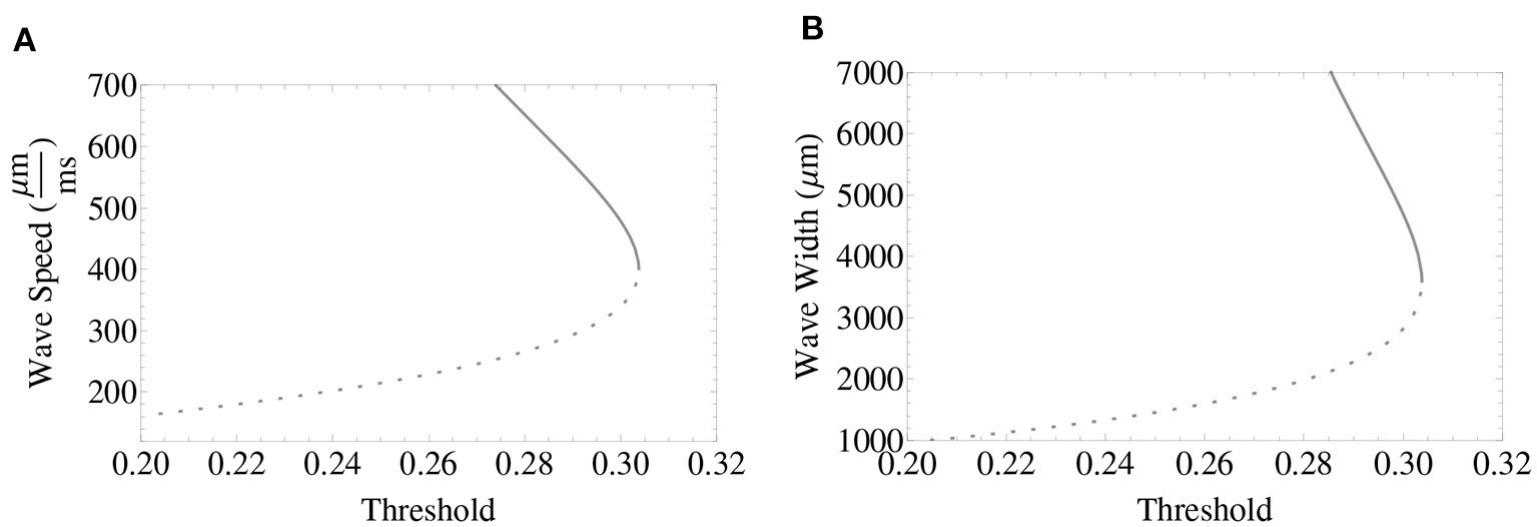
Traveling wave solutions for the first-order neural field model with wave features consistent with *in vivo* clinical features. **(A)** Wave speed determined by synaptic threshold *k*. **(B)** Wave width determined by synaptic threshold *k*. The lower branch (dashed gray curve) consists of unstable waves and the upper branch (gray curve), of stable waves colliding in a saddle-node bifurcation as the synaptic threshold is increased. Parameters used for these plots: ϵ = 0.1, β = 1 and σ = 1, 000 μm.

In Figure 1, we provide plots of the relationship among wave width, wave speed, and synaptic threshold, together with a choice of parameters in which the model (System 1) supports wave features found in the range-of-interest. The curves shown consist of a lower branch of unstable waves and an upper branch of stable waves. The linear and nonlinear stability of the pulse solutions have been fully addressed in Pinto and Ermentrout (2001); Coombes and Owen (2004); Pinto et al. (2005); Sandstede (2007), and Kapitula et al. (2004). In this work, we focus our efforts mostly on understanding the behavior of fractional-order wave solutions lying in the upper branch motivated by the stability of the traveling wave solutions in the integer-order case, and because biologically it is more realistic that stable wave solutions model the propagation of cortical wave activity. However, to our knowledge, the stability of wave solutions of fractional-order neural fields has yet to be addressed.

## 3. Results

### 3.1. Fractional-Order Neural Field Model Motivation

In this section, we consider an extension of the model (System 1) into a fractional-order setting. The motivation behind this approach lies in the approximation modeling, where the cell membrane is modeled as an ideal capacitor. In a more realistic setting, capacitors can entertain losses and frequency variation of the capacitance. A fractional-order approach has been suggested to better describe these complicated dynamics (Westerland and Ekstam, 1994). Therefore, in this fractional setting, more complicated dynamics of the postsynaptic membrane potential can be considered. In particular, a fractional-order approach is suggested to model memory events of the capacitance (0 < α <1) (Westerland and Ekstam, 1994) or plausible fractional-order relaxation-oscillation behavior (1 < α < 2) (Tofighi, 2003).

We consider a traditional neural networks heuristic derivation (Ermentrout, 1998) and we allow the indices *j* and *i* to denote a presynaptic and a postsynaptic neuron, respectively. In this way, the membrane potential of a presynaptic cell and a postsynaptic cell is denoted by *V*_*j*_(*t*) and *V*_*i*_(*t*), respectively. As previously mentioned, we assume that the potential in each cell, *V*, has been converted into a firing rate by a convenient firing rate function *S*(*V*). In a traditional integer-order setting, it is assumed that the capacitance current, *I*_*c*_, and the membrane potential, *V*_*M*_, are related by:


(8) $$\begin{array}{l} I_{c}=C_{M}\frac{dV_{M}}{dt}, \end{array}$$

where the membrane capacitance is denoted by *C*_*M*_. We assume that an action potential of a presynaptic cell affects the postsynaptic cell by means of a postsynaptic potential, *PSP*_*ij*_(*t* − *s*), where *t* denotes the measured time and *s* = {*t*_1_, *t*_2_, ...} describes the spike times of the presynaptic neuron. We also consider that there are no delays due to the distance traveled along the axon or due to the geometrical structure of the axon. We assume that the postsynaptic potential adds up linearly and we account for all the possible times to determine to total potential at the soma of cell *i*. We define the total potential due to cell *j* at the soma of cell *i* at time *t*, *G*_*i, j*_(*t*), as:


(9) $$\begin{array}{l} G_{i,j}\left(t\right):=\underset{k}{\sum }PSP_{ij}\left(t-t_{k}\right), \end{array}$$

where the index *k* denotes the total number of spikes considered. Considering the instantaneous firing rate of the presynaptic cell, *S*_*j*_(*V*), it is possible to rewrite the above expression as:


(10) $$\begin{array}{l} G_{i,j}\left(t\right)=\int_{-\infty }^{t}PSP_{ij}\left(t-s\right)S_{j}\left(V_{j}\left(s\right)\right)ds. \end{array}$$

Considering different presynaptic cells, we obtain the total potential to the postsynaptic cell as:


(11) $$\begin{array}{l} V_{i}\left(t\right)=\underset{j}{\sum }\int_{-\infty }^{t}PSP_{ij}\left(t-s\right)S_{j}\left(V_{j}\left(s\right)\right)ds. \end{array}$$

In a traditional voltage-based formulation, it is also assumed that the postsynaptic potential is solely determined by the properties of the postsynaptic cell, that is *PSP*_*ij*_(*t*) = *w*_*ij*_*PSP*_*i*_(*t*), for convenient weights *w*_*ij*_. In addition, it is assumed that these postsynaptic potentials are determined by the sums and powers of exponential functions. The inverse of a convenient linear integral operator with exponential kernel is a first-order constant coefficient differential operator. The latter fact can be used to further simplify Equation (11) to obtain:


(12) $$\begin{array}{l} \tau_{i}\frac{dV_{i}\left(t\right)}{dt}+V_{i}=\underset{j}{\sum }w_{ij}S_{j}\left(V_{j}\left(s\right)\right). \end{array}$$

In the latter scenario, it is assumed that the postsynaptic membrane behaves as an ideal capacitor with zero losses and constant capacitance. Also, the time constant of the model, τ_*i*_, is determined by the membrane properties of the postsynaptic cell.

In a more realistic physical setting, the losses of capacitors producing a capacitance frequency variation can be taken into account. This has been modeled as a fractional-order model (Westerland and Ekstam, 1994) as:


(13) $$\begin{array}{l} I_{c}\left(t\right)={C{\;}_{\alpha }}_{a}D_{t}^{\alpha }V_{c}\left(t\right), \end{array}$$

where *I*_*c*_(*t*) is the total current, *V*_*c*_(*t*) is the voltage, *C*_α_ is the capacitance and ${\;}_{a}D_{t}^{\alpha }$ is the Caputo's fractional-order derivative operator. The fractional-order α can be considered in the range of 0 < α < 2, as orders of α>2 determine inductive elements that are no longer capacitive. When α = 1, we recover the ideal capacitor setting used to derive Equation (12). When 0 < α <1, a non-ideal capacitance is modeled with memory events of the capacitance being described with a power law attenuation (Westerland and Ekstam, 1994). This setting has been utilized to model the ionic conductances in the Hodgkin-Huxley model in Santamaria (2015). On the other hand, by means of Fourier transforming Equation (13), it is possible to determine that the impedance of the capacitor in the case of 1 < α < 2 consists of a negative resistance (Jiang et al., 2020), allowing the plausibility of oscillations and sustained large currents at specific frequencies. In particular, the latter case can be employed to model fractional-order relaxation oscillation behavior (Tofighi, 2003). Both scenarios have interesting motivations for modeling the dynamics of the postsynaptic membrane potential established in Equation (12) under a fractional-order perspective. In this fractional-order scenario, it is also necessary to consider a more general form for the postsynaptic potentials. We consider again the total potential to the postsynaptic cell (Equation 11):


(14) $$\begin{array}{l} V_{i}\left(t\right)=\underset{j}{\sum }\int_{-\infty }^{t}PSP_{ij}\left(t-s\right)S_{j}\left(V_{j}\left(s\right)\right)ds. \end{array}$$

We assume that the postsynaptic potential is solely determined by the properties of the postsynaptic cell, that is *PSP*_*ij*_(*t*) = *w*_*ij*_*PSP*_*i*_(*t*), for convenient weights *w*_*ij*_. In addition, it is now assumed that these postsynaptic potentials are determined by the sums and powers of Mittag-Leffler functions, *PSP*_*ij*_(*t*) = *M*(*t*). The definition of a two-parameter Mittag-Leffler function is $E_{\alpha ,\beta }\left(z\right)=\sum_{k=0}^{\infty }\frac{z^{k}}{\Gamma \left(\alpha k+\beta \right)}$ where α>0 and β>0. We note that the exponential function is solely a particular case of a Mittag-leffler function $E_{1,1}\left(\lambda t\right)=e^{\lambda t}$ (for more details and properties of Mittag-Leffler functions see Podlubny, 1999). In this scenario, we obtain:


(15) $$\begin{array}{l} V_{i}\left(t\right)=\underset{j}{\sum }\int_{-\infty }^{t}M\left(t-s\right)S_{j}\left(V_{j}\left(s\right)\right)ds. \end{array}$$

Considering 0 < α ≤ 1, it can be proven (Podlubny, 1999; Bonilla et al., 2007) that:


(16) $$\begin{array}{l} {\;}_{a}D_{t}^{\alpha }\int_{a}^{t}M(t-s)u(s)ds=u(t), \end{array}$$

where *M*(*t*) is a convenient Mittag-Leffler kernel, e.g., $M\left(t\right)=t^{\alpha -1}E_{\alpha ,\alpha }\left(t^{\alpha }\right)$, and ${\;}_{a}D_{t}^{\alpha }$ is the Caputo's fractional-order derivative of order α that is defined as:


(17) $$\begin{array}{l} {\;}_{a}D_{t}^{\alpha }f(t)=\frac{1}{\Gamma (1-\alpha )}\int_{a}^{t}\frac{f^{\prime }(\tau )}{{\left(t-\tau \right)}^{\alpha }}d\tau . \end{array}$$

For more details and properties of the Caputo's fractional-order derivative see the Supplementary Material. Considering convenient choices of Mittag-Leffler functions it is also possible to extend Equation (16) to the case of 1 < α ≤ 2. Equation (16) can be used to further simplify Equation (14) to obtain a fractional-order system as:


(18) $$\begin{array}{l} {\tau_{i}}_{a}D_{t}^{\alpha }V_{i}+V_{i}=\underset{j}{\sum }w_{ij}S_{j}\left(V_{j}\left(s\right)\right). \end{array}$$

Considering the previous motivation we now propose a fractional-order neural field model as:


(19) $$\begin{array}{l} \begin{array}{c} D_{t}^{\alpha }u\left(x,t\right)=-u\left(x,t\right)+\int_{-\infty }^{\infty }g\left(x-y\right)H\left(u\left(y,t\right)-k\right)dy-\beta q\left(x,t\right)\\ D_{t}^{\alpha }q\left(x,t\right)=\epsilon u\left(x,t\right)-\epsilon q\left(x,t\right), \end{array} \end{array}$$

where $D_{t}^{\alpha }$ denotes the Caputo's fractional derivative of order α and where we have fixed the lower bound to *a* = 0:


(20) $$\begin{array}{l} D_{t}^{\alpha }f\left(t\right):{=}_{0}D_{t}^{\alpha }f\left(t\right)=\frac{1}{\Gamma \left(n-\alpha \right)}\int_{0}^{t}\frac{f^{n}\left(\tau \right)}{{\left(t-\tau \right)}^{\alpha +1-n}}d\tau , \end{array}$$

for a convenient function *f* and *n* ∈ ℤ^+^ so that *n* − 1 < α ≤ *n* and 0 < α < 2. We note that when α = 1 we recover System (1). In the Supplementary Material, we provide the mathematical formalism behind Caputo's fractional-order derivative and describe its memory interpretation. We are particularly interested in establishing traveling wave solutions in this fractional-order neural field model with features of speed and width within the range of cortical wave propagation.

Explicit traveling wave solutions of fractional-order systems have been established employing the complex transformation method and considering a fractional moving frame: *z* = *x* + *ct*^α^. However, these solutions rely on the use of a chain rule for fractional derivatives, which is known not to be valid (Tarasov, 2016). To our knowledge there is no general method for obtaining explicit closed-form wave solutions in fractional-order system, with a bounded lower limit definition, unless a modified chain rule or transformation is used. In this section, we extend the initial existence of approximate traveling wave solutions for fractional-order equations with a bounded lower limit derivative definition with order α ≈ 1 by making use of our explicit wave solutions in the integer-order case. In this way, we analyze the initial dynamics of wave solutions in a fractional-order frame starting from a first-order solution. In the Supplementary Material, we establish error estimates for our approximations that depend on the features of wave speed *c*, synaptic connectivity range σ, fractional-order α, position *x*, and time *t*. Therefore, these solutions only provide an insight of the *initial* wave dynamics in the fractional-order frame. This first approach is possible due to three factors: (i) the explicit solutions (Equations 5, 6) established as finite sums of exponential functions, (ii) the choice of the Heaviside function to describe the input of synaptic interaction (System 19), and (iii) the choice of a convenient kernel to describe the synaptic connectivity in each of the fractional-order neural field models. Our solutions can be verified by direct substitution into the fractional-order system and by using the derivative approximations established herein. We divide our analysis into two cases: α ≈ 1^−^ and α ≈ 1^+^. According to the memory interpretation described in the Supplementary Material, these two cases have a significantly distinct memory effect. For values of α ≈ 1^−^, we have less neuronal memory effect (transport-like memory from the first-order derivative), and for values α ≈ 1^+^, we have more neuronal memory effect (diffusive-like memory from the second-order derivative).

The first approach that we will present here is based on the natural extensions of exponential functions by Mittag-Leffler functions. This approach will provide explicit closed formulations that permit a direct investigation of the relationship between wave width, wave speed, and synaptic threshold, as well as a convenient analysis of the effect of fractional-order α on the different model parameters. On the other hand, some of the disadvantages of this approach are that the method restricts the use of a particular kernel in the synaptic connectivity term and that our analysis is only valid for α ≈ 1. Nevertheless, in the Supplementary Material we show that for relatively small times (*t* = 0.1), a good approximation might be obtained for fractional orders relatively far from order one (α = 0.9).

### 3.2. Approximate Traveling Wave Solutions With α ≈ 1^−^

In order to establish the approximate traveling wave solutions, we consider the following fractional-order equation:


(21) $$\begin{array}{l} D_{t}^{\alpha }w\left(t\right)=\lambda w\left(t\right). \end{array}$$

The previous equation can be solved by means of Fourier transform obtaining solutions determined by Mittag-Leffler functions (Podlubny, 1999). For values of 0 < α <1, we find that the solutions of Equation (21) are of the form:


(22) $$\begin{array}{l} w\left(t\right)=AE_{\alpha ,1}\left(\lambda t^{\alpha }\right), \end{array}$$

where $E_{\alpha ,1}\left(\lambda t^{\alpha }\right)$ is a two-parameter Mittag-Leffler function and *A* is a constant.

Motivated by Equation (21), we investigate the behavior of the fractional derivative of Mittag-Leffler functions in the fractional moving frame determined by *z* = *x* + *ct*^α^, obtaining:


(23) $$\begin{array}{l} D_{t}^{\alpha }E_{\alpha ,1}\left(\frac{x+ct^{\alpha }}{\sigma }\right)\approx \frac{c}{\sigma }E_{\alpha ,1}\left(\frac{x+ct^{\alpha }}{\sigma }\right), \end{array}$$

for values of α ≈ 1^−^. In general, the chain rule is not valid for fractional derivatives. This implies that Equation (23) is an approximation where the absolute error is established in terms of Mittag-Leffler functions. In Section 4 of the Supplementary Material we establish the procedure to obtain the approximation determined by Equation (23). In this case, as α → 1^−^, inequality determined by Equation (23) tends to an equality. Our estimates are better suited for considering narrower waves, longer synaptic connectivity ranges σ, values of α sufficiently close to 1 and small times.

We consider System (19) together:


(24) $$\begin{array}{l} g_{L}(x)=\frac{1}{2\sigma }\frac{d}{du}\left(E_{\alpha ,1}u\right){|}_{u=-\frac{|x|}{\sigma }}. \end{array}$$

It can be proven that *g*_*L*_(*x*) → *g*(*x*) as α → 1^−^, thus as α → 1^−^ we recover the integer-order neural field model (System 1).

By conveniently replacing the exponential functions in Equations (5) and (6) by Mittag-Leffler functions, we establish approximate fractional traveling wave solutions that can be verified by a direct substitution into the model (System 19):


(25) $$\begin{array}{l} u_{⋆L}\left(x,t\right), \end{array}$$

and


(26) $$\begin{array}{l} q_{⋆L}\left(x,t\right). \end{array}$$

To facilitate the visualization of the manuscript, the explicit description of the previous two equations are fully established in the Supplementary Material as Equations (S22) and (S23). In section 3.4, we will further analyze the matching conditions that determine the existence of the wave solutions (Equations 25,26), as well as the neuronal collective memory effect on wave features.

### 3.3. Approximate Traveling Wave Solutions With α ≈ 1^+^

In a similar fashion, we consider 1 < α < 2 and the following eigenvalue equation:


(27) $$\begin{array}{l} D_{t}^{\alpha }w\left(t\right)=\lambda w\left(t\right). \end{array}$$

Solutions to Equation (27) can be obtained by means of Fourier transform and are determined by:


(28) $$\begin{array}{l} w\left(t\right)=AE_{\alpha }\left(\lambda t^{\alpha }\right)+BtE_{\alpha ,2}\left(\lambda t^{\alpha }\right), \end{array}$$

where *A* and *B* are constants. We establish estimates of the fractional derivative of Mittag-Leffler functions in the fractional frame:


(29) $$\begin{array}{l} \begin{array}{c} D_{t}^{\alpha }\left(E_{\alpha ,1}\left(\frac{x+ct^{\alpha }}{\sigma }\right)+\left(\frac{x+ct^{\alpha }}{\sigma }\right)E_{\alpha ,2}\left(\frac{x+ct^{\alpha }}{\sigma }\right)\right)\\ \;\approx \frac{c}{\sigma }\left(E_{\alpha ,1}\left(\frac{x+ct^{\alpha }}{\sigma }\right)+\left(\frac{x+ct^{\alpha }}{\sigma }\right)E_{\alpha ,2}\left(\frac{x+ct^{\alpha }}{\sigma }\right)\right). \end{array} \end{array}$$

The error of the estimate determined by Equation (29) is established in terms of Mittag-Leffler functions in Section 4 of the Supplementary Material. We remark that in this case, as α → 1^+^, the inequality determined by Equation (29) does not converge to an equality. However, for sufficiently long connectivity extent and low speeds, the absolute error of our estimate is sufficiently small and this motivates our study. For details, please see the Supplementary Material.

Consider System (19) together with the following kernel:


(30) $$\begin{array}{l} g_{R}(x)=\frac{1}{4\sigma }\frac{d}{du}\left[E_{\alpha ,1}u\right]{|}_{u=-\frac{|x|}{\sigma }}+\frac{1}{4\sigma }\frac{d}{du}\left[uE_{\alpha ,2}u\right]{|}_{u=-\frac{|x|}{\sigma }}. \end{array}$$

It can be proven that *g*_*R*_(*x*) → *g*(*x*) as α → 1^+^; thus, we also recover Equation (1) as α → 1^+^. By replacing the exponential functions in Equations (2) and (3) by convenient choices of Mittag-Leffler functions, we obtain the approximate traveling wave solutions that can be verified by a direct substitution into the model:


(31) $$\begin{array}{l} u_{⋆R}\left(x,t\right), \end{array}$$

and


(32) $$\begin{array}{l} q_{⋆R}\left(x,t\right). \end{array}$$

The explicit description of the previous equations are fully described in the Supplementary Material [Equations (S24) and (S25)]. In there, we also show the error estimates for Equations (23) and (29) finding a better agreement in the case of 0 < α <1. This is consistent with the memory interpretation of the fractional-order derivative.

### 3.4. On the Effect of Fractional-Order on Wave Features

We now explore the existence conditions determined by Equation (7) on the wave solutions (Equations 25, 31). Because our theoretical results are based on chain rule approximations (Equations 23, 29), for the present we limit our analysis to fractional-orders α ≈ 1. We are particularly interested in extending the modeling of cortical wave features using fractional-order neural field models and analyzing the effect due to fractional-order on wave features when considering small times (*t* = 0.1). The estimations and projections in this first approach depend on the absolute error estimates determined for Equations (23) and (29), which are fully established in the Supplementary Material. The error estimates depend on fractional-order α, synaptic connectivity σ, wave speed *c*, distance *x*, and time *t*. For this approach, we only consider relatively long synaptic connectivity ranges (σ = 1, 000 μm, and σ = 1, 500 μm), in that our error estimates are suited for these values. The synaptic connectivity ranges that have been used are contained within reported ranges -of 40 μm to 2mm- of synaptic connectivity measurements (Braitenberg and Schuz, 1998; Linden et al., 2011; Peyrache et al., 2012). We consider values of α ≈ 1 and, for ease of visualization, we project the memory effect due to the fractional derivative for values of α distant from 1 (α = 0.9 or α = 1.1). In our analysis, we find a consistent behavior of solutions for different fractional derivatives orders in each of the two cases (α ≈ 1^−^ and α ≈ 1^+^). Due to the nature of our approach, we obtain best estimates for the lower branch of waves, which is known to consist of unstable waves. However, we are also able to gain an insight into a portion of the upper branch, which is known to consist of stable waves that are relevant to describe cortical wave propagation. Therefore, our main focus will be mainly on the features of waves allocated on the upper branch but considering some interesting nonlinear effects due to the fractional-order frame that occur on the lower branch.

**Figure 2:**
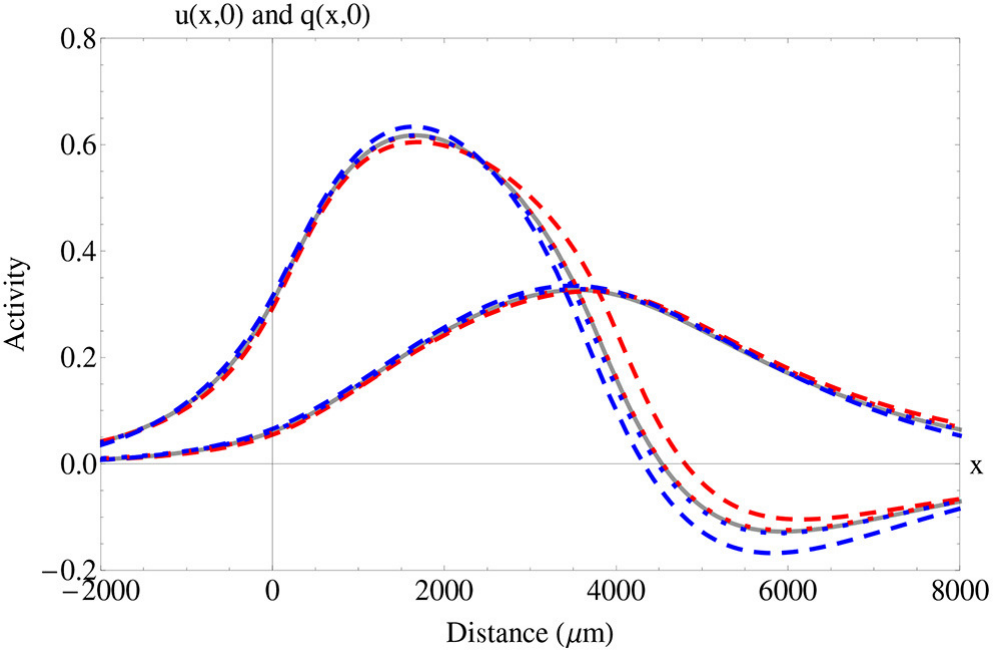
Fractional and integer-order traveling wave solutions *u*_⋆_(*x, t*), *q*_⋆_(*x, t*), *u*_⋆*L*_(*x, t*), *q*_⋆*L*_(*x, t*), *u*_⋆*R*_(*x, t*), and *q*_⋆*R*_(*x, t*). In this plot, we show the spatial wave profiles for a fixed initial time of *t* = 0. The solid gray lines correspond to the activity (leftmost wave) and adaptation (rightmost wave) in the integer-order case α = 1. The dotted red lines correspond to α = 0.99, the dashed red lines correspond to α = 0.9, the dotted blue lines correspond to α = 1.01, and the dashed blue lines correspond to α = 1.1. We consider two distinct traveling waves with similar features that are located in the upper (stable) branch to compare the effect of fractional-order on wave characteristics. For the integer-order α = 1, the wave speed is *c* = 402.8 μm/ms, the wave width is *w* = 3616.1 μm, and the synaptic threshold is *k* = 0.304. For the fractional-order α = 0.99, the wave speed is *c* = 405.1 μm/ms, the wave width is *w* = 3625.6 μm, and the synaptic threshold is *k* = 0.302. For the fractional-order α = 0.9, the wave speed is *c* = 433.8 μm/ms, the wave width is *w* = 3887.5 μm and the synaptic threshold is *k* = 0.292. For the fractional-order α = 1.01, the wave speed is *c* = 398.7μm/ms, the wave width is *w* = 3, 570 μm, and the synaptic threshold is *k* = 0.305. For the fractional-order α = 1.1, the wave speed is *c* = 379 μm/ms, the wave width is *w* = 3438.6 μm and the synaptic threshold *k* = 0.314. Parameters fixed for this plot: β = 1, ϵ = 0.1, and σ = 1, 000 μm.

In Figure 2, we consider wave profiles under different fractional derivative orders with similar wave features. We note that, in comparison to the integer-order case, the features of wave width and wave speed were increased for α ≈ 1^−^ whereas the synaptic threshold was diminished. On the other hand, the features of wave width and wave speed were decreased for α ≈ 1^+^, whereas synaptic threshold was increased. This analysis suggests that the memory effect does indeed affect the *initial* features of a wave under the same parameter choice. In particular, for values of α ≈ 1^+^, more synaptic input is required to produce a wave with diminished features, whereas for values of α ≈ 1^−^, less synaptic input is required to produce a fractional-order wave with increased features.

**Figure 3:**
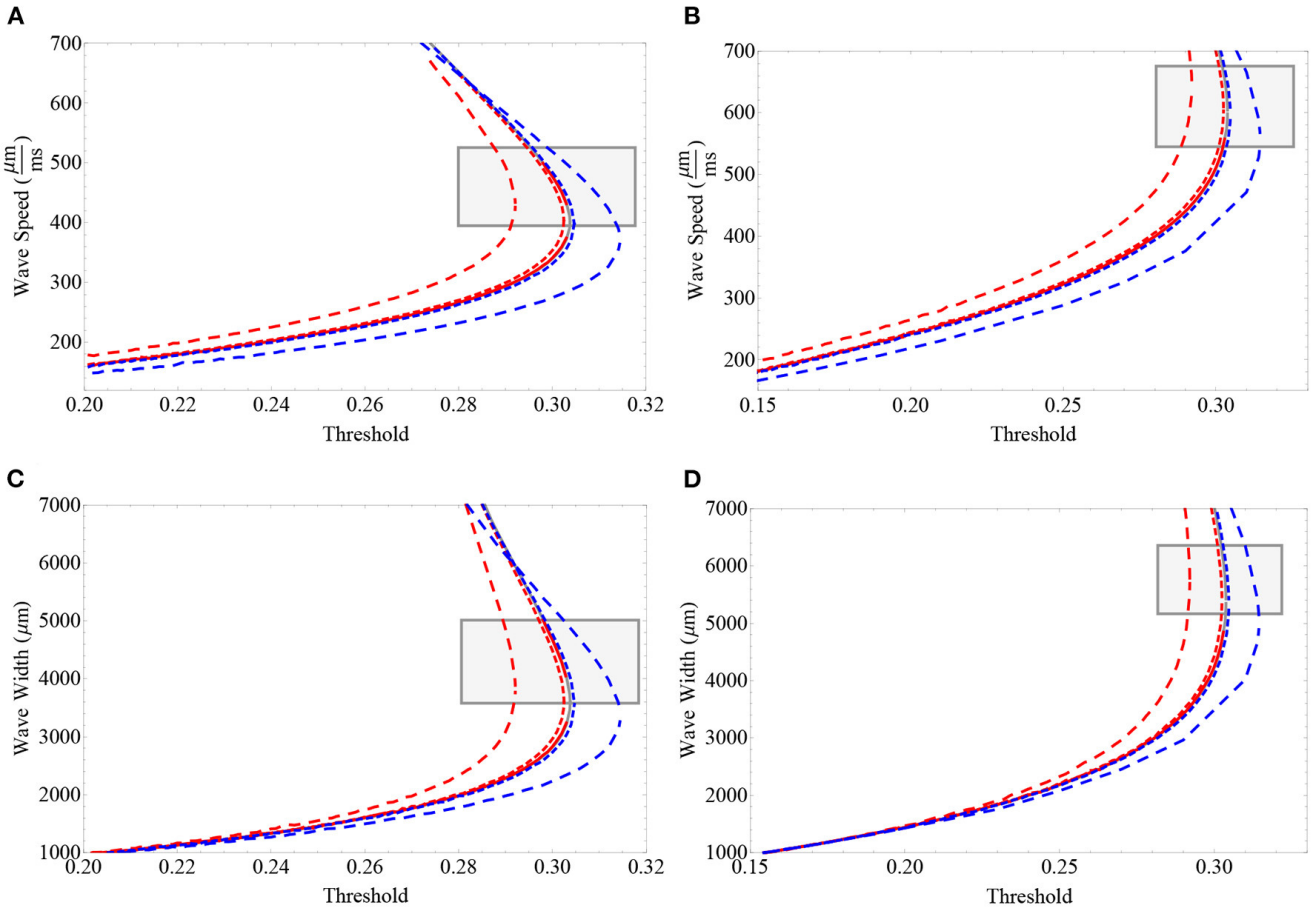
Wave speed vs. synaptic threshold **(A,B)** and wave width vs. synaptic threshold **(C,D)**. The values of the synaptic connectivity range include the following: for **(A,C)** σ = 1, 000 μm, and for **(B,D)** σ=1,500 μm. Gray lines correspond to the integer-order case α = 1, blue lines correspond to α ≈ 1^+^ (dashed lines α = 1.01, dotted lines α = 1.1), and red lines correspond to α ≈ 1^−^ (dashed lines α = 0.99, dotted lines α = 0.9). The gray rectangles determine the regions of interest near the upper (stable) branch that are suited for our explicit approximations considering the error estimates described in the Supplementary Material. We acknowledge that a big portion of the stable branch (not shown) cannot be analyzed by these approximations. In **(A,B)**, we note that the effect of the order of the derivative in wave speed is to modify the synaptic threshold in which a fixed speed is achieved. For values α ≈ 1^−^, a fixed wave speed is achieved with less synaptic threshold compared to values of α ≈ 1^+^. In **(C,D)**, we note that, in general, the effect of the order of the derivative in wave width is also to modify the synaptic threshold in which such a width is achieved. However, in this case, a nonlinear effect of the order of the derivative and extent of the synaptic connectivity on the lower branch is present. For values α ≈ 1^−^, a fixed wave width is achieved with less synaptic threshold compared to values of α ≈ 1^+^. Parameters fixed for these plots: β = 1 and ϵ = 0.1.

In Figure 3, we analyze the neuronal collective memory effect due to the fractional derivative order by considering wave speed vs. synaptic threshold and wave width vs. synaptic threshold curves. Our main observation is that in the upper branch, the memory effect directly affects both wave speed and wave width. Fractional derivative orders of α ≈ 1^−^ tend to diminish the synaptic threshold necessary to achieve a fixed speed and width. On the other hand, values of α ≈ 1^+^ tend to increase the synaptic threshold necessary for achieving a fixed speed and width. Projections of fractional-orders more distant from 1 exhibit a consistent effect of fractional-order on wave propagation speed. That is, in our analysis, memory effect *initially* increases wave speed and width (α ≈ 1^−^) or decreases wave speed and width (α ≈ 1^+^). The relationship between fractional-order and wave width is more complex on the lower branch of unstable waves and is observed to be also affected by the extent of the synaptic connectivity and the synaptic threshold. In particular, we note that the effect of fractional-order on wave speed seems to be different to the effect of fractional-order on wave width. Fractional-order modifies the feature of wave speed on both unstable and stable branch. However, fractional-order does not modify the feature of wave width on a portion of the unstable branch. That is, when considering sufficiently low synaptic thresholds, there is no major effect of fractional-order on wave width. The results in Figure 3 suggest that the effect of fractional-order derivative on wave width might be dependent on the synaptic-threshold.

**Figure 4:**
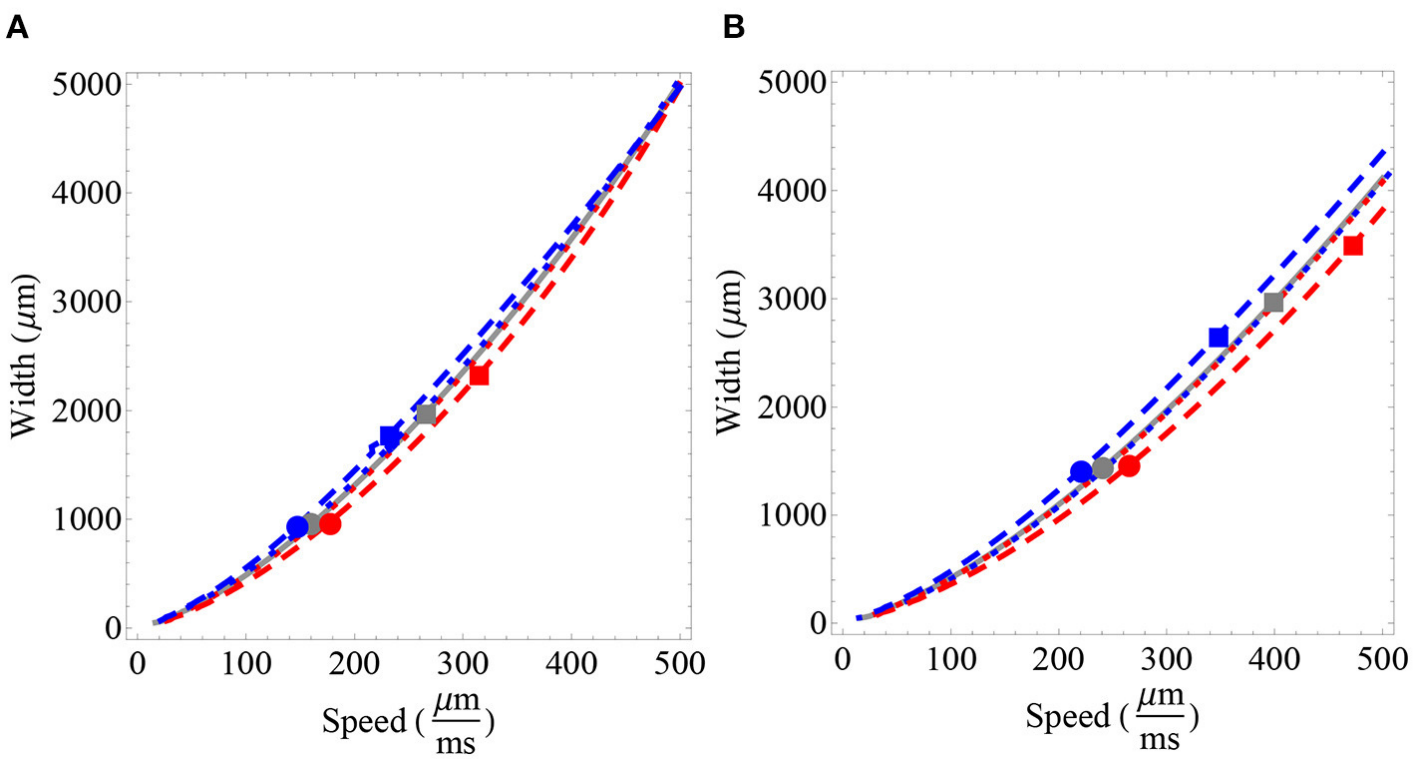
Wave width vs. wave speed for different fractional-order estimates and synaptic connectivity. Gray lines correspond to the integer-order case α = 1, blue lines correspond to α ≈ 1^+^ and red lines correspond to α ≈ 1^−^. Short dashes represent values of α closer to 1, that is, short red dashes represent α = 0.99 and short blue dashes represent α = 1.01. Large red dashes represent α = 0.9 and blue large dashes represent α = 1.1. The gray point, the red point and the blue point represent wave features for a fixed synaptic threshold of *k* = 0.2 for α = 1, α = 0.9, and α = 1.1, respectively. Similarly, the gray square, the red square, and the blue square represent the fixed synaptic threshold of *k* = 0.28. **(A)** We fix σ = 1, 000 μm. **(B)** We fix σ = 1, 500 μm. **(A,B)** We note that the relationship between wave width and fractional-order is not substantially affected for a wave with correspondent low synaptic threshold. We also observe that a fractional-order of α ≈ 1^−^ tends to increase wave width and wave speed relative to α = 1. On the other hand, α ≈ 1^+^ tends to decrease wave width and wave speed relative to α = 1. The previous analysis is valid for sufficiently wide waves, as observed in Figure 3. The wave features obtained for *k* = 0.2 (unstable branch) only modify wave speed. On the other hand, in considering *k* = 0.28 we observe an effect on both the width and the speed of the wave. This change is also affected by the synaptic connectivity range. Parameters fixed for all plots: β = 1 and ϵ = 0.1.

One of the advantages of the approximations developed in this section is that we can explore the initial effect of fractional-order on distinct parameter relations. In Figure 4, we analyze the relationship between wave width and wave speed for different fixed synaptic thresholds. We find a direct effect of fractional-order on wave speed and a nonlinear effect on wave width, consistent with the analysis developed in Figure 3. For a relatively small synaptic threshold (determining wave solutions lying in the lower branch), we find a slight increase in wave speed (α ≈ 1^−^) and a slight decrease in wave speed (α ≈ 1^+^), with a nearly insignificant change in width. For a larger synaptic threshold (determining wave solutions in the upper branch), we find results consistent with Figures 2, 3: an increase in wave speed and wave width (α ≈ 1^−^) and a decrease in wave speed and wave width (α ≈ 1^+^) (see Figure 4 for details).

Following the analysis developed previously, we suggest that the memory effect due to the fractional-order derivative plays a role in the properties of traveling wave solutions of fractional neural field models. We hypothesize as follows: in the case of α ≈ 1^−^, the memory of the system, the history of neural activity elapsed over time, *initially* increases wave speed and wave width. On the other hand, in the case of α ≈ 1^+^, we note a decrease in the wave speed and wave width. We also note a plausible synaptically-dependent effect of fractional-order on wave width. For considerably low synaptic thresholds (on the unstable branch) there appears to be no impact of fractional-order on the feature of wave width. Therefore, we hypothesize a nonlinear effect of fractional-order on the feature of wave width. We claim that this initial dynamics are of interest, as they might provide information about transient dynamics during the creation of propagating activity. Our previous results are very restricted since they are only applicable to values of α ≈ 1, under specific conditions. Therefore, in the next section we provide further work to support our observations.

### 3.5. Adomian Decomposition Method

In this section, we utilize the Adomian decomposition method to approximate fractional traveling wave solutions in a wider range of fractional-orders and longer times. Adomian decomposition has been successfully applied to obtain asymptotic expansions of traveling wave solutions in the Korteweg-de Vries (KdV) equation, Burgers' equation, and wave equation, among others (Wazwaz, 2001; Jafari and Daftardar-Gejji, 2006; Wang, 2006; Abbasbandy, 2007). Its convergence and recursive formulas were established in Adomian (1988), Cherruault (1990), Abbaoui and Cherruault (1995), and Wazwaz (2000).

Some limitations of this method were reported in Abbasbandy (2007), in the results obtained from this approximation for solving a generalized coupled KdV equation were revealed to be valid only for small values of *x* and *t*. However, in Adomian (1988), Adomian (1994), Wazwaz (1997), and Wazwaz (2001), it is shown that the capability of the Adomian decomposition method can be directly improved by determining further terms in the approximation. In the Supplementary Material, we establish absolute error estimates of the Adomian Decomposition Method considering a first-order initial condition. The error estimates depend on different features, such as synaptic-threshold (and therefore wave width and wave speed). Based on these error estimates we limit the values of *t* to be analyzed.

There are advantages and disadvantages using the Adomian decomposition method in comparison to the Mittag-Leffler extensions developed in section 3. An advantage of this method is that a more general kernel can be used in the spatial synaptic connectivity term, longer times and different synaptic connectivity extents can also be analyzed. A disadvantage is that it is numerically challenging to obtain the relationship among fractional-order, the different model parameters and wave features (e.g., the analysis performed in Figures 3, 4). We claim that both approaches can provide a complementary insight on the effect of fractional-order on cortical wave features.

We consider again a fractional-order neural fiel model:


(33) $$\begin{array}{l} \begin{array}{c} D_{t}^{\alpha }u\left(x,t\right)=-u\left(x,t\right)+\int_{-\infty }^{\infty }g\left(x-y\right)H\left(u\left(y,t\right)-k\right)dy-\beta q\left(x,t\right)\\ D_{t}^{\alpha }q\left(x,t\right)=\epsilon u\left(x,t\right)-\epsilon q\left(x,t\right). \end{array} \end{array}$$

In this new approach, the kernel choice *g*(*x*) can be a more general symmetric monotonically decreasing function, as long as it is sufficiently smooth. For our analysis, we consider a gaussian kernel, $g\left(x\right)\;=\;\frac{1}{\sigma \sqrt{2\pi }}e^{\frac{-x^{2}}{2\sigma^{2}}}$.

### 3.6. Approximate Traveling Wave Solution for 0 < α <1

We consider a fractional neural field model (System 33) for 0 < α <1. This method consists of considering the first-order traveling wave solutions (Equations 2,3) as initial conditions. That is:


(34) $$\begin{array}{l} u\left(x,0\right)=u_{⋆}\left(x,0\right), \end{array}$$

and


(35) $$\begin{array}{l} q\left(x,0\right)=q_{⋆}\left(x,0\right). \end{array}$$

Applying the Adomian decomposition method we obtain approximate traveling wave solutions. In Section 5 of the Supplementary Material we provide details regarding the procedure to obtain such traveling wave solutions. Using a 4α approximation to increase the capability of the method, we obtain the following traveling wave solution for the activity variable:


(36) $$\begin{array}{l} \begin{array}{cc} u_{f}\left(x,t\right) & \approx u_{⋆}\left(x,0\right)+f_{1}\left(x\right)\frac{t^{\alpha }}{\Gamma \left(\alpha +1\right)}+f_{2}\left(x\right)\frac{t^{2\alpha }}{\Gamma \left(2\alpha +1\right)}\\ +f_{3}\left(x\right)\frac{t^{3\alpha }}{\Gamma \left(3\alpha +1\right)}+f_{4}\left(x\right)\frac{t^{4\alpha }}{\Gamma \left(4\alpha +1\right)}, \end{array} \end{array}$$

and for the adaptation variable:


(37) $$\begin{array}{l} \begin{array}{cc} q_{f}\left(x,t\right) & \approx q_{⋆}\left(x,0\right)+h_{1}\left(x\right)\frac{t^{\alpha }}{\Gamma \left(\alpha +1\right)}+h_{2}\left(x\right)\frac{t^{2\alpha }}{\Gamma \left(2\alpha +1\right)}\\ +h_{3}\left(x\right)\frac{t^{3\alpha }}{\Gamma \left(3\alpha +1\right)}+h_{4}\left(x\right)\frac{t^{4\alpha }}{\Gamma \left(4\alpha +1\right)}. \end{array} \end{array}$$

The description of each of the terms of the previous expressions, as well as the details of the Adomian decomposition method, are contained in Section 5 of the Supplementary Material. In the Supplementary Material, we also provide error estimates of the Adomian approximated solution.

**Figure 5:**
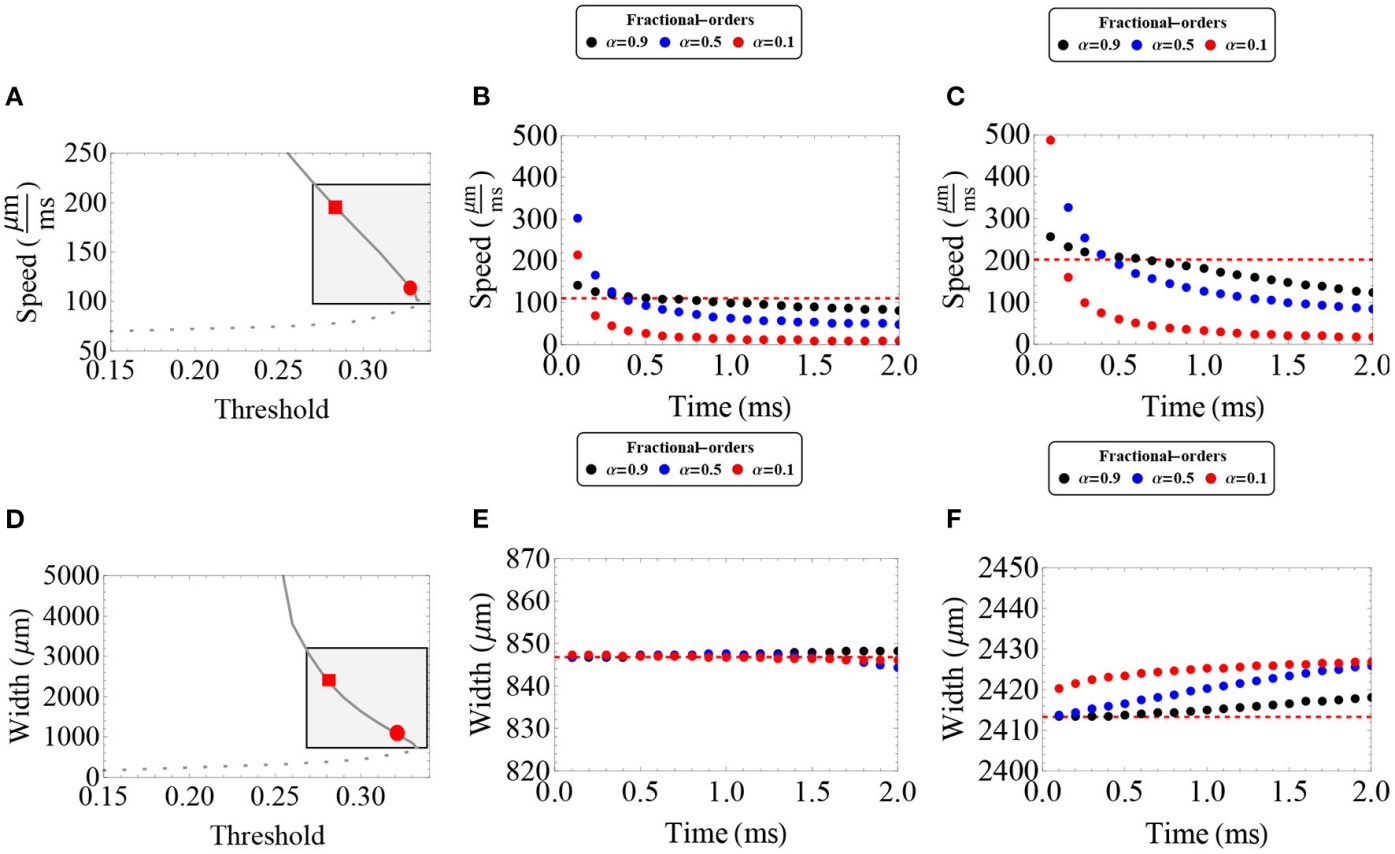
Wave speed and wave width as a function of time as estimated by the Adomian Decomposition Method in the case of 0 < α <1. **(A,D)** Wave speed vs. synaptic threshold and wave width vs. synaptic threshold, respectively. The gray rectangles determine the regions of interest in the upper (stable) branch that are suited for the Adomian Decomposition Method according to the error estimates established in the Supplementary Material (up to *t* = 2 ms). Here, we choose two distinct wave solutions to analyze the effect of fractional-order on wave features. The “red square solution” determines a wave solution considering *k* = 0.28 (*c* = 202 μ*m*/*ms* and *w* = 2, 413 μm) and the “red circle solution” determines a wave solution considering *k* = 0.33 (*c* = 110 μ m/ms and *w* = 847 μm). **(B,C,E,F)** Wave speed and wave width for the wave solution as time evolves determined by the red circle solution **(B,E)** and red square solution **(C,F)**, respectively. The different color dots determine distinct fractional-orders. The red dashed lines determine the features of the integer-order initial solution. **(B,C)** The fractional wave solutions present an initial increase in wave speed, in agreement with the Mittag-Leffler approximations, followed by a subsequent and significant decrease in wave speed. **(E)** The fractional wave solutions α ≈ 1 present an insignificant increase in wave width. **(F)** The fractional wave solutions present a slight increase in wave width. **(A–F**) Parameters fixed: ϵ = 0.1, β = 1.0, and σ = 300 μm.

In Figure 5, we analyze the evolution of two different waves lying in the upper branch of stable waves using the Adomian approximation. Due to the characteristics of this method we can now consider a shorter synaptic connectivity extent (σ = 300 μm), and waves lying in an upper portion of the stable branch. However, a similar analysis has been made for longer synaptic connectivity extents (e.g., σ = 1, 000 μm), obtaining qualitatively similar results. In Figures 5B,C,E,F, we show the evolution of the wave speed (at the front of the wave) and the wave width according to time intervals suggested by our error estimates. In both cases, we find an initial increase in wave speed, consistent with our results from the Mittag-Leffler approximations, followed by a subsequent decrease in wave speed. In the initial increase of speed we find that, in general, lower orders imply faster speeds. After that, the wave speed was dramatically reduced with lower order implying slower waves. The effect of fractional-order on wave width was more complex. For relatively low synaptic threshold (*k* = 0.28), we find that the wave width was slightly increased, whereas for higher synaptic threshold (*k* = 0.33) the increase of the wave width was minimum. In the first case, a lower fractional-order imply more increase in the feature of wave width. With the Mittag-Leffler approximations we were not able to analyze waves in the stable branch with lower synaptic thresholds. However, the results of this approach are consistent with the intuition gained from the Mittag-Leffler approximations: the effect of fractional-order on wave width is determined by the synaptic threshold.

**Figure 6:**
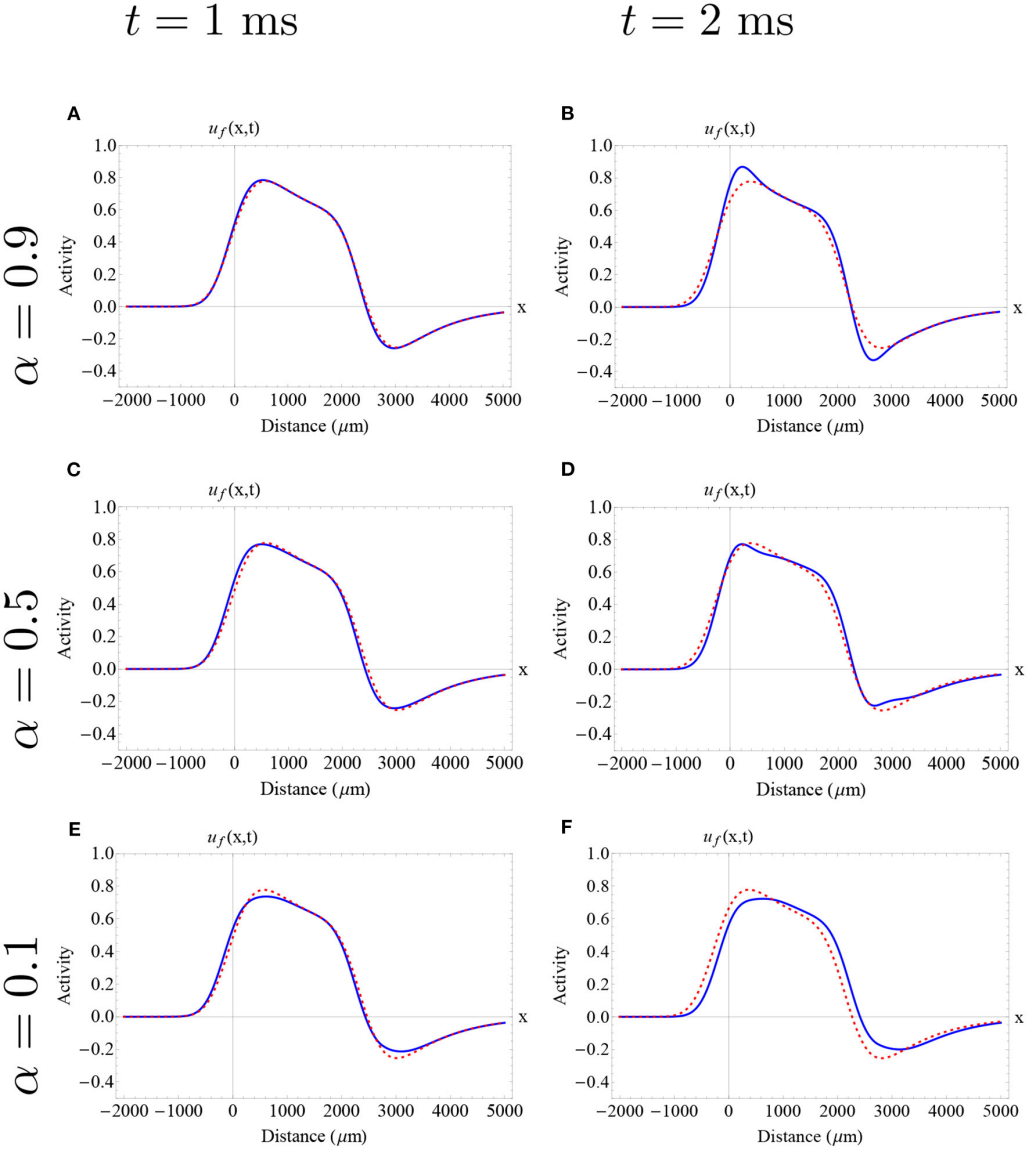
Approximate fractional-order traveling pulse solutions using the Adomian decomposition method in the case of 0 < α <1. The dashed red curve denotes the integer-order initial pulse solution (Equation 5) determined by the “red square solution” and the blue curve denotes the approximate fractional pulse solution. Each row and column determine a different fractional-order and time as is described in the caption. **(A–F)** The effect of fractional-order on wave speed is nonlinear. In all cases, there is an initial increase of wave speed, followed by a decrease in wave speed. On the other hand, for all fractional orders and all times, we find a consistent and slight increase in wave width as is described in Figure 5. The fractional-order approximations provide an insight of a possible effect of fractional-order on wave profile finding a slight change in the wave amplitude. **(A–F)** Parameters fixed: ϵ = 0.1, β = 1.0, *k* = 0.33 and σ = 300 μm.

**Figure 7:**
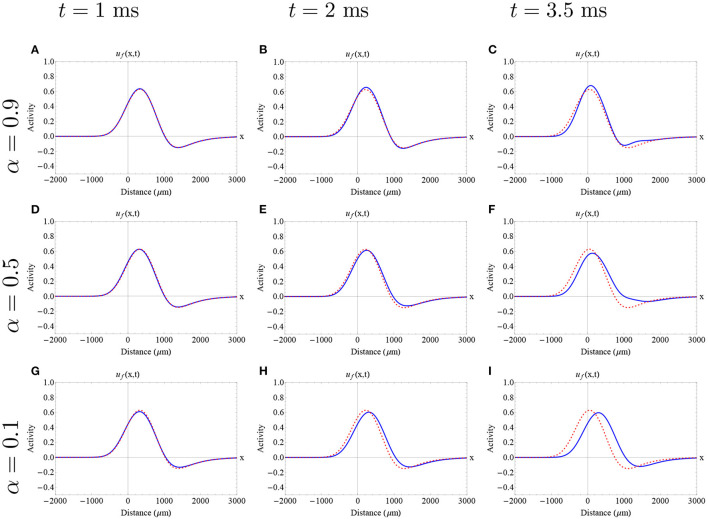
Approximate fractional-order traveling pulse solutions using the Adomian decomposition method in the case of 0 < α <1. The dashed red curve denotes the integer-order initial pulse solution (Equation 5) determined by the “red circle solution” and the blue curve denotes the approximate fractional pulse solution. Each row and column determine a different fractional-order and time as is described in the caption. **(A–I)** The effect of fractional-order on wave speed is nonlinear. In all cases, there is an initial increase of wave speed, followed by a decrease in wave speed. The fractional-order approximations provide an insight of a possible effect of fractional-order on wave profile finding a slight increase in the wave amplitude. **(A–I)** Parameters fixed: ϵ = 0.1, β = 1.0, *k* = 0.28 and σ = 300 μm.

In Figures 6, 7, we show the initial profile of the two previously analyzed traveling wave solution, presented as initial conditions in , with Figure 5, with different fractional-orders utilizing the Adomian decomposition method for 0 < α <1. Here, we observe initial differences on the wave profile due to their position in the stable branch (narrower wave and wider wave). For all the fractional-orders analyzed here, we obtained an initial increase in wave speed, followed by a decrease in wave speed at later times as is described in Figure 5. The time interval chosen for each wave is based in its correspondent error estimate. In both cases, a slight change of profile can be observed in this short time interval. In particular, for high fractional-orders a small change in the wave amplitude is observed. Due to the nature of our methods, we cannot detect the exact effect of fractional-order on wave shape. Thus, a further analysis of the effect of fractional-order on wave shape needs to be addressed in the future.

**Figure 8:**
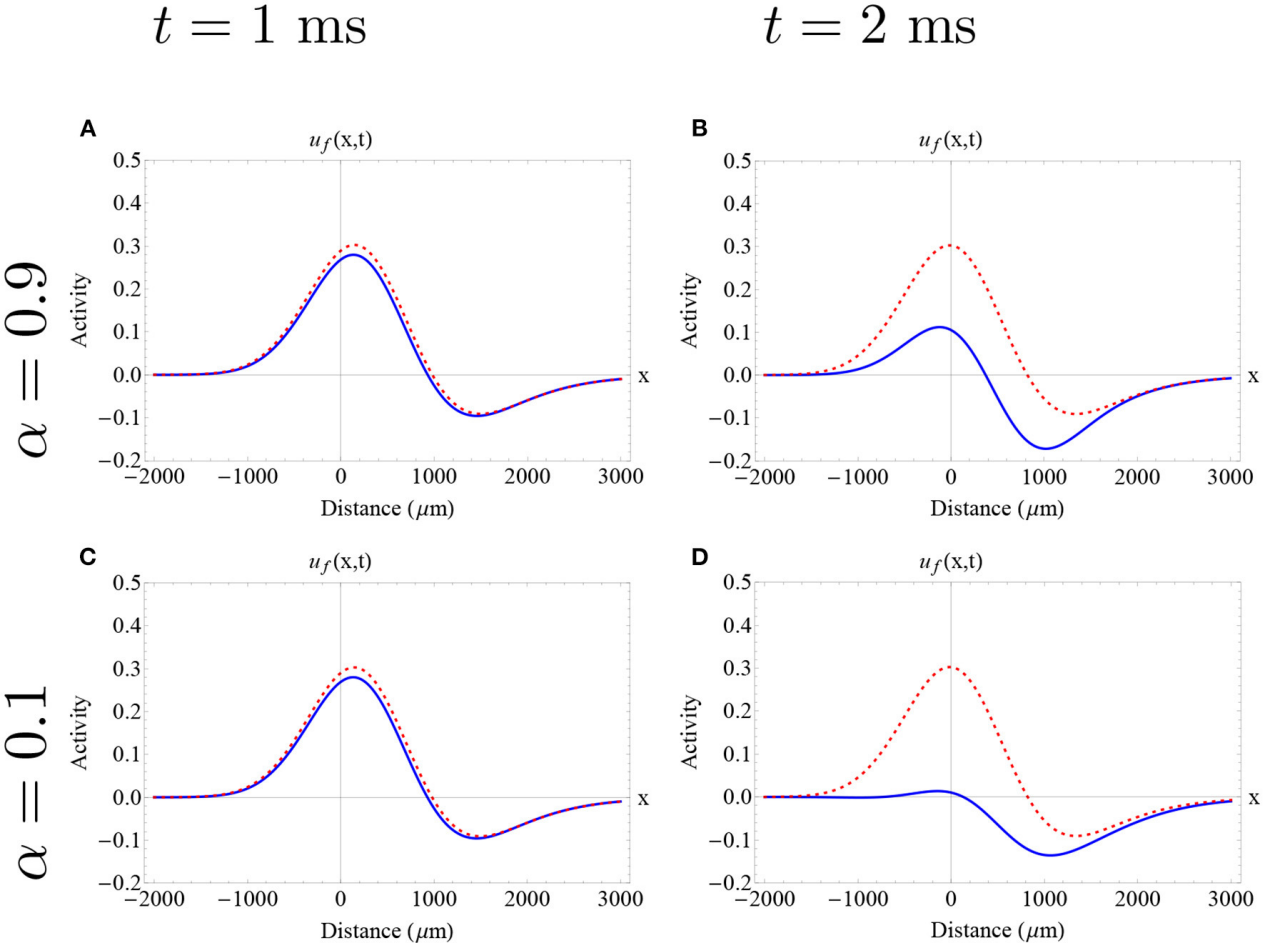
Approximate fractional-order traveling pulse solutions with different fractional orders using the Adomian decomposition method on the unstable branch considering the case of 0 < α <1. The dashed red curve denotes the explicit integer-order pulse solution (Equation 5). The dashed green line denotes the synaptic threshold, the blue curve denotes the fractional pulse, and the red dots determine the points at which the synaptic threshold is achieved. Each row and column determines a different fractional order and time. **(A)** Fractional-order α = 0.9 at time *t* = 1, we note a slight decrease in wave speed and slight decrease in wave width. **(B)** Fractional-order α = 0.9 at *t* = 2, the fractional pulse solution is no longer above the synaptic threshold, hence it is no longer considered a pulse solution. **(C,D)** Fractional-order α = 0.1 at time *t* = 1 and time *t* = 2, respectively. Similarly to **(C)**, the fractional pulse solution is no longer above the synaptic threshold. **(A–D)** Parameters fixed: ϵ = 0.1, β = 1.5, *k* = 0.25 and σ = 500 μm. Initial wave features *c* = 160 μm/ms and *w* = 589 μm.

In Figure 8, we analyze approximate fractional-order wave solutions on a wave located on the unstable branch. Our aim is to show the different effect of the method on the stable and the unstable branch. For the different fractional-orders analyzed here the pulse disrupted below the synaptic threshold, and was no longer considered a pulse solution.

In summary, the results of the case of 0 < α <1 show that the effect of fractional order is to initially increase the wave speed, and then significantly decreasing it. The initial increase is supported by the results obtained from the Mittag-Leffler approximations. This implies that the modeling of cortical *in vivo* wave propagation using fractional-order neural field models is severely affected by the fractional-order choice. Also, the effect of fractional-order on wave width is nonlinear and determined by the synaptic threshold and synaptic connectivity extent.

### 3.7. Traveling Wave Solution for 1 < α < 2

We consider the fractional neural field model (System 33) for 1 < α < 2, but now under the following initial conditions:


(38) $$\begin{array}{l} u\left(x,0\right)=u_{⋆}\left(x,0\right), \end{array}$$

(39) $$\begin{array}{l} u_{t}\left(x,0\right)=\frac{\partial u_{⋆}}{\partial t}\left(x,0\right), \end{array}$$

(40) $$\begin{array}{l} q\left(x,0\right)=q_{⋆}\left(x,0\right), \end{array}$$

and


(41) $$\begin{array}{l} q_{t}\left(x,0\right)=\frac{\partial q_{⋆}}{\partial t}\left(x,0\right). \end{array}$$

Applying the Adomian decomposition method, we obtain the approximated wave solutions. For details regarding the Adomian decomposition method, please see Section 5 of the Supplementary Material. The approximate wave solutions employing a 4α approximation are the following:


(42) $$\begin{array}{l} \begin{array}{cc} u_{f}\left(x,t\right) & \approx u_{⋆}\left(x,0\right)+\frac{\partial u_{⋆}}{\partial t}\left(x,0\right)+f_{1}\left(x\right)\frac{t^{\alpha }}{\Gamma \left(\alpha +1\right)}\\ +f_{2}\left(x\right)\frac{t^{2\alpha }}{\Gamma \left(2\alpha +1\right)}+f_{3}\left(x\right)\frac{t^{3\alpha }}{\Gamma \left(3\alpha +1\right)}\\ +f_{4}\left(x\right)\frac{t^{4\alpha }}{\Gamma \left(4\alpha +1\right)}, \end{array} \end{array}$$

and


(43) $$\begin{array}{l} \begin{array}{cc} q_{f}\left(x,t\right) & \approx q_{⋆}\left(x,0\right)+\frac{\partial q_{⋆}}{\partial t}\left(x,0\right)+h_{1}\left(x\right)\frac{t^{\alpha }}{\Gamma \left(\alpha +1\right)}\\ +h_{2}\left(x\right)\frac{t^{2\alpha }}{\Gamma \left(2\alpha +1\right)}+h_{3}\left(x\right)\frac{t^{3\alpha }}{\Gamma \left(3\alpha +1\right)}\\ +h_{4}\left(x\right)\frac{t^{4\alpha }}{\Gamma \left(4\alpha +1\right)}. \end{array} \end{array}$$

The description of each of the terms of the previous expressions as well as the details regarding the Adomian decomposition method are established in Section 5 of the Supplementary Material.

**Figure 9:**
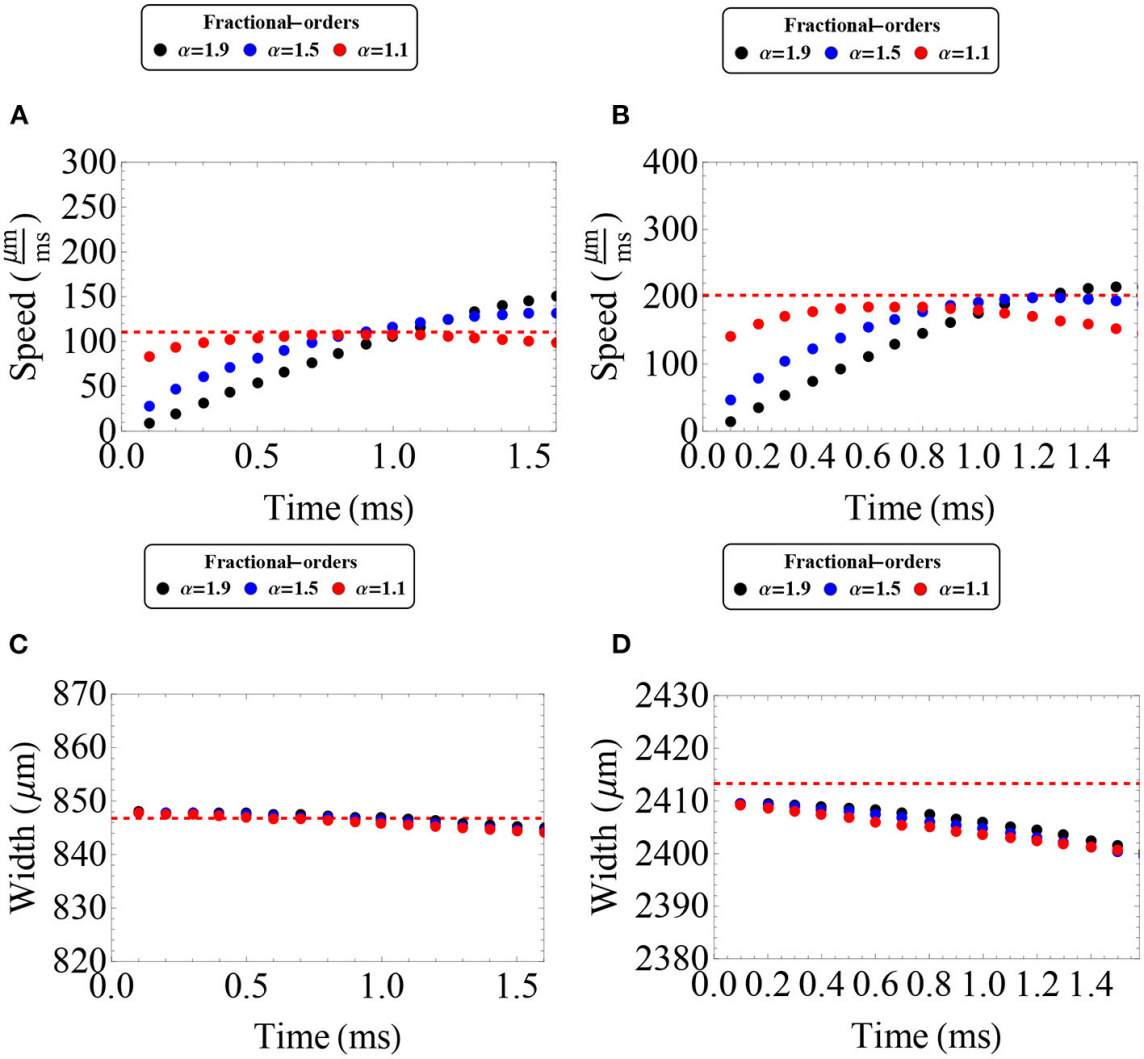
Wave speed and wave width as a function of time as estimated by the Adomian Decomposition Method in the case of 1 < α < 2. **(A–D)** Wave speed and wave width for the wave solution determined by the “red circle solution” **(A,C)** and “red square solution” **(B,D)**, respectively. The different color dots determine distinct fractional-orders. The red dashed lines determine the features of the integer-order solutions initial solution. We analyze up to *t* = 1.5 in correspondence to the error estimates established in the Supplementary Material. **(A,B)** The fractional wave solutions present an initial decrease in wave speed, in agreement with the Mittag-Leffler approximations, followed by a subsequent increase in wave speed. **(C)** The fractional wave solutions present an insignificant decrease in wave width. **(D)** The fractional wave solutions present a slight decrease in wave width. A similar analysis has been made for longer synaptic connectivity ranges obtaining qualitatively similar results. **(A–D)** Parameters fixed: ϵ = 0.1, β = 1.0, and σ = 300 μm.

In Figure 9, we analyze the evolution of the two different waves lying in the upper branch of stable waves, described in Figure 5, using the Adomian approximation in the case of 1 < α < 2. We show the evolution of the wave speed (at the front of the wave) and the wave width according to time intervals suggested by our error estimates. In both cases, we find an initial decrease in wave speed, consistent with our results from the Mittag-Leffler approximations, followed by a subsequent increase in wave speed. In the initial decrease of speed we find that, in general, lower orders imply less decrease. After that, the wave speed was increased. For relatively low synaptic threshold (*k* = 0.28), we find that the wave width was slightly decreased, whereas for higher synaptic threshold (*k* = 0.33), the decrease of the wave width was minimum. In the first case, a lower fractional-order imply more decrease in the feature of wave width. This result is also consistent with the results from the Mittag-Leffler approximations: the effect of fractional-order on wave width is determined by the synaptic threshold.

**Figure 10:**
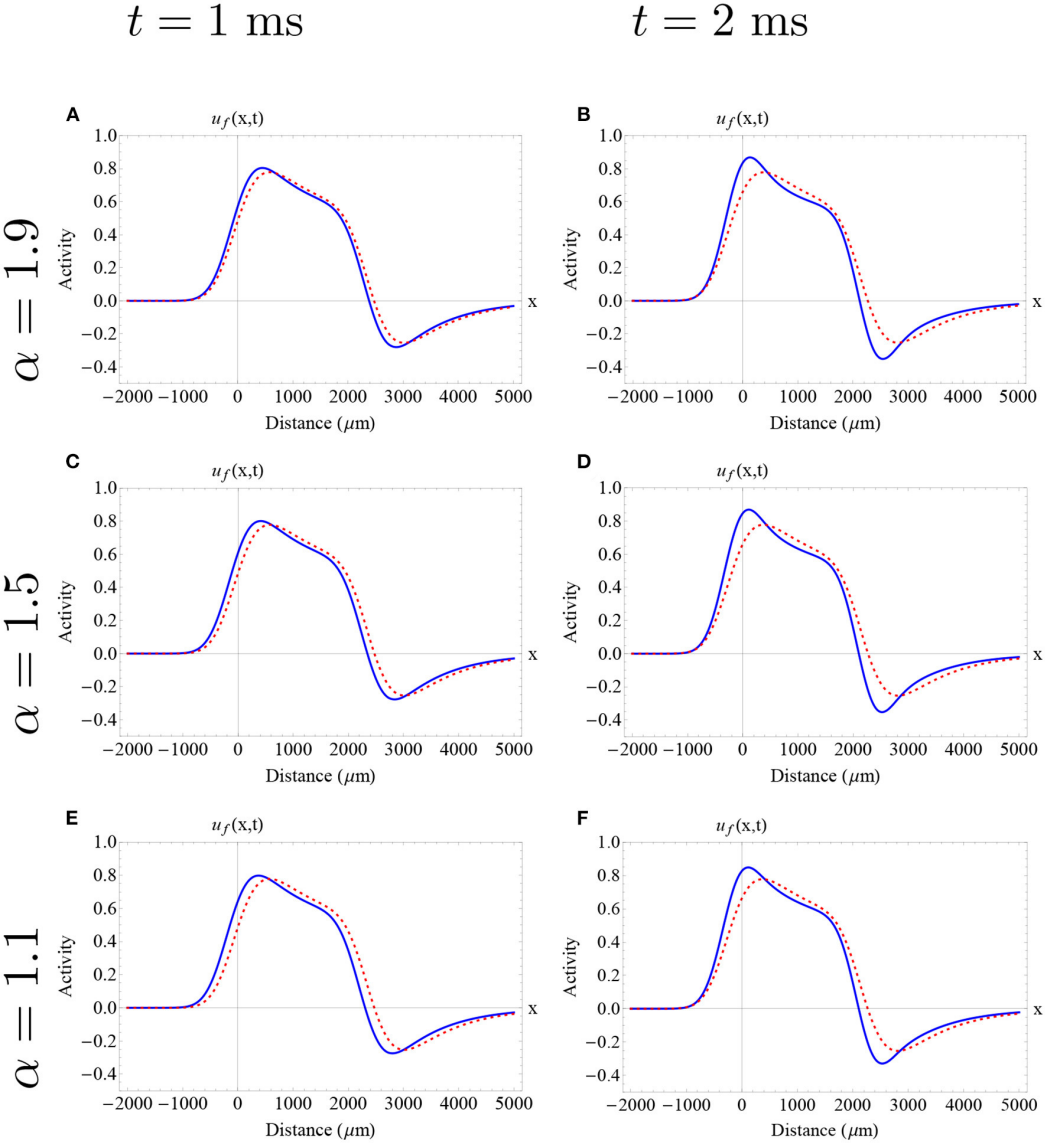
Approximate fractional-order traveling pulse solutions using the Adomian decomposition method in the case of 1 < α < 2. The dashed red curve denotes the integer-order initial pulse solution (Equation 5) determined by the “red square solution” and the blue curve denotes the approximate fractional pulse solution. Each row and column determine a different fractional-order and time as is described in the caption. **(A–F)** The effect of fractional-order on wave speed is nonlinear. In all cases, there is an initial decrease of wave speed, followed by an increase in wave speed. On the other hand, for all fractional orders and all times, we find a consistent and slight decrease in wave width as is described in Figure 9. The fractional-order approximations provide an insight of a possible effect of fractional-order on wave profile. **(A–F)** Parameters fixed: ϵ = 0.1, β = 1.0, *k* = 0.28 and σ = 300 μm.

In Figure 10, we depict an example of a fractional-order approximate solutions considering the initial conditions previously discussed, but now in the case of 1 < α < 2. Here, we find that all fractional-orders exhibited an initial decrease in wave speed and width consistent with the analysis developed in section 3. For all fractional-orders and low times, we find a consistent and very slight (less than 150 μm) decrease in wave width. It is not possible to establish a qualitatively difference in the wave shape with this approach. Further work needs to be addressed to establish the effect of fractional-order on wave profile.

The analysis developed by using the Adomian decomposition method strengths our hypothesis established in Section 3. Regardless of the fractional-order considered, there is an effect of fractional-order on wave speed and wave width. Low fractional-orders (0 < α <1), tend to produce a slight increase in wave width and similar shape to the integer-order case, at a cost of initially increasing wave speed and significantly decreasing the wave speed at later times. On the other hand, the initial effect of fractional-orders (1 < α < 2) is to decrease wave width and speed. After this transient effect on the feature of wave speed, the wave speed tends to increase. The limitations of the approximation does not permit to know the exact evolution later in time of this case but the reduction on wave width is an important effect due to the fractional-order. Therefore, the possible memory repercussion due to a fractional-order approach exerts a significant effect on wave features modeled by neural fields.

## 4. Discussion

In this work, we established a novel study regarding the existence of approximated fractional-order traveling wave solutions to describe wave features observed in *in vivo* clinical recordings. We focused our efforts on two different ranges of fractional orders: 0 < α <1 and 1 < α < 2. In our work, the characteristics shown in the wave solutions were considerably different in each of these cases. First, our Mittag-Leffler approximations provided information of plausible *initial* fractional-order dynamics when considering the change from a first-order to a fractional-order framework. In the case of α ≈ 1^−^, our approximations converged to the first-order case as α → 1^−^. They provided evidence of an initial and transient activity increase. On the other hand, in the case of α ≈ 1^+^, the estimates did not converge to the first-order case as α → 1^+^. The usefulness of the latter case was restricted when considering long synaptic connectivity extents and low speeds. This is one of the limitations of our approximations. For such scenarios, we found an initial activity decrease. We complemented our analysis with explicit Mittag-Leffler error estimates, described in the Supplementary Material, that motivated the use of such approximations.

Second, the implementation of the Adomian decomposition method provided information regarding a wider range of fractional orders covering 0 < α < 2. Since the effectiveness of this method relies on the order expansion choice, we limited our analysis to convenient time intervals motivated by the error estimates herein established. Using this approach, we recovered the initial transient dynamics previously established by the Mittag-Leffler approximations and observed further dynamics changes. In particular, in the case of 0 < α <1, after the initial effect captured by the Mittag-Leffler approximation, a decrease of activity was observed. On the other hand, considering the case of 1 < α < 2, we observed an activity increase after an initial decrease of activity. Therefore, both of our solutions agreed on the initial transient effects, and the Adomian decomposition method provided evidence of distinct dynamics as time increases. We also found evidence of an apparent synaptic-dependent fractional-order derivative effect using this methodology. In particular, wave solutions determined by higher synaptic thresholds had diminished feature change than those determined by lower synaptic thresholds, in which more acute changes were observed. Thus, the fractional-order derivative's memory effect might also depend on the synaptic activity threshold.

Since the fractional-order traveling wave approximated solutions have as free parameters: the wave speed (*c*), the wave width (*w*), and the synaptic connectivity extent (σ), the effect of fractional-order on solutions can only be analyzed by considering the matching conditions determined by Equation (7). The matching conditions provided the existence of traveling waves in the first-order case and the fractional-order case. Due to the number of free parameters, our work was designed to extract information about the relationship between the wave speed and the wave width, as these two features can be related to clinical data. Some of our study limitations are the use of convenient kernels in the Mittag-Leffler approach and limited synaptic connectivity extents for each of the approximations. To the authors' knowledge, this is the first study of fractional-order neural field models and provides a basis for future research considering the modeling of neuronal population activity under a fractional-order framework.

## 5. Conclusions

We established an initial study of traveling wave solutions of fractional-order neural field models in this work. We provided evidence of distinct effects on wave features considering the fractional temporal order as developed using the Caputo mathematical framework and a first-order wave solution as the initial condition. We hypothesized that the difference in characteristics is due to the neuronal collective memory effect of the fractional derivative. We found that for values of 0 < α <1, the memory tends to increase initially and then decrease the wave speed, while in the case of 1 < α < 2, the memory tends to decrease initially and then increase the wave speed. Also, our results showed that the effect of fractional-order on wave width is dependent on the synaptic threshold and the synaptic connectivity extent. Therefore, our results provided insight into how the memory effect due to the fractional-order derivative plays a complex role in studying wave patterns in neural fields. There are several advantages of considering a fractional-order scenario in comparison to a traditional integer-order framework. First, the model motivation extends naturally to a fractional-order scenario, and we can recover the first-order case when considering the limit α → 1^−^. In this model motivation, the fractional-order can account for different synaptic processes and scales of action. The fractional-order approach provides richer dynamics, in which the plausible memory index exerts different effects on the wave features. By considering a fractional-order approach, the problem's difficulty increases; however, it is possible to include more realistic modeling features similar to the expected non-linear nature of neuronal systems.

Future research directions include developing numerical and computational methods to implement the Caputo fractional-order derivative better and to analyze wave propagation features without restricting synaptic connectivity extents. In general, it is also of interest to understand the effect of fractional-order on different spatio-temporal patterns of activity. Also, it is desirable to investigate the effect of fractional-order on wave propagation by considering different fractional-order derivative definitions, and developing hypotheses of the plausible memory effect due to the fractional-order derivative definition choice.

## Data Availability Statement

The original contributions presented in the study are included in the article/Supplementary Material, further inquiries can be directed to the corresponding author.

## Author Contributions

LG-R designed the research, established the mathematical models, performed the mathematical analysis, performed the numerical simulations, and wrote the manuscript.

## Funding

This research was funded by SIP-IPN 2021-1285 and 2022-1416.

## Conflict of Interest

The author declares that the research was conducted in the absence of any commercial or financial relationships that could be construed as a potential conflict of interest.

## Publisher's Note

All claims expressed in this article are solely those of the authors and do not necessarily represent those of their affiliated organizations, or those of the publisher, the editors and the reviewers. Any product that may be evaluated in this article, or claim that may be made by its manufacturer, is not guaranteed or endorsed by the publisher.
