# Supplementary material for: Fractional-Order Traveling Wave Approximations for a Fractional-Order Neural Field Model
Source: Front Comput Neurosci. 2022 Mar 24;16:788924. doi: 10.3389/fncom.2022.788924 (PMC8987931; doi:10.3389/fncom.2022.788924)
Supplement: Supplementary file 1 [file Presentation_1.PDF]

Supplementary Material: Fractional-order traveling wave approximations for a fractional-order neural field model

1 FRACTIONAL CALCULUS DEFINITIONS

In this section, we establish some basic definitions and results from fractional-order derivatives with regard to Caputo and discuss its memory interpretation. For details and proofs of these properties, see (Podlubny, 1999; Ross, 1974; Ishteva, 2005; Ortigueira, 2011; Baleanu et al., 2016).

Define $\mathcal{C}$ as the class of piecewise continuous functions that are integrable on any subinterval of (a, ∞). Let f be on $\mathcal{C}$, $\alpha \in \mathbb{R}^+$, let $\Gamma(z) = \int_0^\infty t^{z-1}e^{-t}dt$ be the Gamma function (for properties of the Gamma function, see (Podlubny, 1999; Ross, 1974)). The *fractional integral operator of order* α is defined as:

$$ {}_aJ_t^\alpha f(t) = \frac{1}{\Gamma(\alpha)} \int_a^t \frac{f(\tau)}{(t-\tau)^{1-\alpha}} d\tau \tag{S1} $$

We remark that this integral operator is defined by fixing a lower limit a. To complement the previous definition we establish ${}_aJ_t^0 = I$. That is, the zeroth-order integral operator is the identity operator ${}_aJ_t^0 f(t) = f(t)$.

The fractional integral operator holds:

$$ {}_aJ_t^\beta \, {}_aJ_t^\alpha f(t) = {}_aJ_t^{\beta+\alpha} f(t), \beta \geq 0, \alpha \geq 0, \tag{S2} $$

and

$$ {}_aJ_t^\beta (t-a)^\mu = \frac{\Gamma(\mu+1)}{\Gamma(\mu+\beta+1)}(t-a)^{\mu+\beta}, \beta \geq 0, \mu > -1. \tag{S3} $$

In (Magin, 2004), a memory interpretation of a fractional-order integral operator of order α is established : $\alpha = 0$ represents zero memory, $0 < \alpha < 1$ represents intermediate memory, and $\alpha = 1$ represents complete memory. This interpretation is obtained from the specific kernel used in (S1).

Let $n \in \mathbb{Z}^+$ and let f be a function of class $\mathcal{C}$. Let $\alpha \in \mathbb{R}^+$ such that $n - 1 < \alpha \leq n$. Suppose f is of class $\mathcal{C}^n$. The *Caputo's fractional-order derivative of f of order* α is defined (Podlubny, 1999; Ross, 1974) as:

$$ {}_aD_t^\alpha f(t) := {}_aJ_t^{n-\alpha}(f^n(t)), \tag{S4} $$

where $f^n(t)$ is the nth order integer derivative of $f(t)$. That is,

$$ {}_aD_t^\alpha f(t) = \frac{1}{\Gamma(n-\alpha)} \int_a^t \frac{f^n(\tau)}{(t-\tau)^{\alpha+1-n}} d\tau. \tag{S5} $$

We note that this definition is also determined by the value of the lower limit a. To complement the previous definition we establish ${}_aD_t^0 = I$, so that the zeroth-order differential operator is the identity operator and ${}_aD_t^0 f(t) = f(t)$. Thus, the integer-order Caputo fractional derivative coincides with the integer-order classical derivative ${}_aD_t^m f(t) = f^m(t)$ for $m \in \mathbb{Z}^+$.

According to the memory interpretation of the fractional integral operator of order α, we extend the memory interpretation to the Caputo fractional derivative definition determined by (S4): values of $\alpha \approx (n-1)^+$ represent more memory effect, whereas values of $\alpha \approx n^-$ represent less memory effect. The specific memory effect is also determined by the value $n \in \mathbb{Z}^+$, which establishes the fractional derivative of order α. In the main manuscript, we consider two different cases for fractional-order α, $0 < \alpha < 1$ and $1 < \alpha < 2$. Due to our previous discussion, memory interpretation is different in both cases. In the case of $0 < \alpha < 1$, we have less memory effect for the case of $\alpha \approx 1^-$ and more memory effect for the case of $\alpha \approx 0^+$. In the case of $1 < \alpha < 2$, we have less memory effect for the case of $\alpha \approx 2^-$ and more memory effect for the case of $\alpha \approx 1^+$. In particular, when considering $\alpha \approx 1$, we have different memory effects for nearby values. For values $\alpha \approx 1^-$, we have less memory effect (from the first-order derivative) and for values $\alpha \approx 1^+$, we have more memory effect (from the second-order derivative).

The following properties of the Caputo's fractional-order derivative are important:

THEOREM 1. *Let f and g be functions of class $\mathcal{C}$ and let $c \in \mathbb{R}$. Then the following properties hold (Podlubny, 1999; Ross, 1974):*

1. ${}_aD_t^\alpha c = 0.$
2. ${}_aD_t^\alpha (f(t) + g(t)) = {}_aD_t^\alpha f(t) + {}_aD_t^\alpha g(t).$
3. ${}_aD_t^\alpha (cf(t)) = c\,{}_aD_t^\alpha f(t).$

There is a simple expression of the Caputo fractional derivative of the term $(t-a)^\mu$ in the form:

$$ {}_aD_t^\alpha (t-a)^\mu = \frac{\Gamma(\mu+1)}{\Gamma(\mu-\alpha+1)}(t-a)^{\mu-\alpha}, \tag{S6}$$

where the expression is valid for μ is such that $0 \le n-1 < \alpha \le n < \mu$ (for details see (Ishteva, 2005)).

The fractional integral operator ${}_aJ_t^\alpha$ and the Caputo fractional derivative operator ${}_aD_t^\alpha$ satisfy the following important properties:

$$ {}_aD_t^\alpha\, {}_aJ_t^\alpha f(t) = f(t), \text{ for } \alpha > 0, \text{ and } t > a, \tag{S7}$$

$$ {}_aJ_t^\alpha\, {}_aD_t^\alpha f(t) = f(t) - \sum_{k=0}^{n-1} f^k(a^+)\frac{(t-a)^k}{k!}, \tag{S8}$$

the last expression is valid for $n-1 < \alpha \le n$, $n \in \mathbb{Z}^+$.

We note that expression (S8) requires the values of the function and its integer derivatives at the point a (or a^+ when necessary).

In addition to the definition of the Caputo fractional derivative, there are a number of different definitions of fractional derivatives (Podlubny, 1999; Ross, 1974). In each of these definitions, the memory effect due to the fractional derivative is encapsulated in its definition. In (Wang and Li, 2011), it is suggested that this provides the possibility of choosing the memory-dependent type according to the needs of the applications. Also, since under convenient hypothesis there is a relationship between the Caputo derivative, the Riemann-Liouville derivative and the Grünwald-Letnikov derivative, it is possible to rewrite the model under a different fractional-order derivative definition (Podlubny, 1999; Ross, 1974).

In the main manuscript of this work, we focus our efforts on the definition of the Caputo fractional derivative, as this definition helped us to simplify our error estimates. Also, we set the lower limit at $a = 0$ to facilitate our computations. However, a further study using different definitions for fractional derivatives, as well as different memory interpretations, might be of interest. For our work we define:

$$D_t^\alpha f(t) := \ {}_0D_t^\alpha f(t), \tag{S9}$$

and

$$J_t^\alpha f(t) := \ {}_0J_t^\alpha f(t). \tag{S10}$$

2 TRAVELING WAVE SOLUTIONS IN THE INTEGER ORDER CASE $\alpha = 1$

In the main manuscript, we established the traveling wave solutions for the traditional voltage-based neural field model. Here, we sketch the procedure to obtain equations (2) and (3) of the main manuscript. First, we consider again the neural field model:

$$\begin{aligned} D_t u\left(x,t\right) &= -u\left(x,t\right) + \int_{-\infty}^{\infty} g(x-y)H(u(y,t)-k)dy - \beta q\left(x,t\right) \\ D_t q\left(x,t\right) &= \epsilon u\left(x,t\right) - \epsilon q\left(x,t\right). \end{aligned} \tag{S11}$$

We begin by changing coordinates into the moving frame (z, t), where $z = x + ct$:

$$\begin{aligned} cD_z u\left(z,t\right) + D_t u\left(z,t\right) &= -u\left(z,t\right) + \int_{-\infty}^{\infty} g(z-s)H(u(s,t)-k)ds - \beta q\left(z,t\right) \\ cD_z q\left(z,t\right) + D_t q\left(z,t\right) &= \epsilon u\left(z,t\right) - \epsilon q\left(z,t\right). \end{aligned} \tag{S12}$$

We then consider stationary solutions in this frame, i.e., $D_t u(z,t) = 0$ and $D_t q(z,t) = 0$. This assumption implies that $u(z,t) = u(z)$ and $q(z,t) = q(z)$. Substituting in the previous system we obtain:

$$\begin{aligned} cD_z u\left(z\right) &= -u\left(z\right) + \int_{-\infty}^{\infty} g(z-s)H(u(s)-k)ds - \beta q\left(z\right) \\ cD_z q\left(z\right) &= \epsilon u\left(z\right) - \epsilon q\left(z\right). \end{aligned} \tag{S13}$$

The previous system is an ordinary differential equations system. We can rewrite the derivatives simply as $D_z u(z) = u'(z)$ and $D_z q(z) = q'(z)$, obtaining:

$$\begin{aligned} u'(z) &= -\left(\frac{1}{c}\right) u(z) + \left(\frac{1}{c}\right) \int_{-\infty}^{\infty} g(z-s) H(u(s)-k) ds - \left(\frac{\beta}{c}\right) q(z) \\ q'(z) &= \left(\frac{\epsilon}{c}\right) u(z) - \left(\frac{\epsilon}{c}\right) q(z). \end{aligned} \tag{S14}$$

The previous system is a constant coefficient non-homogeneous system that can be written in matrix form as:

$$\begin{pmatrix} u'(z) \\ q'(z) \end{pmatrix} = A \begin{pmatrix} u(z) \\ q(z) \end{pmatrix} + \begin{pmatrix} \frac{1}{c} \int_{-\infty}^{\infty} g(z-s) H(u(s)-k) ds \\ 0 \end{pmatrix} \tag{S15}$$

where

$$A = \begin{pmatrix} -\frac{1}{c} & -\frac{\beta}{c} \\ \frac{\epsilon}{c} & -\frac{\epsilon}{c} \end{pmatrix}. \tag{S16}$$

We assume that we have a pulse solution, so that the synaptic threshold is achieved in exactly two points at $z = w_0$, and $z = w$. Given than the wave solution is translationally invariant, we can set $w_0 = 0$. Under these assumptions the previous system simplifies as:

$$\begin{pmatrix} u'(z) \\ q'(z) \end{pmatrix} = A \begin{pmatrix} u(z) \\ q(z) \end{pmatrix} + \begin{pmatrix} \frac{1}{c} \int_{0}^{w} g(z-s) ds \\ 0 \end{pmatrix}. \tag{S17}$$

The previous system can be solved by variation of parameters formula to obtain:

$$\begin{pmatrix} u(z) \\ q(z) \end{pmatrix} = e^{Az} \left(\begin{pmatrix} u(z_0) \\ q(z_0) \end{pmatrix} + \int_{z_0}^{z} e^{-As} \begin{pmatrix} \frac{1}{c} \int_{0}^{w} g(y-s) ds \\ 0 \end{pmatrix} dy \right). \tag{S18}$$

where e^{Az} represents the matrix exponential of Az. Here, we only consider the case of real eigenvalues in the matrix A, which is determined by the parameter relationship $(\epsilon - 1)^2 - 4\epsilon\beta > 0$. Simplifying the above expressions and considering proper conditions at infinity we arrive at the solutions expressed in (2) and (3):

$$\begin{aligned} &u(z) = u(x+ct, t) = \\ &\left(\frac{\epsilon - 1 + \sqrt{(\epsilon-1)^2 - 4\epsilon\beta}}{2c\sqrt{(\epsilon-1)^2 - 4\epsilon\beta}} \right) e^{\lambda_+ (x+ct)} \int_{-\infty}^{x+ct} e^{-s\lambda_+} \left(\int_0^w g(s-y)\, dy \right) ds \\ &- \left(\frac{\epsilon - 1 - \sqrt{(\epsilon-1)^2 - 4\epsilon\beta}}{2c\sqrt{(\epsilon-1)^2 - 4\epsilon\beta}} \right) e^{\lambda_- (x+ct)} \int_{-\infty}^{x+ct} e^{-s\lambda_-} \left(\int_0^w g(s-y)\, dy \right) ds, \end{aligned} \tag{S19}$$

and

$$
\begin{aligned}
&q\left(z\right)=q\left(x+ct,t\right)= \\
&\left(\frac{\epsilon}{c\sqrt{\left(\epsilon-1\right)^{2}-4\epsilon\beta}}\right)e^{\lambda_{+}\left(x+ct\right)}\int_{-\infty}^{x+ct}e^{-s\lambda_{+}}\left(\int_{0}^{w}g\left(s-y\right)dy\right)ds \\
&-\left(\frac{\epsilon}{c\sqrt{\left(\epsilon-1\right)^{2}-4\epsilon\beta}}\right)e^{\lambda_{-}\left(x+ct\right)}\int_{-\infty}^{x+ct}e^{-s\lambda_{-}}\left(\int_{0}^{w}g\left(s-y\right)dy\right)ds
\end{aligned}
\tag{S20}
$$

where

$$
\lambda_{\pm}=-\frac{\epsilon+1}{2c}\pm\frac{\sqrt{\left(\epsilon-1\right)^{2}-4\epsilon\beta}}{2c}. \tag{S21}
$$

3 EXPLICIT MITTAG-LEFFLER APPROXIMATE TRAVELING WAVE SOLUTIONS IN THE CASE $0<\alpha<1$ AND $1<\alpha<2$

The explicit approximate traveling wave solutions in the case of $0<\alpha<1$, $u_{\star R}(x,t)$ and $q_{\star R}(x,t)$ in the main manuscript, are determined by:

$$
u_{\star L}(x,t)=\begin{cases}
A_uE_{\alpha,1}\left(\frac{x+ct^{\alpha}}{\sigma}\right) \\
-A_uE_{\alpha,1}\left(\frac{(x+ct^{\alpha})-w}{\sigma}\right) \\
\qquad\qquad\text{if } x+ct\leq 0 \\
\\
B_uE_{\alpha,1}\left(\lambda_{+}(x+ct^{\alpha})\right) \\
+C_uE_{\alpha,1}\left(\lambda_{-}(x+ct^{\alpha})\right) \\
+D_uE_{\alpha,1}\left(-\frac{(x+ct^{\alpha})}{\sigma}\right) \\
+E_uE_{\alpha,1}\left(\frac{x+ct^{\alpha}-w}{\sigma}\right)+F_u \\
\qquad\qquad\text{if } 0<x+ct\leq w \\
\\
G_uE_{\alpha,1}\left(\lambda_{+}(x+ct^{\alpha})\right) \\
-G_uE_{\alpha,1}\left(\lambda_{+}(x+ct^{\alpha}-w)\right) \\
+H_uE_{\alpha,1}\left(\lambda_{-}(x+ct^{\alpha})\right) \\
-H_uE_{\alpha,1}\left(\lambda_{-}(x+ct^{\alpha}-w)\right) \\
+I_uE_{\alpha,1}\left(-\frac{(x+ct^{\alpha})}{\sigma}\right) \\
+J_uE_{\alpha,1}\left(-\frac{(x+ct^{\alpha}-w)}{\sigma}\right) \\
\qquad\qquad\text{if } x+ct>w
\end{cases}
\tag{S22}
$$

and by

$$
q_{\star L}(x,t) = \begin{cases}
A_q E_{\alpha,1}\left(\frac{x+ct^{\alpha}}{\sigma}\right) \\
-A_q E_{\alpha,1}\left(\frac{(x+ct^{\alpha})-w}{\sigma}\right) \\
\qquad\qquad\qquad \text{if } x+ct \leq 0 \\
\\
B_q E_{\alpha,1}\left(\lambda_{+}(x+ct^{\alpha})\right) \\
+C_q E_{\alpha,1}\left(\lambda_{-}(x+ct^{\alpha})\right) \\
+D_q E_{\alpha,1}\left(-\frac{(x+ct^{\alpha})}{\sigma}\right) \\
+E_q E_{\alpha,1}\left(\frac{x+ct^{\alpha}-w}{\sigma}\right) + F_q \\
\qquad\qquad\qquad \text{if } 0 < x+ct \leq w \\
\\
G_q E_{\alpha,1}\left(\lambda_{+}(x+ct^{\alpha})\right) \\
-G_q E_{\alpha,1}\left(\lambda_{+}(x+ct^{\alpha}-w)\right) \\
+H_q E_{\alpha,1}\left(\lambda_{-}(x+ct^{\alpha})\right) \\
-H_q E_{\alpha,1}\left(\lambda_{-}(x+ct^{\alpha}-w)\right) \\
+I_q E_{\alpha,1}\left(-\frac{(x+ct^{\alpha})}{\sigma}\right) \\
+J_q E_{\alpha,1}\left(-\frac{(x+ct^{\alpha}-w)}{\sigma}\right) \\
\qquad\qquad\qquad \text{if } x+ct > w.
\end{cases} \tag{S23}
$$

The explicit approximate traveling wave solutions in the case of $1 < \alpha < 2$, $u_{\star L}(x,t)$ and $q_{\star L}(x,t)$ in the main manuscript, are determined by:

$$u_{\star R}(x,t) = \begin{cases} \frac{A_u}{2} E_{\alpha,1}\left(\frac{x+ct^\alpha}{\sigma}\right) \\ +\frac{A_u}{2}\left(\frac{x+ct^\alpha}{\sigma}\right) E_{\alpha,2}\left(\frac{x+ct^\alpha}{\sigma}\right) \\ -\frac{A_u}{2} E_{\alpha,1}\left(\frac{x+ct^\alpha-w}{\sigma}\right) \\ -\frac{A_u}{2}\left(\frac{x+ct^\alpha-w}{\sigma}\right) E_{\alpha,2}\left(\frac{x+ct^\alpha-w}{\sigma}\right) \\ \qquad\qquad\qquad \text{if } x+ct \leq 0 \\ \\ \frac{B_u}{2} E_{\alpha,1}\left(\lambda_+(x+ct^\alpha)\right) \\ +\frac{B_u}{2}\left(\lambda_+(x+ct^\alpha)\right) E_{\alpha,2}\left(\lambda_+(x+ct^\alpha)\right) \\ +\frac{C_u}{2} E_{\alpha,1}\left(\lambda_-(x+ct^\alpha)\right) \\ +\frac{C_u}{2}\left(\lambda_-(x+ct^\alpha)\right) E_{\alpha,2}\left(\lambda_-(x+ct^\alpha)\right) \\ +\frac{D_u}{2} E_{\alpha,1}\left(-\frac{x+ct^\alpha}{\sigma}\right) \\ +\frac{D_u}{2}\left(-\frac{x+ct^\alpha}{\sigma}\right) E_{\alpha,2}\left(-\frac{x+ct^\alpha}{\sigma}\right) \\ +\frac{E_u}{2} E_{\alpha,1}\left(\frac{x+ct^\alpha-w}{\sigma}\right) \\ +\frac{E_u}{2}\left(\frac{x+ct^\alpha-w}{\sigma}\right) E_{\alpha,2}\left(\frac{x+ct^\alpha-w}{\sigma}\right) \\ +\frac{F_u}{2} \\ \qquad\qquad\qquad \text{if } 0 < x+ct \leq w \\ \\ \frac{G_u}{2} E_{\alpha,1}\left(\lambda_+(x+ct^\alpha)\right) \\ +\frac{G_u}{2}\left(\lambda_+(x+ct^\alpha)\right) E_{\alpha,2}\left(\lambda_+(x+ct^\alpha)\right) \\ -\frac{G_u}{2} E_{\alpha,1}\left(\lambda_+(x+ct^\alpha-w)\right) \\ -\frac{G_u}{2}\left(\lambda_+(x+ct^\alpha-w)\right) E_{\alpha,2}\left(\lambda_+(x+ct^\alpha-w)\right) \\ +\frac{H_u}{2} E_{\alpha,1}\left(\lambda_-(x+ct^\alpha)\right) \\ +\frac{H_u}{2}\left(\lambda_-(x+ct^\alpha)\right) E_{\alpha,2}\left(\lambda_-(x+ct^\alpha)\right) \\ -\frac{H_u}{2} E_{\alpha,1}\left(\lambda_-(x+ct^\alpha-w)\right) \\ -\frac{H_u}{2}\left(\lambda_-(x+ct^\alpha-w)\right) E_{\alpha,2}\left(\lambda_-(x+ct^\alpha-w)\right) \\ +\frac{I_u}{2} E_{\alpha,1}\left(-\frac{x+ct^\alpha}{\sigma}\right) \\ +\frac{I_u}{2}\left(-\frac{x+ct^\alpha}{\sigma}\right) E_{\alpha,2}\left(-\frac{x+ct^\alpha}{\sigma}\right) \\ +\frac{J_u}{2} E_{\alpha,1}\left(-\frac{x+ct^\alpha-w}{\sigma}\right) \\ +\frac{J_u}{2}\left(-\frac{x+ct^\alpha-w}{\sigma}\right) E_{\alpha,2}\left(-\frac{x+ct^\alpha-w}{\sigma}\right) \\ \qquad\qquad\qquad \text{if } x+ct > w, \end{cases} \tag{S24}$$

and by

$$
q_{\star R}(x,t) = \begin{cases}
\frac{A_q}{2}E_{\alpha,1}\left(\frac{x+ct^\alpha}{\sigma}\right) \\
+\frac{A_q}{2}\left(\frac{x+ct^\alpha}{\sigma}\right)E_{\alpha,2}\left(\frac{x+ct^\alpha}{\sigma}\right) \\
-\frac{A_q}{2}E_{\alpha,1}\left(\frac{x+ct^\alpha-w}{\sigma}\right) \\
-\frac{A_q}{2}\left(\frac{x+ct^\alpha-w}{\sigma}\right)E_{\alpha,2}\left(\frac{x+ct^\alpha-w}{\sigma}\right) \\
\qquad \text{if } x+ct \le 0 \\
\\
\frac{B_q}{2}E_{\alpha,1}\left(\lambda_+(x+ct^\alpha)\right) \\
+\frac{B_q}{2}\left(\lambda_+(x+ct^\alpha)\right)E_{\alpha,2}\left(\lambda_+(x+ct^\alpha)\right) \\
+\frac{C_q}{2}E_{\alpha,1}\left(\lambda_-(x+ct^\alpha)\right) \\
+\frac{C_q}{2}\left(\lambda_-(x+ct^\alpha)\right)E_{\alpha,2}\left(\lambda_-(x+ct^\alpha)\right) \\
+\frac{D_q}{2}E_{\alpha,1}\left(-\frac{x+ct^\alpha}{\sigma}\right) \\
+\frac{D_q}{2}\left(-\frac{x+ct^\alpha}{\sigma}\right)E_{\alpha,2}\left(-\frac{x+ct^\alpha}{\sigma}\right) \\
+\frac{E_q}{2}E_{\alpha,1}\left(\frac{x+ct^\alpha-w}{\sigma}\right) \\
+\frac{E_q}{2}\left(\frac{x+ct^\alpha-w}{\sigma}\right)E_{\alpha,2}\left(\frac{x+ct^\alpha-w}{\sigma}\right) \\
+\frac{F_q}{2} \\
\qquad \text{if } 0 < x+ct \le w \\
\\
\frac{G_q}{2}E_{\alpha,1}\left(\lambda_+(x+ct^\alpha)\right) \\
+\frac{G_q}{2}\left(\lambda_+(x+ct^\alpha)\right)E_{\alpha,2}\left(\lambda_+(x+ct^\alpha)\right) \\
-\frac{G_q}{2}E_{\alpha,1}\left(\lambda_+(x+ct^\alpha-w)\right) \\
-\frac{G_q}{2}\left(\lambda_+(x+ct^\alpha-w)\right)\times \\
E_{\alpha,2}\left(\lambda_+(x+ct^\alpha-w)\right) \\
+\frac{H_q}{2}E_{\alpha,1}\left(\lambda_-(x+ct^\alpha)\right) \\
+\frac{H_q}{2}\left(\lambda_-(x+ct^\alpha)\right)E_{\alpha,2}\left(\lambda_-(x+ct^\alpha)\right) \\
-\frac{H_q}{2}E_{\alpha,1}\left(\lambda_-(x+ct^\alpha-w)\right) \\
-\frac{H_q}{2}\left(\lambda_-(x+ct^\alpha-w)\right)\times \\
E_{\alpha,2}\left(\lambda_-(x+ct^\alpha-w)\right) \\
+\frac{I_q}{2}E_{\alpha,1}\left(-\frac{x+ct^\alpha}{\sigma}\right) \\
+\frac{I_q}{2}\left(-\frac{x+ct^\alpha}{\sigma}\right)E_{\alpha,2}\left(-\frac{x+ct^\alpha}{\sigma}\right) \\
+\frac{J_q}{2}E_{\alpha,1}\left(-\frac{x+ct^\alpha-w}{\sigma}\right) \\
\frac{J_q}{2}\left(-\frac{x+ct^\alpha-w}{\sigma}\right)E_{\alpha,2}\left(-\frac{x+ct^\alpha-w}{\sigma}\right) \\
\qquad \text{if } x+ct > w.
\end{cases} \tag{S25}
$$

The coefficients for the traveling wave solutions (S22),(S23), (S24) and (S25) as well as the coefficients for (5) and (6) in the main manuscript depend on the different model parameters and are determined by:

$$A_u = \frac{\sigma}{4c\sqrt{(\epsilon-1)^2-4\epsilon\beta}} \left(\frac{\epsilon - 1 + \sqrt{(\epsilon-1)^2-4\epsilon\beta}}{1-\lambda_+\sigma} - \frac{\epsilon - 1 - \sqrt{(\epsilon-1)^2-4\epsilon\beta}}{1-\lambda_-\sigma} \right) \quad , \tag{S26}$$

$$B_u = \frac{\epsilon - 1 + \sqrt{(\epsilon-1)^2-4\epsilon\beta}}{4c\sqrt{(\epsilon-1)^2-4\epsilon\beta}} \left(\frac{\sigma}{1-\lambda_+\sigma} - \frac{\sigma}{1+\lambda_+\sigma} + \frac{2}{\lambda_+} \right), \tag{S27}$$

$$C_u = \frac{\epsilon - 1 - \sqrt{(\epsilon-1)^2-4\epsilon\beta}}{4c\sqrt{(\epsilon-1)^2-4\epsilon\beta}} \left(\frac{\sigma}{1+\lambda_-\sigma} - \frac{\sigma}{1-\lambda_-\sigma} - \frac{2}{\lambda_-} \right), \tag{S28}$$

$$D_u = \frac{\sigma}{4c\sqrt{(\epsilon-1)^2-4\epsilon\beta}} \left(\frac{\epsilon - 1 + \sqrt{(\epsilon-1)^2-4\epsilon\beta}}{1+\lambda_+\sigma} - \frac{\epsilon - 1 - \sqrt{(\epsilon-1)^2-4\epsilon\beta}}{1+\lambda_-\sigma} \right), \tag{S29}$$

$$E_u = \frac{\sigma}{4c\sqrt{(\epsilon-1)^2-4\epsilon\beta}} \left(\frac{\epsilon - 1 - \sqrt{(\epsilon-1)^2-4\epsilon\beta}}{1-\lambda_-\sigma} - \frac{\epsilon - 1 + \sqrt{(\epsilon-1)^2-4\epsilon\beta}}{1-\lambda_+\sigma} \right), \tag{S30}$$

$$F_u = \frac{1}{2c\sqrt{(\epsilon-1)^2-4\epsilon\beta}} \left(\frac{\epsilon - 1 - \sqrt{(\epsilon-1)^2-4\epsilon\beta}}{\lambda_-} - \frac{\epsilon - 1 + \sqrt{(\epsilon-1)^2-4\epsilon\beta}}{\lambda_+} \right), \tag{S31}$$

$$G_u = \frac{\epsilon - 1 + \sqrt{(\epsilon-1)^2-4\epsilon\beta}}{4c\sqrt{(\epsilon-1)^2-4\epsilon\beta}} \left(\frac{\sigma}{1-\lambda_+\sigma} + \frac{2}{\lambda_+} - \frac{\sigma}{1+\lambda_+\sigma} \right), \tag{S32}$$

$$H_u = \frac{\epsilon - 1 - \sqrt{(\epsilon-1)^2-4\epsilon\beta}}{4c\sqrt{(\epsilon-1)^2-4\epsilon\beta}} \left(\frac{\sigma}{1+\lambda_-\sigma} - \frac{2}{\lambda_-} - \frac{\sigma}{1-\lambda_-\sigma} \right), \tag{S33}$$

$$I_u = \frac{\sigma}{4c\sqrt{(\epsilon-1)^2-4\epsilon\beta}} \left(\frac{\epsilon - 1 + \sqrt{(\epsilon-1)^2-4\epsilon\beta}}{1+\lambda_+\sigma} - \frac{\epsilon - 1 - \sqrt{(\epsilon-1)^2-4\epsilon\beta}}{1+\lambda_-\sigma} \right), \tag{S34}$$

$$J_u = \frac{\sigma}{4c\sqrt{(\epsilon-1)^2-4\epsilon\beta}} \left(\frac{\epsilon - 1 - \sqrt{(\epsilon-1)^2-4\epsilon\beta}}{1+\lambda_-\sigma} - \frac{\epsilon - 1 + \sqrt{(\epsilon-1)^2-4\epsilon\beta}}{1+\lambda_+\sigma} \right), \tag{S35}$$

$$A_q = \frac{\sigma\epsilon}{2c\sqrt{(\epsilon-1)^2-4\epsilon\beta}} \left(\frac{1}{1-\lambda_+\sigma} - \frac{1}{1-\lambda_-\sigma} \right), \tag{S36}$$

$$B_q = \frac{\epsilon}{2c\sqrt{(\epsilon-1)^2-4\epsilon\beta}} \left(\frac{\sigma}{1-\lambda_+\sigma} - \frac{\sigma}{1+\lambda_+\sigma} + \frac{2}{\lambda_+} \right), \tag{S37}$$

$$C_q = \frac{\epsilon}{2c\sqrt{(\epsilon-1)^2-4\epsilon\beta}} \left(\frac{\sigma}{1+\lambda_-\sigma} - \frac{\sigma}{1-\lambda_-\sigma} - \frac{2}{\lambda_-} \right), \tag{S38}$$

$$D_q = \frac{\epsilon}{2c\sqrt{(\epsilon-1)^2-4\epsilon\beta}} \left(\frac{\sigma}{1+\lambda_+\sigma} - \frac{\sigma}{1+\lambda_-\sigma} \right), \tag{S39}$$

$$E_q = \frac{\epsilon}{2c\sqrt{(\epsilon-1)^2-4\epsilon\beta}}\left(\frac{\sigma}{1-\lambda_-\sigma}-\frac{\sigma}{1-\lambda_+\sigma}\right), \tag{S40}$$

$$F_q = \frac{\epsilon}{c\sqrt{(\epsilon-1)^2-4\epsilon\beta}}\left(\frac{1}{\lambda_-}-\frac{1}{\lambda_+}\right), \tag{S41}$$

$$G_q = \frac{\epsilon}{2c\sqrt{(\epsilon-1)^2-4\epsilon\beta}}\left(\frac{\sigma}{1-\lambda_+\sigma}+\frac{2}{\lambda_+}-\frac{\sigma}{1+\lambda_+\sigma}\right), \tag{S42}$$

$$H_q = \frac{\epsilon}{2c\sqrt{(\epsilon-1)^2-4\epsilon\beta}}\left(\frac{\sigma}{1+\lambda_-\sigma}-\frac{2}{\lambda_-}-\frac{\sigma}{1-\lambda_-\sigma}\right), \tag{S43}$$

$$I_q = \frac{\epsilon\sigma}{2c\sqrt{(\epsilon-1)^2-4\epsilon\beta}}\left(\frac{1}{1+\lambda_+\sigma}-\frac{1}{1+\lambda_-\sigma}\right), \tag{S44}$$

and

$$J_q = \frac{\epsilon\sigma}{2c\sqrt{(\epsilon-1)^2-4\epsilon\beta}}\left(\frac{1}{1+\lambda_-\sigma}-\frac{1}{1+\lambda_+\sigma}\right). \tag{S45}$$

3.1 Synaptic kernels used in Mittag-Leffler pulse approximations of fractional-order neural field models

In section 3 of the main manuscript, we utilized Mittag-Leffler functions to construct fractional-order wave approximate solutions. There, we choose a particular exponential kernel for the first-order neural field model, namely, $g(x) = \frac{1}{2\sigma}e^{-\frac{|x|}{\sigma}}$. In the Mittag-Leffler fractional-order wave approximations, specific kernels are required. That is,

$$g_L(x) = \frac{1}{2\sigma}\frac{d}{du}\left(E_{\alpha,1}u\right)\Big|_{u=-\frac{|x|}{\sigma}} \tag{S46}$$

is chosen for the case of $\alpha \approx 1^-$, and

$$g_R(x) = \frac{1}{4\sigma}\frac{d}{du}\left[E_{\alpha,1}u\right]\Big|_{u=-\frac{|x|}{\sigma}} + \frac{1}{4\sigma}\frac{d}{du}\left[uE_{\alpha,2}u\right]\Big|_{u=-\frac{|x|}{\sigma}} \tag{S47}$$

is used for the case of $\alpha \approx 1^+$.

In Figure S1 we provide an example of the synaptic kernels $g_L(x)$ and $g_R(x)$. It can be proven that $g_L(x) \to g(x)$ as $\alpha \to 1^-$, and that $g_R(x) \to g(x)$ as $\alpha \to 1^+$, so that in both cases we recover the integer-order kernel.

4 ERROR ESTIMATES OF THE FRACTIONAL DERIVATIVE ON THE FRACTIONAL COORDINATE SYSTEM

In this section, we provide the absolute error estimates necessary to develop the wave approximations of section 3 of the main manuscript. These error estimates support the assumptions of a chain rule-like behaviour for values of $\alpha \approx 1$. These estimates are based on: wave speed c, synaptic connectivity range σ, fractional derivative order α, distance x, and time t. Therefore, for each wave solution with a fixed value of

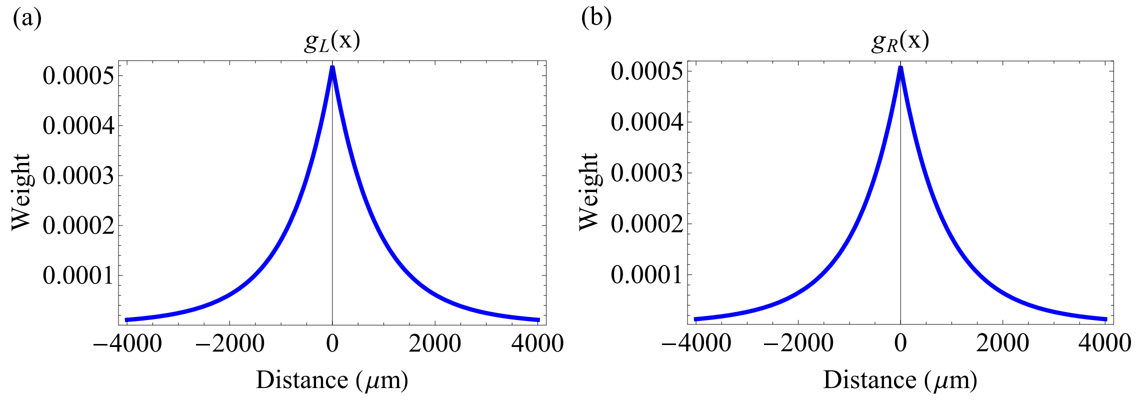

Figure S1. Kernels for the fractional-order neural field models. **(a)** $g_L(x)$ for $\sigma = 1000$ μm **(b)** $g_R(x)$ for $\sigma = 1000$ μm.

speed and width there is a different corresponding error estimate. In the case of $\alpha \approx 1^-$, we provided the fractional derivative approximation:

$$D_t^\alpha E_{\alpha,1}\left(\frac{x+ct^\alpha}{\sigma}\right) \approx \frac{c}{\sigma} E_{\alpha,1}\left(\frac{x+ct^\alpha}{\sigma}\right). \tag{S48}$$

The previous approximation is also used in in the case $\alpha \approx 1^+$. We now provide an absolute error estimate of (S48), applicable for both $\alpha \approx 1^-$ and $\alpha \approx 1^+$, as follows:

$$\begin{aligned}
&\left|D_t^\alpha E_{\alpha,1}\left(\frac{x+ct^\alpha}{\sigma}\right) - \frac{c}{\sigma}E_{\alpha,1}\left(\frac{x+ct^\alpha}{\sigma}\right)\right| \leq \\
&\frac{\left|1-\alpha\right|c(x+ct^\alpha)}{\sigma^2}\left(E_{\alpha,2\alpha-1}\left(\frac{x+ct^\alpha}{\sigma}\right)+\right. \\
&\left.\left|1-\alpha\right|E_{\alpha,2\alpha}\left(\frac{x+ct^\alpha}{\sigma}\right)\right) + \frac{c}{\sigma}\left|\Gamma(\alpha)-1\right|E_{\alpha,\alpha}\left(\frac{x}{\sigma}\right) - \\
&\frac{\left|1-\alpha\right|cx}{\sigma^2}\left(E_{\alpha,2\alpha-1}\left(\frac{x}{\sigma}\right)+\left|1-\alpha\right|E_{\alpha,2\alpha}\left(\frac{x}{\sigma}\right)\right) + \\
&\frac{c}{\sigma}\left|E_{\alpha,\alpha}\left(\frac{x+ct^\alpha}{\sigma}\right) - E_{\alpha,1}\left(\frac{x+ct^\alpha}{\sigma}\right)\right|.
\end{aligned} \tag{S49}$$

The previous estimation can be obtained by using the fractional-order derivative definition, performing algebraic manipulation, and using Gautschi's inequality ($\alpha \approx 1^-$) or Wendel's double inequality ($\alpha \approx 1^+$). We now provide a sketch of the previous procedure. First, by using the triangle inequality, we consider:

$$
\begin{aligned}
&\Big|D_t^{\alpha} E_{\alpha,1}\left(\frac{x+ct^{\alpha}}{\sigma}\right) - \frac{c}{\sigma} E_{\alpha,1}\left(\frac{x+ct^{\alpha}}{\sigma}\right)\Big| \leq \\
&\Big|D_t^{\alpha} E_{\alpha,1}\left(\frac{x+ct^{\alpha}}{\sigma}\right) - \frac{c}{\sigma} E_{\alpha,\alpha}\left(\frac{x+ct^{\alpha}}{\sigma}\right)\Big| + \frac{c}{\sigma}\Big|E_{\alpha,\alpha}\left(\frac{x+ct^{\alpha}}{\sigma}\right) - E_{\alpha,1}\left(\frac{x+ct^{\alpha}}{\sigma}\right)\Big|.
\end{aligned}
\tag{S50}
$$

We notice that the second term of the previous inequality is exactly the last term in (S49). Therefore, to obtain the rest of the bounding terms in (S49) we need to bound the first term in (S50). By using the definition of the Mittag-Leffler functions and the binomial formula, we rewrite the first term in (S50) as:

$$
\begin{aligned}
&\Big|D_t^{\alpha} E_{\alpha,1}\left(\frac{x+ct^{\alpha}}{\sigma}\right) - \frac{c}{\sigma} E_{\alpha,\alpha}\left(\frac{x+ct^{\alpha}}{\sigma}\right)\Big| \\
&= \Big| D_t^{\alpha} \sum_{k=0}^{\infty} \frac{1}{\Gamma(\alpha k+1)} \sum_{n=0}^{k} \left(\frac{x}{\sigma}\right)^n \left(\frac{c}{\sigma}\right)^{k-n} \binom{k}{n} t^{\alpha(k-n)} \\
&- \left(\frac{c}{\sigma}\right) \sum_{k=0}^{\infty} \frac{1}{\Gamma(\alpha k+\alpha)} \sum_{n=0}^{k} \binom{k}{n} \left(\frac{x}{\sigma}\right)^n t^{\alpha(k-n)} \left(\frac{c}{\sigma}\right)^{\alpha(k-n)} \Big| \\
&= \Big| \sum_{k=1}^{\infty} \frac{1}{\Gamma(\alpha k+1)} \sum_{n=0}^{k-1} \left(\frac{x}{\sigma}\right)^n \left(\frac{c}{\sigma}\right)^{k-n} \binom{k}{n} D_t^{\alpha} t^{\alpha(k-n)} \\
&- \left(\frac{c}{\sigma}\right) \sum_{k=0}^{\infty} \frac{1}{\Gamma(\alpha k+\alpha)} \sum_{n=0}^{k} \binom{k}{n} \left(\frac{x}{\sigma}\right)^n t^{\alpha(k-n)} \left(\frac{c}{\sigma}\right)^{\alpha(k-n)} \Big| \\
&= \Big| \sum_{k=1}^{\infty} \frac{\alpha c k}{\sigma \Gamma(\alpha k+1)} \sum_{n=0}^{k-1} \left(\frac{x}{\sigma}\right)^n \left(\frac{c}{\sigma}\right)^{k-n-1} \binom{k-1}{n} t^{\alpha(k-n-1)} \frac{\Gamma(\alpha(k-n))}{\Gamma(\alpha(k-n)+(1-\alpha))} \\
&- \left(\frac{c}{\sigma}\right) \sum_{k=0}^{\infty} \frac{1}{\Gamma(\alpha k+\alpha)} \sum_{n=0}^{k} \binom{k}{n} \left(\frac{x}{\sigma}\right)^n t^{\alpha(k-n)} \left(\frac{c}{\sigma}\right)^{\alpha(k-n)} \Big|.
\end{aligned}
\tag{S51}
$$

The last term has been obtained by using the fractional derivative formula (S6) and simplifying by using a property of the Gamma function that states $\Gamma(\alpha+1) = \alpha\Gamma(\alpha)$ (see Podlubny (1999) for details). The last term of (S51) can be further simplified by making a change of variables in the first summation to obtain:

$$
\begin{aligned}
&\Big| \sum_{k=0}^{\infty} \frac{c}{\sigma \Gamma(\alpha k+\alpha)} \sum_{n=0}^{k} \left(\frac{x}{\sigma}\right)^n \left(\frac{c}{\sigma}\right)^{k-n} \binom{k}{n} t^{\alpha(k-n)} \frac{\Gamma(\alpha(k-n)+\alpha)}{\Gamma(\alpha(k-n)+1)} \\
&- \sum_{k=0}^{\infty} \frac{c}{\sigma \Gamma(\alpha k+\alpha)} \sum_{n=0}^{k} \binom{k}{n} \left(\frac{x}{\sigma}\right)^n t^{\alpha(k-n)} \left(\frac{c}{\sigma}\right)^{\alpha(k-n)} \Big| \\
&\leq \sum_{k=0}^{\infty} \frac{c}{\sigma \Gamma(\alpha k+\alpha)} \sum_{n=0}^{k} \left(\frac{x}{\sigma}\right)^n \left(\frac{c}{\sigma}\right)^{k-n} \binom{k}{n} t^{\alpha(k-n)} \Big| \frac{\Gamma(\alpha(k-n)+\alpha)}{\Gamma(\alpha(k-n)+1)} - 1 \Big|.
\end{aligned}
\tag{S52}
$$

The last term is obtained by noticing that we have manipulated the indexes and exponents of the two summations to be equal. By using Gautschi's inequality ($\alpha \approx 1^-$), or Wendel's double inequality ($\alpha \approx 1^+$) and considering $m \geq 1$, it can be obtained the following bound:

$$\left|\frac{\Gamma(\alpha m+\alpha)}{\Gamma(\alpha m+1)}-1\right| \leq \left|1-\alpha\right|\alpha m \leq \left|\alpha-1\right|\alpha m_{max}, \tag{S53}$$

where m_{max} is the maximum finite index considered. By conveniently manipulating the last term in (S52), and by using (S53), we can bound it as:

$$\begin{aligned}
&\sum_{k=0}^{\infty}\frac{c}{\sigma\Gamma(\alpha k+\alpha)}\left(\sum_{n=0}^{k-1}\left(\frac{x}{\sigma}\right)^{n}\left(\frac{c}{\sigma}\right)^{k-n}\binom{k}{n}t^{\alpha(k-n)}\left|\frac{\Gamma(\alpha(k-n)+\alpha)}{\Gamma(\alpha(k-n)+1)}-1\right|\right)\\
&\leq\sum_{k=0}^{\infty}\frac{c}{\sigma\Gamma(\alpha k+\alpha)}\left(\sum_{n=0}^{k-1}\left(\frac{x}{\sigma}\right)^{n}\left(\frac{c}{\sigma}\right)^{k-n}\binom{k}{n}t^{\alpha(k-n)}\left|1-\alpha\right|\alpha k\right.\\
&\left.+\binom{k}{k}\left(\frac{x}{\sigma}\right)^{k}\left|1-\alpha\right|\alpha k-\binom{k}{k}\left(\frac{x}{\sigma}\right)^{k}\left|1-\alpha\right|\alpha k+\binom{k}{k}\left(\frac{x}{\sigma}\right)^{k}\left|\frac{\Gamma(\alpha)}{\Gamma(1)}-1\right|\right)\\
&=\sum_{k=0}^{\infty}\frac{c}{\sigma\Gamma(\alpha k+\alpha)}\left(\sum_{n=0}^{k}\left(\frac{x}{\sigma}\right)^{n}\left(\frac{c}{\sigma}\right)^{k-n}\binom{k}{n}t^{\alpha(k-n)}\left|1-\alpha\right|\alpha k+\left(\frac{x}{\sigma}\right)^{k}\left(\left|\Gamma(\alpha)-1\right|-\left|1-\alpha\right|\alpha k\right)\right)\\
&=\left(\frac{c}{\sigma}\right)\left|1-\alpha\right|\sum_{k=0}^{\infty}\frac{\alpha k}{\Gamma\left(\alpha(k+1)\right)}\left(\frac{x+ct^{\alpha}}{\sigma}\right)^{k}+\left(\frac{c}{\sigma}\right)\left|\Gamma(\alpha)-1\right|\sum_{k=0}^{\infty}\frac{x^{k}}{\Gamma\left(\alpha k+\alpha\right)}\\
&-\left|1-\alpha\right|\left(\frac{c}{\sigma}\right)\sum_{k=0}^{\infty}\frac{\alpha k}{\Gamma(\alpha k+k)}\left(\frac{x}{\sigma}\right)^{k}\\
&\leq\left(\frac{c}{\sigma}\right)\left|1-\alpha\right|\left(\left(\frac{x+ct^{\alpha}}{\sigma}\right)\sum_{k=0}^{\infty}\frac{(x+ct^{\alpha})^{k}}{\sigma^{k}\Gamma(\alpha k+2\alpha-1)}+\left|1-\alpha\right|\left(\frac{x+ct^{\alpha}}{\sigma}\right)\sum_{k=0}^{\infty}\frac{(x+ct^{\alpha})^{k}}{\Gamma(\alpha k+2\alpha)}\right)\\
&+\left(\frac{c}{\sigma}\right)\left|\Gamma(\alpha)-1\right|E_{\alpha,\alpha}\left(\frac{x}{\sigma}\right)-\left|1-\alpha\right|\left(\frac{c}{\sigma}\right)\left(\frac{x}{\sigma}\right)\sum_{k=0}^{\infty}\frac{\left(\frac{x}{\sigma}\right)^{k}}{\Gamma(\alpha k+2\alpha-1)}+\left|1-\alpha\right|\left(\frac{x}{\sigma}\right)\sum_{k=0}^{\infty}\frac{x^{k}}{\sigma^{k}\Gamma(\alpha k+2\alpha)}\\
&=\left(\frac{c}{\sigma}\right)\left|1-\alpha\right|\left(\frac{x+ct^{\alpha}}{\sigma}\right)\left(E_{\alpha,2\alpha-1}\left(\frac{x+ct^{\alpha}}{\sigma}\right)+\left|1-\alpha\right|E_{\alpha,2\alpha}\left(\frac{x+ct^{\alpha}}{\sigma}\right)\right)\\
&+\left(\frac{c}{\sigma}\right)\left|\Gamma(\alpha)-1\right|E_{\alpha,\alpha}\left(\frac{x}{\sigma}\right)-\left(\frac{c}{\sigma}\right)\left|1-\alpha\right|\left(\frac{x}{\sigma}\right)\left(E_{\alpha,2\alpha-1}\left(\frac{x}{\sigma}\right)+\left|1-\alpha\right|E_{\alpha,2\alpha}\left(\frac{x}{\sigma}\right)\right)
\end{aligned} \tag{S54}$$

To arrive to the last bounding term we have performed algebraic manipulation and use properties of the Gamma function. By using the last bounding terms in (S54) and (S50) we recover the error estimates established in (S49) for $\alpha \approx 1^-$. A similar approach can be developed for $\alpha \approx 1^+$ by using (S53), obtaining similar results.

In this case, we notice that as $\alpha \to 1^-$ each of the bounding terms located in the right hand side of inequality (S49) tend to zero, as a factor of $\mid 1-\alpha \mid$ appears in most of the terms. The last term in (S49) naturally tends to zero as $\alpha \to 1^-$. Therefore, as $\alpha \to 1^-$ the approximation (S48) converges to an inequality. In Figure S2(a), we provide an example of an error estimate obtained from (S49). We observe that we obtain low absolute error estimates in the case of $\alpha \approx 1^-$ when considering small times and small widths.

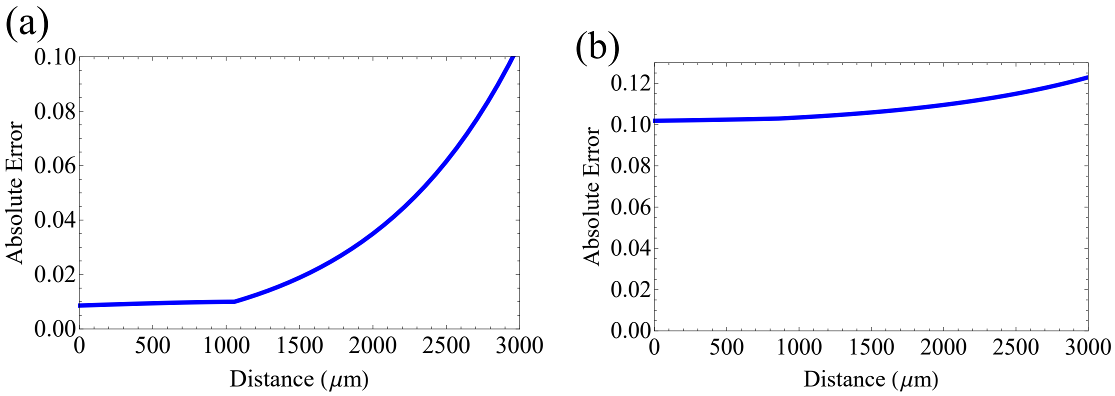

Figure S2. Absolute error estimates of chain-rule-like fractional-order derivative expression under different parameter choices restricted to $\alpha \approx 1$. The error estimates depend on: fractional-order α, synaptic connectivity σ, wave speed c, and elapsed time and distance. **(a)** Error estimates for parameters $\alpha = 0.9$, $c = 100$ μm/ms and $\sigma = 1000$ μm. **(b)** Error estimates for parameters $\alpha = 1.01$, $c = 150$ μm/ms and $\sigma = 1500$ μm. In **(a)-(b)**, we consider small times ($t = 0.1$). In both cases, the approximations improve as $\alpha \to 1$. Our approximations work better in the case of $\alpha \approx 1^-$ than in the case $\alpha \approx 1^+$. This is consistent with the memory interpretation: values of $\alpha \approx 1^-$ represent little memory effect (from the first-order time derivative) and values of $\alpha \approx 1^+$ represent more memory effect (from the second-order time derivative). Also, the absolute error is diminished when considering longer connectivity ranges and slower wave speeds. Parameters fixed for all plots: $\beta = 1$ and $\epsilon = 0.1$.

In the case of $\alpha \approx 1^+$, we provide the fractional derivative approximation:

$$
\begin{aligned}
& D_t^{\alpha} E_{\alpha,1}\left(\frac{x+ct^{\alpha}}{\sigma}\right) + \left(\frac{x+ct^{\alpha}}{\sigma}\right) E_{\alpha,2}\left(\frac{x+ct^{\alpha}}{\sigma}\right) \approx \\
& \frac{c}{\sigma}\left(E_{\alpha,1}\left(\frac{x+ct^{\alpha}}{\sigma}\right) + \left(\frac{x+ct^{\alpha}}{\sigma}\right) E_{\alpha,2}\left(\frac{x+ct^{\alpha}}{\sigma}\right)\right).
\end{aligned}
\tag{S55}
$$

The absolute error estimate of the previous estimation can be written as:

$$
\begin{aligned}
&\Big|D_t^{\alpha}\left(E_{\alpha,1}\left(\frac{x+ct^{\alpha}}{\sigma}\right)+\left(\frac{x+ct^{\alpha}}{\sigma}\right)E_{\alpha,2}\left(\frac{x+ct^{\alpha}}{\sigma}\right)\right)-\\
&\frac{c}{\sigma}\left(E_{\alpha,1}\left(\frac{x+ct^{\alpha}}{\sigma}\right)+\left(\frac{x+ct^{\alpha}}{\sigma}\right)E_{\alpha,2}\left(\frac{x+ct^{\alpha}}{\sigma}\right)\right)\Big|\\
&\leq\Big|D_t^{\alpha}\left(E_{\alpha,1}\left(\frac{x+ct^{\alpha}}{\sigma}\right)-\frac{c}{\sigma}E_{\alpha,1}\left(\frac{x+ct^{\alpha}}{\sigma}\right)\right)\Big|+\\
&\Big|D_t^{\alpha}\left(\left(\frac{x+ct^{\alpha}}{\sigma}\right)E_{\alpha,2}\left(\frac{x+ct^{\alpha}}{\sigma}\right)\right)-\frac{c}{\sigma}\left(\frac{x+ct^{\alpha}}{\sigma}\right)E_{\alpha,2}\left(\frac{x+ct^{\alpha}}{\sigma}\right)\Big|.
\end{aligned}
\tag{S56}
$$

The first term of (S56) has already been bounded by (S49). The second term can be bounded as:

$$
\begin{aligned}
&\Big|D_t^{\alpha}\left(\left(\frac{x+ct^{\alpha}}{\sigma}\right)E_{\alpha,2}\left(\frac{x+ct^{\alpha}}{\sigma}\right)\right)-\frac{c}{\sigma}\left(\frac{x+ct^{\alpha}}{\sigma}\right)E_{\alpha,2}\left(\frac{x+ct^{\alpha}}{\sigma}\right)\Big|\\
&\leq\frac{c(\Gamma(\alpha+1))}{\sigma}+\frac{c(\alpha-1)(x+ct^{\alpha})}{\sigma^2}E_{\alpha,\alpha}\left(\frac{x+ct^{\alpha}}{\sigma}\right)\\
&+\frac{c(\Gamma(\alpha)-1)x}{\sigma^2}E_{\alpha,1+\alpha}\left(\frac{x}{\sigma}\right)-\frac{c(\alpha-1)x}{\sigma^2}E_{\alpha,\alpha}\left(\frac{x}{\sigma}\right)\\
&+\frac{c(\alpha-1)(x+ct^{\alpha})}{\sigma^2}E_{\alpha,2+\alpha}\left(\frac{x+ct^{\alpha}}{\sigma}\right)+\\
&\frac{c(\alpha-1)^2(x+ct^{\alpha})}{\sigma^2}\left(E_{\alpha,\alpha+1}\left(\frac{x+ct^{\alpha}}{\sigma}\right)\right.\\
&\left.-E_{\alpha,\alpha+2}\left(\frac{x+ct^{\alpha}}{\sigma}\right)\right)+\frac{\Gamma(\alpha)(\alpha-1)cx}{\sigma^2}E_{\alpha,2+\alpha}\left(\frac{x}{\sigma}\right)\\
&-\frac{c(\alpha-1)^2x}{\sigma}\left(E_{\alpha,\alpha+1}\left(\frac{x}{\sigma}\right)-E_{\alpha,\alpha+2}\left(\frac{x}{\sigma}\right)\right)+\\
&\frac{c(x+ct^{\alpha})}{\sigma^2}\Big|E_{\alpha,2}\left(\frac{x+ct^{\alpha}}{\sigma}\right)-E_{\alpha,1+\alpha}\left(\frac{x+ct^{\alpha}}{\sigma}\right)\Big|.
\end{aligned}
\tag{S57}
$$

We now provide a sketch of how to obtain the previous inequality. We consider:

$$
\begin{aligned}
&\Big|D_t^{\alpha}\left(\left(\frac{x+ct^{\alpha}}{\sigma}\right)E_{\alpha,2}\left(\frac{x+ct^{\alpha}}{\sigma}\right)\right)-\frac{c}{\sigma}\left(\frac{x+ct^{\alpha}}{\sigma}\right)E_{\alpha,2}\left(\frac{x+ct^{\alpha}}{\sigma}\right)\Big|\\
&\leq\Big|D_t^{\alpha}\left(\left(\frac{x+ct^{\alpha}}{\sigma}\right)E_{\alpha,2}\left(\frac{x+ct^{\alpha}}{\sigma}\right)\right)-\frac{c}{\sigma}\left(\frac{x+ct^{\alpha}}{\sigma}\right)E_{\alpha,1+\alpha}\left(\frac{x+ct^{\alpha}}{\sigma}\right)\Big|\\
&+\frac{c(x+ct^{\alpha})}{\sigma^2}\Big|E_{\alpha,2}\left(\frac{x+ct^{\alpha}}{\sigma}\right)-E_{\alpha,1+\alpha}\left(\frac{x+ct^{\alpha}}{\sigma}\right)\Big|
\end{aligned}
\tag{S58}
$$

We notice that the last term of the previous inequality is the same as the last term in (S57). By using the fractional derivative definition, algebraic manipulation and inequality (S53), the first term of (S58) can be bounded as:

$$
\begin{aligned}
&\Big|D_t^{\alpha}\left(\left(\frac{x+ct^{\alpha}}{\sigma}\right)E_{\alpha,2}\left(\frac{x+ct^{\alpha}}{\sigma}\right)\right)-\frac{c}{\sigma}\left(\frac{x+ct^{\alpha}}{\sigma}\right)E_{\alpha,1+\alpha}\left(\frac{x+ct^{\alpha}}{\sigma}\right)\Big|\\
&=\Big|\frac{c\Gamma(\alpha+1)}{\sigma}+\frac{c}{\sigma}\sum_{k=1}^{\infty}\frac{\alpha k+\alpha}{\Gamma(\alpha k+1)\alpha k+1}\sum_{n=0}^{k}\binom{k}{n}\left(\frac{x}{\sigma}\right)^{k}\left(\frac{c}{\sigma}\right)^{k-n}t^{\alpha(k-n)}\frac{\Gamma(\alpha(k-n)+\alpha)}{\Gamma(\alpha(k-n)+1)}\\
&-\sum_{k=1}^{\infty}\frac{c}{\sigma\Gamma(\alpha k+1)}\sum_{n=0}^{k}\binom{k}{n}\left(\frac{x}{\sigma}\right)^{n}\left(\frac{c}{\sigma}\right)^{k-n}t^{\alpha(k-n)}\Big|\\
&=\Big|\frac{c\Gamma(\alpha+1)}{\sigma}+\frac{c}{\sigma}\sum_{k=1}^{\infty}\frac{1}{\Gamma(\alpha k+1)}\sum_{n=0}^{k}\binom{k}{n}\left(\frac{x}{\sigma}\right)^{n}\left(\frac{c}{\sigma}\right)^{k-n}t^{\alpha(k-n)}\frac{\Gamma(\alpha(k-n)+\alpha)}{\Gamma(\alpha(k-n)+1)}\\
&-\sum_{k=1}^{\infty}\frac{c}{\sigma\Gamma(\alpha k+1)}\sum_{n=0}^{k}\binom{k}{n}\left(\frac{x}{\sigma}\right)^{n}\left(\frac{c}{\sigma}\right)^{k-n}t^{\alpha(k-n)}\\
&+\sum_{k=1}^{\infty}\frac{c(\alpha-1)}{\sigma\Gamma(\alpha k+2)}\sum_{n=0}^{k}\binom{k}{n}\left(\frac{x}{\sigma}\right)^{n}\left(\frac{c}{\sigma}\right)^{k-n}t^{\alpha(k-n)}\frac{\Gamma(\alpha(k-n)+\alpha)}{\Gamma(\alpha(k-n)+1)}\Big|\\
&\leq\frac{c\Gamma(\alpha+1)}{\sigma}\\
&+\sum_{k=1}^{\infty}\frac{c}{\sigma\Gamma(\alpha k+1)}\left(\sum_{n=0}^{k-1}\binom{k}{n}\left(\frac{x}{\sigma}\right)^{n}\left(\frac{c}{\sigma}\right)^{k-n}t^{\alpha(k-n)}\Big|\frac{\Gamma(\alpha(k-n)+\alpha)}{\Gamma(\alpha(k-n)+1)}-1\Big|+\left(\frac{x}{\sigma}\right)^{k}\Big|\Gamma(\alpha)-1\Big|\right)\\
&+\sum_{k=1}^{\infty}\frac{c(\alpha-1)}{\sigma\Gamma(\alpha k+2)}\left(\sum_{n=0}^{k-1}\binom{k}{n}\left(\frac{x}{\sigma}\right)^{n}\left(\frac{c}{\sigma}\right)^{k-n}t^{\alpha(k-n)}\Big|\frac{\Gamma(\alpha(k-n)+\alpha)}{\Gamma(\alpha(k-n)+1)}\Big|+\left(\frac{x}{\sigma}\right)^{k}\Gamma(\alpha)\right)\\
&\leq\frac{c\Gamma(\alpha+1)}{\sigma}\\
&+\sum_{k=1}^{\infty}\frac{c}{\sigma\Gamma(\alpha k+1)}\left(\sum_{n=0}^{k-1}\binom{k}{n}\left(\frac{x}{\sigma}\right)^{n}\left(\frac{c}{\sigma}\right)^{k-n}t^{\alpha(k-n)}(\alpha-1)\alpha k+\left(\frac{x}{\sigma}\right)^{k}\Big|\Gamma(\alpha)-1\Big|\right.\\
&\left.+\left(\frac{x}{\sigma}\right)^{k}(\alpha-1)\alpha k-\left(\frac{x}{\sigma}\right)^{k}(\alpha-1)\alpha k\right)\\
&+\sum_{k=1}^{\infty}\frac{c(\alpha-1)}{\sigma\Gamma(\alpha k+2)}\left(\sum_{n=0}^{k-1}\binom{k}{n}\left(\frac{x}{\sigma}\right)^{n}\left(\frac{c}{\sigma}\right)^{k-n}t^{\alpha(k-n)}(1+(\alpha-1)\alpha k)+\left(\frac{x}{\sigma}\right)^{k}\Gamma(\alpha)\right.\\
&\left.+\left(\frac{x}{\sigma}\right)^{k}(\alpha-1)\alpha k-\left(\frac{x}{\sigma}\right)^{k}(\alpha-1)\alpha k\right)
\end{aligned}
\tag{S59}
$$

By performing algebraic manipulation in the last term in (S59), it can be further bounded as:

$$
\begin{aligned}
&\leq \frac{c\Gamma(\alpha+1)}{\sigma} \\
&+\sum_{k=1}^{\infty} \frac{c}{\sigma\Gamma(\alpha k+1)} \left(\sum_{n=0}^{k} \binom{k}{n} \left(\frac{x}{\sigma}\right)^{n} \left(\frac{c}{\sigma}\right)^{k-n} t^{\alpha(k-n)} (\alpha-1)\alpha k + \left(\frac{x}{\sigma}\right)^{k} \left(\left|\Gamma(\alpha)-1\right| - (\alpha-1)\alpha k \right) \right) \\
&+\sum_{k=1}^{\infty} \frac{c(\alpha-1)}{\sigma\Gamma(\alpha k+2)} \left(\sum_{n=0}^{k} \binom{k}{n} \left(\frac{x}{\sigma}\right)^{n} \left(\frac{c}{\sigma}\right)^{k-n} t^{\alpha(k-n)} (1+(\alpha-1)\alpha k) + \left(\frac{x}{\sigma}\right)^{k} (\Gamma(\alpha)-(\alpha-1)\alpha k) \right) \\
&\leq \frac{c\Gamma(\alpha+1)}{\sigma} + \frac{c}{\sigma} \sum_{k=1}^{\infty} \frac{1}{\Gamma(\alpha k+1)} \left((\alpha-1)\alpha k \left(\frac{x+ct^{\alpha}}{\sigma}\right)^{k} + \left(\frac{x}{\sigma}\right)^{k} (\mid \Gamma(\alpha)-1 \mid -(\alpha-1)\alpha k) \right) \\
&+\frac{c(\alpha-1)}{\sigma} \sum_{k=1}^{\infty} \frac{1}{\Gamma(\alpha k+2)} \left((1+(\alpha-1)\alpha k) \left(\frac{x+ct^{\alpha}}{\sigma}\right)^{k} + \left(\frac{x}{\sigma}\right)^{k} (\Gamma(\alpha)-(\alpha-1)\alpha k) \right) \\
&= \frac{c\Gamma(\alpha+1)}{\sigma} + \frac{c(\alpha-1)}{\sigma} \sum_{k=1}^{\infty} \frac{\alpha k(x+ct^{\alpha})^{k}}{\sigma^{k}\Gamma(\alpha k+1)} + \frac{c \mid \Gamma(\alpha)-1 \mid}{\sigma} \sum_{k=1}^{\infty} \frac{x^{k}}{\sigma^{k}\Gamma(\alpha k+1)} - \frac{c(\alpha-1)}{\sigma} \sum_{k=1}^{\infty} \frac{\alpha k x^{k}}{\sigma^{k}\Gamma(\alpha k+1)} \\
&+\frac{c(\alpha-1)}{\sigma} \sum_{k=1}^{\infty} \frac{(x+ct^{\alpha})^{k}}{\sigma^{k}\Gamma(\alpha k+2)} + \frac{c(\alpha-1)^{2}}{\sigma} \sum_{k=1}^{\infty} \frac{\alpha k(x+ct^{\alpha})^{k}}{\sigma^{k}\Gamma(\alpha k+2)} + \frac{\Gamma(\alpha)c(\alpha-1)}{\sigma} \sum_{k=1}^{\infty} \frac{x^{k}}{\sigma^{k}\Gamma(\alpha k+2)} \\
&-\frac{c(\alpha-1)^{2}}{\sigma} \sum_{k=1}^{\infty} \frac{\alpha k x^{k}}{\sigma^{k}\Gamma(\alpha k+2)} \\
&\leq \frac{c\Gamma(\alpha+1)}{\sigma} + \frac{c(\alpha-1)}{\sigma} \left(\left(\frac{x+ct^{\alpha}}{\sigma}\right) \sum_{k=0}^{\infty} \frac{\alpha k(x+ct^{\alpha})^{k}}{\sigma^{k}\Gamma(\alpha k+1+\alpha)} + \left(\frac{x+ct^{\alpha}}{\sigma}\right) \alpha \sum_{k=0}^{\infty} \frac{(x+ct^{\alpha})^{k}}{\sigma^{k}\Gamma(\alpha k+1+\alpha)} \right) \\
&+\frac{xc(\Gamma(\alpha)-1)}{\sigma^{2}} \sum_{k=0}^{\infty} \frac{x^{k}}{\sigma^{k}\Gamma(\alpha k+1+\alpha)} - \frac{xc(\alpha-1)}{\sigma^{2}} \sum_{k=0}^{\infty} \frac{\alpha k x^{k}}{\sigma^{k}\Gamma(\alpha k+1+\alpha)} \\
&-\frac{\alpha x c(\alpha-1)}{\sigma^{2}} \sum_{k=0}^{\infty} \frac{x^{k}}{\sigma^{k}\Gamma(\alpha k+1+\alpha)} + \frac{(x+ct^{\alpha})c(\alpha-1)}{\sigma^{2}} \sum_{k=0}^{\infty} \frac{(x+ct^{\alpha})^{k}}{\sigma^{k}\Gamma(\alpha k+2+\alpha)} \\
&+\frac{(x+ct^{\alpha})c(\alpha-1)^{2}}{\sigma^{2}} \sum_{k=0}^{\infty} \frac{\alpha k(x+ct^{\alpha})^{k}}{\sigma^{k}\Gamma(\alpha k+2+\alpha)} + \frac{(x+ct^{\alpha})c(\alpha-1)^{2}}{\sigma^{2}} \sum_{k=0}^{\infty} \frac{\alpha(x+ct^{\alpha})^{k}}{\sigma^{k}\Gamma(\alpha k+2+\alpha)} \\
&+\frac{\Gamma(\alpha)c(\alpha-1)x}{\sigma^{2}} \sum_{k=0}^{\infty} \frac{x^{k}}{\sigma^{k}\Gamma(\alpha k+2+\alpha)} - \frac{c(\alpha-1)^{2}x}{\sigma^{2}} \sum_{k=0}^{\infty} \frac{\alpha k x^{k}}{\sigma^{k}\Gamma(\alpha k+2+\alpha)} \\
&-\frac{c(\alpha-1)^{2}x}{\sigma^{2}} \sum_{k=0}^{\infty} \frac{\alpha x^{k}}{\Gamma(\alpha k+2+\alpha)}
\end{aligned}
\tag{S60}
$$

The previous bound can be further manipulated, to obtain:

$$
\frac{c\Gamma(\alpha+1)}{\sigma}+\frac{c(\alpha-1)}{\sigma}\left(\left(\frac{x+ct^{\alpha}}{\sigma}\right)\sum_{k=0}^{\infty}\frac{(\alpha k+\alpha-\alpha)(x+ct^{\alpha})^{k}}{\sigma^{k}(\alpha k+\alpha)\Gamma(\alpha k+k)}+\left(\frac{x+ct^{\alpha}}{\sigma}\right)\alpha E_{\alpha,1+\alpha}\left(\frac{x+ct^{\alpha}}{\sigma}\right)\right)
$$

$$
\frac{c\mid\Gamma(\alpha)-1\mid x}{\sigma^{2}}E_{\alpha,1+\alpha}\left(\frac{x}{\sigma}\right)-\frac{c(\alpha-1)x}{\sigma^{2}}\sum_{k=0}^{\infty}\frac{(\alpha k+\alpha-\alpha)x^{k}}{\sigma^{k}(\alpha k+\alpha)\Gamma(\alpha k+\alpha)}-\frac{c(\alpha-1)\alpha x}{\sigma^{2}}E_{\alpha,1+\alpha}\left(\frac{x}{\sigma}\right)
$$

$$
+\frac{c(\alpha-1)(x+ct^{\alpha})}{\sigma^{2}}E_{\alpha,2+\alpha}\left(\frac{x+ct^{\alpha}}{\sigma}\right)+\frac{c(\alpha-1)^{2}(x+ct^{\alpha})}{\sigma^{2}}\sum_{k=0}^{\infty}\frac{(\alpha k+\alpha+1-(1+\alpha))(x+ct^{\alpha})^{k}}{(\alpha k+\alpha+1)\Gamma(\alpha k+\alpha+1)}
$$

$$
+\frac{\alpha c(\alpha-1)^{2}(x+ct^{\alpha})}{\sigma^{2}}E_{\alpha,2+\alpha}\left(\frac{x+ct^{\alpha}}{\sigma}\right)+\frac{\Gamma(\alpha)c(\alpha-1)x}{\sigma^{2}}E_{\alpha,2+\alpha}\left(\frac{x}{\sigma}\right)
$$

$$
-\frac{c(\alpha-1)^{2}x}{\sigma^{2}}\sum_{k=0}^{\infty}\frac{(\alpha k+\alpha+1-(1+\alpha))x^{k}}{\sigma^{k}(\alpha k+\alpha+1)\Gamma(\alpha k+\alpha+1)}-\frac{c(\alpha-1)^{2}x\alpha}{\sigma^{2}}E_{\alpha,2+\alpha}\left(\frac{x}{\sigma}\right)
$$

$$
=\frac{c(\Gamma(\alpha+1))}{\sigma}+\frac{c(\alpha-1)(x+ct^{\alpha})}{\sigma^{2}}E_{\alpha,\alpha}\left(\frac{x+ct^{\alpha}}{\sigma}\right)+\frac{c(\Gamma(\alpha)-1)x}{\sigma^{2}}E_{\alpha,1+\alpha}\left(\frac{x}{\sigma}\right)
$$

$$
-\frac{c(\alpha-1)x}{\sigma^{2}}E_{\alpha,\alpha}\left(\frac{x}{\sigma}\right)+\frac{c(\alpha-1)(x+ct^{\alpha})}{\sigma^{2}}E_{\alpha,2+\alpha}\left(\frac{x+ct^{\alpha}}{\sigma}\right)+
$$

$$
\frac{c(\alpha-1)^{2}(x+ct^{\alpha})}{\sigma^{2}}\left(E_{\alpha,\alpha+1}\left(\frac{x+ct^{\alpha}}{\sigma}\right)-E_{\alpha,\alpha+2}\left(\frac{x+ct^{\alpha}}{\sigma}\right)\right)+\frac{\Gamma(\alpha)(\alpha-1)cx}{\sigma^{2}}E_{\alpha,2+\alpha}\left(\frac{x}{\sigma}\right)
$$

$$
-\frac{c(\alpha-1)^{2}x}{\sigma}\left(E_{\alpha,\alpha+1}\left(\frac{x}{\sigma}\right)-E_{\alpha,\alpha+2}\left(\frac{x}{\sigma}\right)\right).
\tag{S61}
$$

By conveniently arranging (S58) and (S61), we obtain (S57). We notice that our error estimates, provide convergence in the case of $\alpha \approx 1^{-}$. Also, there are good absolute error estimates for sufficiently small times for $\alpha \approx 1^{-}$. On the other hand, the estimates in the case of $\alpha \approx 1^{+}$ are reasonable, however, they do not converge to the fractional order derivative. In particular, we observe that as $\alpha \to 1^{+}$ the estimate is always off by an error of $\frac{c(\Gamma(\alpha+1))}{\sigma}$. Therefore, these estimates are only useful for long connectivity extents, and relatively low speeds. In Figure S2(b), we provide concise examples of error estimates that motive the analysis made in the main manuscript. We analyzed the error in the parameter ranges-of-interest and found a better approximation when considering longer connectivity ranges lying between biologically plausible ranges (1000μm to 1500 μm), with wave speed and width features at the lower limits of the observable ranges. For illustration purposes, we show, in Figure S2, the error estimates under different parameter configurations, considering wave speeds at the lower limit of observable waves. We show spatial distances of up to 3000 μm, as the error estimates rapidly increase in terms of Mittag-Leffler functions. However, with these extensions, we can capture information about a considerable part of the profile of wider waves (wave widths ranging from 3000 μm to 5000 μm).

To numerically solve all the equations of the Mittag-Leffler approximations (matching conditions and error estimates), we implemented numerical routines in Mathematica (Mathematica, 2021). In all cases, the space was discretized by considering $dx = 100$ (μm), on a bounded interval $[-2000, 5000]$ μm.

5 ADOMIAN DECOMPOSITION METHOD

In this section, we describe the use of the Adomian decomposition method to obtain the approximate fractional-order traveling wave solutions that have been established in section 3 of the main manuscript.

5.1 Approximate traveling wave solution for $0 < \alpha < 1$

We consider the fractional neural field model:

$$\begin{aligned} D_t^{\alpha} u\left(x,t\right) =& - u\left(x,t\right) + \int_{-\infty}^{\infty} g(x-y)H(u(y,t)-k)dy - \beta q\left(x,t\right) \\ D_t^{\alpha} q\left(x,t\right) =& \epsilon u\left(x,t\right) - \epsilon q\left(x,t\right), \end{aligned} \tag{S62}$$

for $0 < \alpha < 1$. The method consists of considering the integer-order traveling wave solutions as initial conditions. In order to do so, we first rewrite the traveling wave solutions established in section 2 of the main manuscript:

$$\begin{aligned} u_\star\left(x,t\right) =& \left(\frac{\epsilon - 1 + \sqrt{(\epsilon-1)^2 - 4\epsilon\beta}}{2c\sqrt{\left(\epsilon-1\right)^2 - 4\epsilon\beta}}\right) e^{\lambda_+(x+ct)} \int_{-\infty}^{x+ct} e^{-s\lambda_+} \left(\int_0^w g\left(s-y\right)dy\right) ds \\ & - \left(\frac{\epsilon - 1 - \sqrt{(\epsilon-1)^2 - 4\epsilon\beta}}{2c\sqrt{\left(\epsilon-1\right)^2 - 4\epsilon\beta}}\right) e^{\lambda_-(x+ct)} \int_{-\infty}^{x+ct} e^{-s\lambda_-} \left(\int_0^w g\left(s-y\right)dy\right) ds, \end{aligned} \tag{S63}$$

and

$$\begin{aligned} q_\star\left(x,t\right) =& \left(\frac{\epsilon}{c\sqrt{\left(\epsilon-1\right)^2 - 4\epsilon\beta}}\right) e^{\lambda_+(x+ct)} \int_{-\infty}^{x+ct} e^{-s\lambda_+} \left(\int_0^w g\left(s-y\right)dy\right) ds \\ & - \left(\frac{\epsilon}{c\sqrt{\left(\epsilon-1\right)^2 - 4\epsilon\beta}}\right) e^{\lambda_-(x+ct)} \int_{-\infty}^{x+ct} e^{-s\lambda_-} \left(\int_0^w g\left(s-y\right)dy\right) ds, \end{aligned} \tag{S64}$$

where

$$\lambda_\pm = -\frac{\epsilon+1}{2c} \pm \frac{\sqrt{\left(\epsilon-1\right)^2 - 4\epsilon\beta}}{2c}, \tag{S65}$$

and

$$(\epsilon-1)^2 - 4\epsilon\beta > 0. \tag{S66}$$

We establish the following initial conditions in our initial value problem:

$$u(x,0) = u_\star(x,0) =: U_\star(x), \tag{S67}$$

and

$$q(x,0) = q_\star(x,0) =: Q_\star(x). \tag{S68}$$

Applying the integral operator J^{α} to (S62) and considering that $J_t^{\alpha} D_t^{\alpha} u(x,t) = u(x,t) - u_{\star}(x,0)$ and $J_t^{\alpha} D_t^{\alpha} q(x,t) = q(x,t) - q_{\star}(x,0)$ for $0 < \alpha < 1$, we obtain:

$$\begin{aligned} u(x,t) - U_{\star}(x) =& - J_t^{\alpha} u\left(x,t\right) + J_t^{\alpha}\left(\int_{-\infty}^{\infty} g(x-y) H(u(y,t)-k) dy\right) - \beta J_t^{\alpha} q\left(x,t\right) \\ q(x,t) - Q_{\star}(x) =& \epsilon J_t^{\alpha} u\left(x,t\right) - \epsilon J_t^{\alpha} q\left(x,t\right). \end{aligned} \tag{S69}$$

We consider the following asymptotic expansions:

$$u_f\left(x,t\right) = \sum_{n=0}^{\infty} u_n\left(x,t\right), \tag{S70}$$

$$q_f\left(x,t\right) = \sum_{n=0}^{\infty} q_n\left(x,t\right), \tag{S71}$$

where

$$\psi(u) = \int_{-\infty}^{\infty} g(x-y) H(u(y,t)-k)) dy, \tag{S72}$$

$$\psi(u(x,t)) = \sum_{n=0}^{\infty} A_n\left(u_0, u_1, ..., u_n\right), \tag{S73}$$

and the nonlinear terms can be obtained from the following formula (Adomian, 1988; Jafari and Daftardar-Gejji, 2006; Wazwaz, 2000):

$$A_n = \frac{1}{n!}\left[\frac{d^n}{d\lambda^n}\psi\left(\sum_{n=0}^{\infty} u_n\right)\right]\Bigg|_{\lambda=0}. \tag{S74}$$

By using the previous expansions in (S69), we obtain:

$$\begin{aligned} \sum_{n=0}^{\infty} u_n\left(x,t\right) =& U_{\star}(x) - J_t^{\alpha}\sum_{n=0}^{\infty} u_n\left(x,t\right) + J_t^{\alpha}\sum_{n=0}^{\infty} A_n\left(u_0, u_1, ..., u_n\right) - \beta J_t^{\alpha}\sum_{n=0}^{\infty} q_n\left(x,t\right) \\ \sum_{n=0}^{\infty} q_n\left(x,t\right) =& Q_{\star}(x) + \epsilon J_t^{\alpha}\sum_{n=0}^{\infty} u_n\left(x,t\right) - \epsilon J_t^{\alpha}\sum_{n=0}^{\infty} q_n\left(x,t\right). \end{aligned} \tag{S75}$$

The previous system can also be expressed as:

$$\begin{aligned} u_0 + u_1 + \ldots + u_n + \ldots =& U_{\star}(x) - J_t^{\alpha} u_0 - J_t^{\alpha} u_1 - \ldots - J_t^{\alpha} u_n - \ldots \\ & + J_t^{\alpha} A_0 + J_t^{\alpha} A_1 + \ldots + J_t^{\alpha} A_n + \ldots \\ & - \beta J_t^{\alpha} q_0 - \beta J_t^{\alpha} q_1 - \ldots - \beta J_t^{\alpha} q_n - \ldots \\ q_0 + q_1 + \ldots + q_n + \ldots =& Q_{\star}(x) - \epsilon J_t^{\alpha} q_0 - \epsilon J_t^{\alpha} q_1 - \ldots - \epsilon J_t^{\alpha} q_n - \ldots \\ & + \epsilon J_t^{\alpha} u_0 + \epsilon J_t^{\alpha} u_1 + \ldots + \epsilon J_t^{\alpha} u_n + \ldots. \end{aligned} \tag{S76}$$

We proceed to equate terms recursively, obtaining:

$$\begin{aligned}
u_0(x,t) =& U_\star(x)\\
u_1(x,t) =& J_t^\alpha\left(-u_0 + A_0 - \beta q_0\right)\\
u_2(x,t) =& J_t^\alpha\left(-u_1 + A_1 - \beta q_1\right)\\
&\vdots\\
u_{n+1}(x,t) =& J_t^\alpha\left(-u_n + A_n - \beta q_n\right)\\
&\vdots\\
q_0(x,t) =& Q_\star(x)\\
q_1(x,t) =& J_t^\alpha\left(-\epsilon q_0 + \epsilon u_0\right)\\
q_2(x,t) =& J_t^\alpha\left(-\epsilon q_1 + \epsilon u_1\right)\\
&\vdots\\
q_{n+1}(x,t) =& J_t^\alpha\left(-\epsilon q_n + \epsilon u_n\right)\\
&\vdots
\end{aligned} \tag{S77}$$

The nonlinear terms can be obtained from equation (S74), as:

$$A_n = \frac{1}{n!}\left[\frac{d^n}{d\lambda^n}\left(\int_{-\infty}^{\infty} g(x-y)H\left(\sum_{k=0}^{\infty}\lambda^k u_k(y,t) - k\right)dy\right)\right]\Bigg|_{\lambda=0}. \tag{S78}$$

By substituting $n = 0$ in the previous expression, we find:

$$A_0 = \int_{-\infty}^{\infty} g(x-y)H\left(u_0 - k\right)dy = \int_0^w g(x-y)dy. \tag{S79}$$

By substituing (S79) in the recursive formula established in (S77), we obtain:

$$u_1(x,t) = \left(-U_\star(x) + \int_0^w g(x-y)dy - \beta Q_\star(x)\right)\frac{t^\alpha}{\Gamma(\alpha+1)} =: \quad f_1(x)\frac{t^\alpha}{\Gamma(\alpha+1)}, \tag{S80}$$

and

$$q_1(x,t) = \left(\epsilon U_\star(x) - \epsilon Q_\star(x)\right)\frac{t^\alpha}{\Gamma(\alpha+1)} =: \quad h_1(x)\frac{t^\alpha}{\Gamma(\alpha+1)}. \tag{S81}$$

The previous expressions, can be used in (S78) to determine the term A_1, as:

$$\begin{aligned}A_1 =& \int_{-\infty}^{\infty} g(x-y)\frac{d}{d\lambda}H\left(\lambda^k u_k(y,t)-k\right)dy\Big|_{\lambda=0}\\ =&\frac{g(x)u_1(0,t)}{\mid U_\star'(0)\mid}+\frac{g(x-w)u_1(w,t)}{\mid U_\star'(w)\mid}\\ =&\frac{g(x)f_1(0)}{\mid U_\star'(0)\mid}\frac{t^\alpha}{\Gamma(\alpha+1)}+\frac{g(x-w)f_1(w)}{\mid U_\star'(w)\mid}\frac{t^\alpha}{\Gamma(\alpha+1)}.\end{aligned} \tag{S82}$$

By substituting the previous expression into the recursive relation (S77), we obtain:

$$\begin{aligned}u_2(x,t) =& \left(-f_1(x)+\frac{g(x)f_1(0)}{\mid U_\star'(0)\mid}+\frac{g(x-w)f_1(w)}{\mid U_\star'(w)\mid}-\beta h_1(x)\right)\frac{t^{2\alpha}}{2\alpha+1}\\ =:& f_2(x)\frac{t^{2\alpha}}{\Gamma(2\alpha+1)},\end{aligned} \tag{S83}$$

and

$$\begin{aligned}q_2(x,t) =& \left(-\epsilon h_1(x)+\epsilon f_1(x)\right)\frac{t^{2\alpha}}{2\alpha+1}\\ =:& h_2(x)\frac{t^{2\alpha}}{\Gamma(2\alpha+1)}.\end{aligned} \tag{S84}$$

The previous expressions are again substituted into (S74), to obtain:

$$\begin{aligned}A_2 =& \frac{1}{2!}\left[\frac{d^2}{d\lambda^2}\left(\int_{-\infty}^{\infty} g(x-y)H\left(\sum_{k=0}^{\infty}\lambda^k u_k(y,t)-k\right)dy\right)\right]\Big|_{\lambda=0}\\ =&\frac{1}{2}\left(\frac{2g(x)f_2(0)}{\mid U_\star'(0)}\frac{t^{2\alpha}}{\Gamma(2\alpha+1)}+\frac{2g(x-w)f_2(w)}{\mid U_\star'(w)}\frac{t^{2\alpha}}{\Gamma(2\alpha+1)}\right.\\ &\left.-\frac{1}{\mid U_\star'(0)\mid}\frac{\partial}{\partial y}\left(g(x-y)f_1^2(y)\right)\Big|_{y=0}\frac{t^{2\alpha}}{(\Gamma(\alpha+1))^2}-\frac{1}{\mid U_\star'(w)\mid}\frac{\partial}{\partial y}\left(g(x-y)f_1^2(y)\right)\Big|_{y=w}\frac{t^{2\alpha}}{(\Gamma(\alpha+1))^2}\right).\end{aligned} \tag{S85}$$

The previous expression can be used to determine the terms u_3 and q_3 from the recursive relation established in (S77). Once the terms u_3 and q_3 are determined, the relation (S78) can be used to determine A_3, and formula (S77) can be used again to determine the remaining recursive terms. Here, we present the approximate traveling wave solutions using a 4α approximation:

$$\begin{aligned}u_f(x,t) \approx& U_\star(x)+f_1(x)\frac{t^\alpha}{\Gamma(\alpha+1)}+f_2(x)\frac{t^{2\alpha}}{\Gamma(2\alpha+1)}+f_3(x)\frac{t^{3\alpha}}{\Gamma(3\alpha+1)}\\ &+f_4(x)\frac{t^{4\alpha}}{\Gamma(4\alpha+1)}\end{aligned}, \tag{S86}$$

and

$$
\begin{aligned}
q_f\left(x,t\right) \approx Q_\star\left(x\right) + h_1\left(x\right)\frac{t^{\alpha}}{\Gamma\left(\alpha+1\right)} + h_2\left(x\right)\frac{t^{2\alpha}}{\Gamma\left(2\alpha+1\right)} + h_3\left(x\right)\frac{t^{3\alpha}}{\Gamma\left(3\alpha+1\right)} \\
+ h_4\left(x\right)\frac{t^{4\alpha}}{\Gamma\left(4\alpha+1\right)}.
\end{aligned} \tag{S87}
$$

The different terms of the previous expressions are determined by:

$$
f_1\left(x\right) = \left(-U_\star\left(x\right) + \int_0^w g(x-y)dy - \beta Q_\star\left(x\right)\right), \tag{S88}
$$

$$
f_2\left(x\right) = \left(-f_1\left(x\right) - \beta h_1(x) + \frac{g(x)f_1(0)}{\mid U_\star'(0)\mid} + \frac{g(x-w)f_1(w)}{\mid U_\star'(w)\mid}\right), \tag{S89}
$$

$$
\begin{aligned}
f_3\left(x\right) = \Bigg(-f_2\left(x\right) - \beta h_2(x) + \frac{g(x)f_2(0)}{\mid U_\star'(0)\mid} + \frac{g(x-w)f_2(w)}{\mid U_\star'(w)\mid} \\
-\frac{\Gamma(2\alpha+1)}{2!(\Gamma(\alpha+1))^2}\times\left(\sum_{k=\{0,w\}}\frac{1}{\mid U_\star'(k)\mid}\frac{\partial}{\partial y}\left(g(x-y)f_1^2(y)\right)\Bigg|_{y=k}\right)\Bigg),
\end{aligned} \tag{S90}
$$

$$
\begin{aligned}
f_4\left(x\right) = \Bigg(-f_3(x) - \beta h_3(x) + \frac{g(x)f_3(0)}{\mid U_\star'(0)\mid} + \frac{g(x-w)f_3(w)}{\mid U_\star'(w)\mid} \\
-\frac{\Gamma(3\alpha+1)}{\Gamma(2\alpha+1)\Gamma(\alpha+1)}\times\left(\sum_{k=\{0,w\}}\frac{1}{\mid U_\star'(k)\mid}\frac{\partial}{\partial y}\left(g(x-y)f_2(y)f_1(y)\right)\Bigg|_{y=k}\right) \\
+\frac{\Gamma(3\alpha+1)}{3!(\Gamma(\alpha+1))^3}\times\left(\sum_{k=\{0,w\}}\frac{1}{\mid U_\star'(k)\mid}\frac{\partial^2}{\partial y^2}\left(g(x-y)(f_1(y))^3\right)\Bigg|_{y=k}\right)\Bigg),
\end{aligned} \tag{S91}
$$

$$
h_1\left(x\right) = -\epsilon Q_\star\left(x\right) + \epsilon U_\star\left(x\right), \tag{S92}
$$

and

$$
h_j\left(x\right) = -\epsilon h_{j-1}\left(x\right) + \epsilon f_{j-1}\left(x\right) \tag{S93}
$$

for $j \geq 2, j \in \mathbb{Z}^+$. In the previous expressions, we are assuming that $U_\star(x)$ crossed the threshold at $x = 0$ and $x = w$.

We proceed to establish the approximate traveling wave solutions for the case of $1 < \alpha < 2$.

5.2 Traveling wave solution for $1 < \alpha < 2$

We consider the fractional neural field model (S62), in the case of $1 < \alpha < 2$. We now establish the following initial conditions:

$$
u(x,0) = u_\star(x,0), \tag{S94}
$$

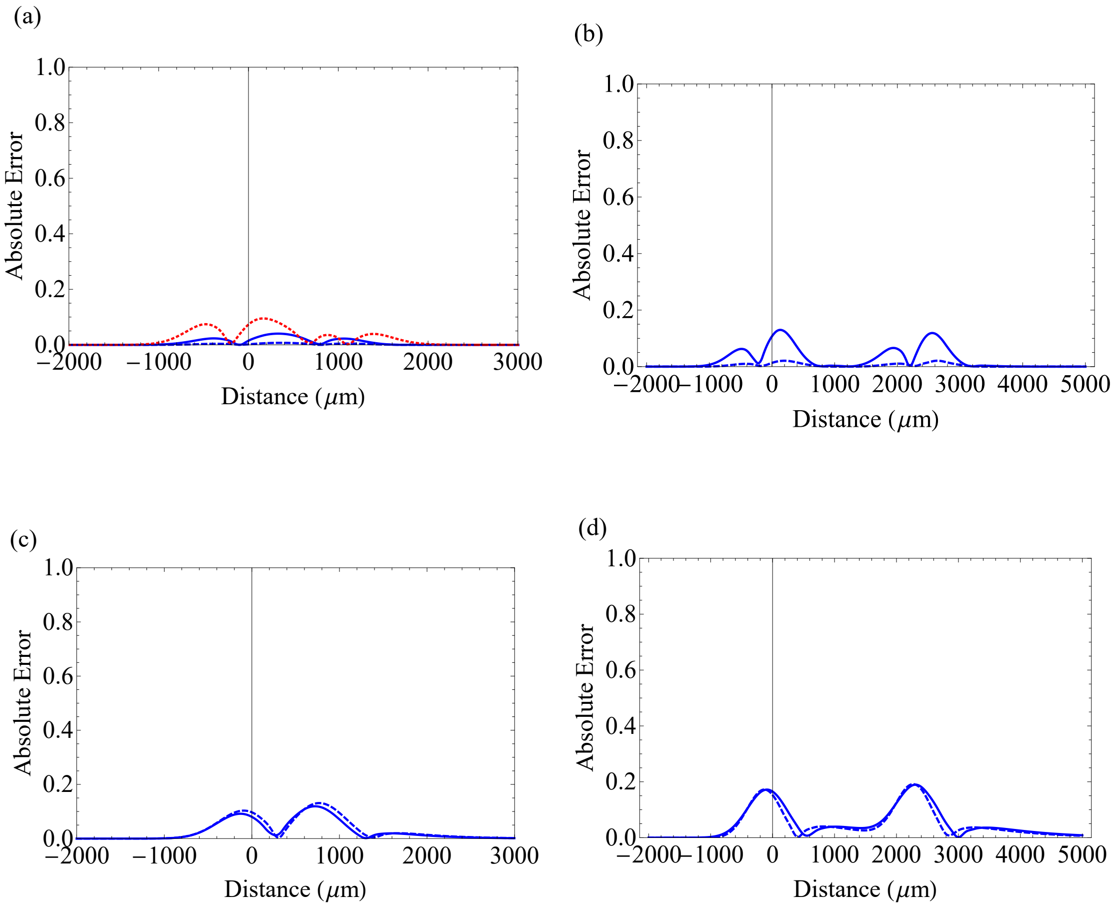

Figure S3. Absolute error estimates for the Adomian Decomposition Method considering the integer-order explicit solution. The dashed blue lines correspond to $t = 1$, the blue lines correspond to $t = 2$ and the red dashed lines correspond to $t = 3.5$ (only for (a)). The error increases as time increases and it also depends on wave width and therefore, synaptic threshold. We compute the error estimate for two wave solutions labelled as "red square wave solution" and "red circle wave solution" in the main manuscript (Figure 5). The features for the "red circle" wave solution are: the wave speed is $c = 110\ \mu m/ms$, the wave width is $w = 847\ \mu m/m$ and the synaptic threshold is $k = 0.33$. The features for the "red square" wave solution are: the wave speed is $c = 202\ \mu m/ms$, the wave width is $w = 2413\ \mu m/ms$ and the synaptic threshold is $k = 0.28$. **(a)** We consider $0 < \alpha < 1$ and the red circle wave solution. **(b)** We consider $0 < \alpha < 1$ and the red square wave solution. **(c)** We consider $1 < \alpha < 2$ and the red circle wave solution. **(b)** We consider $1 < \alpha < 2$ and the red square wave solution. Parameters fixed for all the plots: $\sigma = 300$, $\epsilon = 0.1$, $\beta = 1$ and $\alpha = 1$.

$$u_t(x,0) = \frac{\partial u_\star}{\partial t}(x,0), \tag{S95}$$

$$q(x,0) = q_\star(x,0), \tag{S96}$$

and

$$q_t(x,0) = \frac{\partial q_\star}{\partial t}(x,0). \tag{S97}$$

Applying the integral operator J^{α} to (S62) and considering that $J^{\alpha}D_t^{\alpha}u(x,t) = u(x,t) - u_{\star}(x,0) - \frac{\partial u_{\star}}{\partial t}(x,0)$ and $J^{\alpha}D_t^{\alpha}q(x,t) = q(x,t) - q_{\star}(x,0) - \frac{\partial q_{\star}}{\partial t}(x,0)$ for $1 < \alpha < 2$, we obtain:

$$
\begin{aligned}
u(x,t) - u_{\star}(x,0) - \frac{\partial u_{\star}}{\partial t}(x,0) =& - J_t^{\alpha}u\,(x,t) + J_t^{\alpha}\left(\int_{-\infty}^{\infty} g(x-y)H(u(y,t)-k)dy\right) - \beta J_t^{\alpha}q\,(x,t) \\
q(x,t) - q_{\star}(x,0) - \frac{\partial q_{\star}}{\partial t}(x,0) =& \epsilon J_t^{\alpha}u\,(x,t) - \epsilon J_t^{\alpha}q\,(x,t)\,.
\end{aligned}
\tag{S98}
$$

We consider the following asymptotic expansions:

$$
u_f\,(x,t) = \sum_{n=0}^{\infty} u_n\,(x,t)\,, \tag{S99}
$$

$$
q_f\,(x,t) = \sum_{n=0}^{\infty} q_n\,(x,t)\,, \tag{S100}
$$

where

$$
\psi(u) = \int_{-\infty}^{\infty} g(x-y)H(u(y,t)-k))dy, \tag{S101}
$$

$$
\psi(u(x,t)) = \sum_{n=0}^{\infty} A_n\,(u_0, u_1, ..., u_n)\,, \tag{S102}
$$

and the nonlinear terms can also be obtained from equation (S74). By using the previous expansions in (S98), we obtain:

$$
\begin{aligned}
\sum_{n=0}^{\infty} u_n\,(x,t) =& u_{\star}(x,0) + \frac{\partial u_{\star}}{\partial t}(x,0) - J_t^{\alpha}\sum_{n=0}^{\infty} u_n\,(x,t) + J_t^{\alpha}\sum_{n=0}^{\infty} A_n\,(u_0, u_1, ..., u_n) - \beta J_t^{\alpha}\sum_{n=0}^{\infty} q_n\,(x,t) \\
\sum_{n=0}^{\infty} q_n\,(x,t) =& q_{\star}(x,0) + \frac{\partial q_{\star}}{\partial t}(x,0) + \epsilon J_t^{\alpha}\sum_{n=0}^{\infty} u_n\,(x,t) - \epsilon J_t^{\alpha}\sum_{n=0}^{\infty} q_n\,(x,t)\,.
\end{aligned}
\tag{S103}
$$

The previous system can also be expressed as:

$$
\begin{aligned}
u_0+u_1+\ldots+u_n+\ldots=&u_\star(x,0)+\frac{\partial u_\star}{\partial t}(x,0)-J_t^\alpha u_0-J_t^\alpha u_1-\ldots-J_t^\alpha u_n-\ldots\\
&+J_t^\alpha A_0+J_t^\alpha A_1+\ldots+J_t^\alpha A_n+\ldots\\
&-\beta J_t^\alpha q_0-\beta J_t^\alpha q_1-\ldots-\beta J_t^\alpha q_n-\ldots\\
q_0+q_1+\ldots+q_n+\ldots=&q_\star(x,0)+\frac{\partial q_\star}{\partial t}(x,0)-\epsilon J_t^\alpha q_0-\epsilon J_t^\alpha q_1-\ldots-\epsilon J_t^\alpha q_n-\ldots\\
&+\epsilon J_t^\alpha u_0+\epsilon J_t^\alpha u_1+\ldots+\epsilon J_t^\alpha u_n+\ldots.
\end{aligned} \tag{S104}
$$

We proceed to equate terms recursively, obtaining:

$$
\begin{aligned}
u_0(x,t)=&u_\star(x,0)+\frac{\partial u_\star}{\partial t}(x,0)\\
u_1(x,t)=&J_t^\alpha\left(-u_0+A_0-\beta q_0\right)\\
u_2(x,t)=&J_t^\alpha\left(-u_1+A_1-\beta q_1\right)\\
&\vdots\\
u_{n+1}(x,t)=&J_t^\alpha\left(-u_n+A_n-\beta q_n\right)\\
&\vdots\\
q_0(x,t)=&q_\star(x,0)+\frac{\partial q_\star}{\partial t}(x,0)\\
q_1(x,t)=&J_t^\alpha\left(-\epsilon q_0+\epsilon u_0\right)\\
q_2(x,t)=&J_t^\alpha\left(-\epsilon q_1+\epsilon u_1\right)\\
&\vdots\\
q_{n+1}(x,t)=&J_t^\alpha\left(-\epsilon q_n+\epsilon u_n\right)\\
&\vdots
\end{aligned} \tag{S105}
$$

The nonlinear terms can also be obtained from equation (S74), as is established in (S78). However, in the case of $\alpha \approx 1^+$, the points where activity cross the synaptic threshold are determined by values w_a and w_b such that $u_\star(w_a,0)+\frac{\partial u_\star}{\partial t}(w_a,0)=u_\star(w_b,0)+\frac{\partial u_\star}{\partial t}(w_b,0)=k$. It is possible to follow the same recursive approach that has been developed in Section 5.1, to obtain the terms in (S105). Here, we present the wave solutions using a 4α approximation:

$$
\begin{aligned}
u_f(x,t)\approx U_0(x)+f_1(x)\frac{t^\alpha}{\Gamma(\alpha+1)}+f_2(x)\frac{t^{2\alpha}}{\Gamma(2\alpha+1)}+f_3(x)\frac{t^{3\alpha}}{\Gamma(3\alpha+1)}\\
+f_4(x)\frac{t^{4\alpha}}{\Gamma(4\alpha+1)}
\end{aligned}, \tag{S106}
$$

and

$$q_f\left(x,t\right) \approx Q_0(x) + h_1\left(x\right)\frac{t^{\alpha}}{\Gamma\left(\alpha+1\right)} + h_2\left(x\right)\frac{t^{2\alpha}}{\Gamma\left(2\alpha+1\right)} + h_3\left(x\right)\frac{t^{3\alpha}}{\Gamma\left(3\alpha+1\right)} + h_4\left(x\right)\frac{t^{4\alpha}}{\Gamma\left(4\alpha+1\right)}, \tag{S107}$$

where the previous terms are determined by:

$$U_0\left(x\right) = u_\star(x,0) + \frac{\partial u_\star}{\partial t}(x,0), \tag{S108}$$

$$Q_0\left(x\right) = q_\star(x,0) + \frac{\partial q_\star}{\partial t}(x,0), \tag{S109}$$

$$f_1\left(x\right) = \left(-U_0(x) + \int_{w_a}^{w_b} g(x-y)dy - \beta Q_0\left(x\right)\right), \tag{S110}$$

$$f_2\left(x\right) = \left(-f_1\left(x\right) - \beta h_1(x) + \frac{g(x-w_a)f_1(w_a)}{\mid U_0'(w_a)\mid} + \frac{g(x-w_b)f_1(w_b)}{\mid U_0'(w_b)\mid}\right), \tag{S111}$$

$$f_3\left(x\right) = \left(-f_2\left(x\right) - \beta h_2(x) + \frac{g(x-w_a)f_2(w_a)}{\mid u_0'(w_a)\mid} + \frac{g(x-w_b)f_2(w_b)}{\mid u_0'(w_b)\mid} - \frac{\Gamma(2\alpha+1)}{2!(\Gamma(\alpha+1))^2} \times \left(\sum_{k=\{w_a,w_b\}} \frac{1}{\mid U_0'(j)\mid}\frac{\partial}{\partial y}\left(g(x-y)f_1^2(y)\right)\Big|_{y=k}\right)\right), \tag{S112}$$

$$f_4\left(x\right) = \left(-f_3(x) - \beta h_3(x) + \frac{g(x)f_3(w_a)}{\mid U_0'(w_a)\mid} + \frac{g(x-w_b)f_3(w_b)}{\mid U_0'(w_b)\mid} - \frac{\Gamma(3\alpha+1)}{\Gamma(2\alpha+1)\Gamma(\alpha+1)} \times \left(\sum_{k=\{w_a,w_b\}} \frac{1}{\mid U_0'(k)\mid}\frac{\partial}{\partial y}\left(g(x-y)f_2(y)f_1(y)\right)\Big|_{y=k}\right) + \frac{\Gamma(3\alpha+1)}{3!(\Gamma(\alpha+1))^3} \times \left(\sum_{k=\{w_a,w_b\}} \frac{1}{\mid U_0'(k)\mid}\frac{\partial^2}{\partial y^2}\left(g(x-y)(f_1(y))^3\right)\Big|_{y=k}\right)\right), \tag{S113}$$

$$h_1\left(x\right) = -\epsilon Q_0\left(x\right) + \epsilon U_0\left(x\right), \tag{S114}$$

and

$$h_j\left(x\right) = -\epsilon h_{j-1}\left(x\right) + \epsilon f_{j-1}\left(x\right), \tag{S115}$$

for $j \geq 2, j \in \mathbb{Z}^+$. In the previous expressions, we are also assuming that the function $U_0(x)$ crossed the threshold at the points $x = w_a$ and w_b.

In Figure S3, we analyze the error in the Adomian Decomposition Method. To that end, we compare the explicit solution in the integer order case to the Adomian Decomposition fourth-order approximation. We find that the approximation is better for relatively narrow waves. As the width of the wave increases the

error in the approximation increases. However, our approximation are suitable to analyze waves lying in the upper branch of solutions. In this way, we can compare our explicit approximate solutions with the solutions in the Adomian Decomposition Method obtaining consistent initial results. The Adomian Decomposition Method approximations provide better error estimates in the case $0 < \alpha < 1$ compared to $1 < \alpha < 2$. This is consistent with the error estimates obtained from the approximate traveling wave solutions and it is expected due to the different fractional order derivative definitions obtained in the case $0 < \alpha < 1$ and $1 < \alpha < 2$.

The numerical implementation of the Adomian Decomposition Approximations was performed in Mathematica (Mathematica, 2021). A routine was written to numerically solve equations (S86),(S87),(S106) and (S107) by computing each of the auxiliar functions $f_j(x)$ and $h_j(x)$. For this routine we use the Numerical Calculus package of Mathematica (Mathematica, 2021). To obtain the estimates on width and speed of wave propagation we first start the propagation at $t = 0.01$ and then we start measuring the wave speed and width (at $t = 0.1$) by fixing a consecutive time-step of $dt = 0.1$. The speed was measured at the front of the wave (leftmost point that achieves the synaptic threshold), by calculating the distance traveled divided by the correspondent time interval. The width was measured at each time step by determining the difference of the points where the wave achieved its synaptic threshold. These points were determined by a numerical routine solving a nonlinear equation. The space was discretized by considering $dx = 100$ (μm), on a bounded interval $[-2000, 5000]$ μm.

REFERENCES

Adomian, G. (1988). A review of the decomposition method in applied mathematics. *J Math Anal Appl* 135, 501–544

Baleanu, D., Diethelm, E., Scalas, E., and Trujillo, J. (2016). *Fractional calculus: models and numerical methods* (Singapore: World Scientific)

Ishteva, M. (2005). *Properties and applications of the Caputo fractional operator*. Master's thesis, Universität Karlsruhe (TH), Bulgaria

Jafari, H. and Daftardar-Gejji, V. (2006). Solving linear and nonlinear fractional diffusion and wave equations by adomian decomposition. *Appl Math Comput* 180, 488–497

Magin, R. L. (2004). Fractional calculus in bioengineering, part 1. *Critical Reviews in Biomedical Engineering* 32

Mathematica (2021). Wolfram research inc. *Mathematica, Version 12.3.1* Champaign, IL

Ortigueira, M. (2011). *Fractional Calculus for Scientists and Engineers* (Netherlands: Springer)

Podlubny, I. (1999). *Fractional Differential Equations* (USA: Academic Press)

Ross, B. (1974). A brief history and exposition of the fundamental theory of fractional calculus. In *Fractional Calculus and its applications*, ed. B. Ross (Berlin, Heidelberg: Springer-Verlag)

Wang, J. and Li, H. (2011). Surpassing the fractional derivative: Concept of the memory-dependent derivative. *Comput Math Appl* 62, 1562–1567

Wazwaz, A. (2000). A new algorithm for calculating adomian polynomials for nonlinear operators. *Appl Math Comput* 111, 53–69
